# Diverse lipid conjugates for functional extra-hepatic siRNA delivery *in vivo*

**DOI:** 10.1093/nar/gky1239

**Published:** 2018-12-14

**Authors:** Annabelle Biscans, Andrew Coles, Reka Haraszti, Dimas Echeverria, Matthew Hassler, Maire Osborn, Anastasia Khvorova

**Affiliations:** 1 RNA Therapeutics Institute, University of Massachusetts Medical School, Worcester, MA 01604, USA; 2 Program in Molecular Medicine, University of Massachusetts Medical School, Worcester, MA 01604, USA

## Abstract

Small interfering RNA (siRNA)-based therapies are proving to be efficient for treating liver-associated disorders. However, extra-hepatic delivery remains challenging, limiting therapeutic siRNA utility. We synthesized a panel of fifteen lipid-conjugated siRNAs and systematically evaluated the impact of conjugate on siRNA tissue distribution and efficacy. Generally, conjugate hydrophobicity defines the degree of clearance and the liver-to-kidney distribution profile. In addition to primary clearance tissues, several conjugates achieve significant siRNA accumulation in muscle, lung, heart, adrenal glands and fat. Oligonucleotide distribution to extra-hepatic tissues with some conjugates was significantly higher than with cholesterol, a well studied conjugate, suggesting that altering conjugate structure can enhance extra-hepatic delivery. These conjugated siRNAs enable functional gene silencing in lung, muscle, fat, heart and adrenal gland. Required levels for productive silencing vary (5–200 μg/g) per tissue, suggesting that the chemical nature of conjugates impacts tissue-dependent cellular/intracellular trafficking mechanisms. The collection of conjugated siRNA described here enables functional gene modulation *in vivo* in several extra-hepatic tissues opening these tissues for gene expression modulation. A systemic evaluation of a panel of conjugated siRNA, as reported here, has not previously been investigated and shows that chemical engineering of lipid siRNAs is essential to advance the RNA therapeutic field.

## INTRODUCTION

Therapeutic oligonucleotides—i.e. antisense oligonucleotides (ASOs), small interfering RNA (siRNA) and aptamers—are emerging as a new class of drugs in addition to small molecules and biologics (1). Their advantages over conventional drugs include: (i) ease of design—rationally achieved based on sequence information and straightforward screening, leading to drug candidates within short periods of time; (ii) ability to target disease genes previously considered ‘undruggable’; and (iii) unprecedented potency and duration of effect (2,3). Clinical success is dependent on their efficient delivery to disease tissues.

Conjugate-mediated delivery is emerging as the clinically dominant delivery paradigm for siRNAs. This mode of delivery requires full chemical stabilization of siRNAs (4–6) because unmodified siRNAs are rapidly degraded and cleared from the circulation by kidney filtration, leading to minimal bioavailability in tissues (7). Chemical scaffolds that replace every 2′ hydroxyl (8–10), modify terminal nucleotide linkages (11,12) and stabilize the 5′ phosphate (13–16) maximize the *in vivo* activity of siRNAs.

For liver delivery, the trivalent N-acetylgalactosamine (GalNAc) conjugate binds with high specificity and affinity to the asialoglycoprotein receptor on hepatocytes, resulting in specific oligonucleotide delivery to and robust gene silencing in hepatocytes (17–20). This delivery strategy has revolutionized the development of oligonucleotide therapeutics to treat liver diseases, with more than a dozen clinical programs (21). The success of the GalNAc platform demonstrates that functional tissue delivery of therapeutic oligonucleotides is a foundation for any clinical exploration.

Another class of conjugates widely used for improving siRNA delivery is lipids (22). The majority of studied lipid-conjugated siRNAs, such as cholesterol-conjugated siRNAs, accumulate in the liver (∼60–80%) (7,23,24). However, cholesterol-modified siRNAs also exhibit accumulation and productive silencing in kidney (25), muscle (26) and placenta (27). Moreover, local injection of cholesterol-modified siRNA leads to functional gene silencing in brain, vagina and skin (28–31). The impact of other lipid conjugates on extra-hepatic siRNA delivery has never been evaluated.

Here we systematically evaluate how different lipid conjugates impact siRNA tissue distribution and silencing activity *in vivo*. We synthesized a panel of siRNAs conjugated to 15 different lipid moieties, including saturated and non-saturated fatty acids, steroids and vitamins, with or without a phosphocholine polar head group. In general, the degree of siRNA hydrophobicity correlates with accumulation in kidneys (less hydrophobic) or liver (more hydrophobic). Though most of the injected siRNAs accumulate in clearance organs, we identified conjugates that enable functional siRNA delivery to heart, lung, fat, muscle and adrenal gland. In these tissues, accumulation levels were 3- to 10-fold higher than cholesterol-conjugated siRNAs. The siRNA tissue concentrations required for productive silencing varied depending on the tissue and conjugate, with no perfect correlation between compound accumulation and efficacy being observed. Our findings provide proof of principle that lipid engineering is a viable path for improving extra-hepatic delivery and target silencing.

## MATERIALS AND METHODS

### Synthesis of lipid functionalized solid support for the preparation of conjugated siRNAs

Lipid moieties (except α-tocopheryl succinate) were directly attached via a peptide bond to a controlled pore glass (CPG) functionalized by a C7 linker, as described (32). To synthesize phosphocholine derivatives, amino C7 CPG was first functionalized with phosphocholine (33). Briefly, Fmoc-L-serine tert-butyl (TCI America) was phosphitylated using 2′-cyanoethyl-N,N-diisopropylchlorophosphoramidite (ChemGenes). The resulting phosphoramidite was coupled to choline p-toluenesulfonate (Alfa Aesar) using 5-(ethylthio)-1H-tetrazole (ETT) as an activator. The phosphine ester was then oxidized, and the carboxylic acid and phosphate ester groups were deprotected (i.e. tert-butyl and cyanoethyl groups removed). The resulting intermediate was attached to the amino C7 CPG via a peptide bond to form phosphocholine-functionalized CPG. The Fmoc group was removed, and the selected lipid moiety was attached via a peptide bond to the CPG. All lipid-functionalized solid supports were obtained with a loading of 55 μmol/g.

### Synthesis of α-tocopheryl succinate-conjugated siRNAs

α-tocopheryl succinate was attached to the amino group at the 3′ ends of siRNA sense strands after synthesis, deprotection, and purification of sense strands synthesized on amino C7 CPG or phosphocholine-functionalized amino C7 CPG. *N*-hydroxysuccinimide α-tocopheryl succinate and purified sense strands were combined in a solution of 0.1 M sodium bicarbonate, 20% (v/v) dimethylformamide and incubated overnight at room temperature. One-tenth volume 3 M sodium acetate (pH 5.2) was added to obtain a final concentration of 0.3 M sodium acetate. Three volumes 95% (v/v) ethanol were added, and the mixture was vortexed and then placed for 1h at −80°C. The solution was pelleted by centrifugation for 30 min at 5200 × *g*. The pellet containing the lipid-conjugated siRNA sense strand was dissolved in water, purified and desalted as described below.

### Oligonucleotide synthesis

An Expedite ABI DNA/RNA synthesizer was used to synthesize oligonucleotides following standard protocols. Sense strands were synthesized at 10 μmole scales on lipid-functionalized CPG or amino C7 CPG (for the post-synthetic conjugation of α-tocopheryl succinate moiety) supports. Antisense strands were synthesized at 10 μmole scales on CPG functionalized with Unylinker^®^ (ChemGenes, Wilmington, MA). 2′-*O*-methyl phosphoramidites (ChemGenes, Wilmington, MA, USA), 2′-fluoro phosphoramidites (BioAutomation, Irving, TX, USA), Cy3-labeled phosphoramidites (Gene Pharma, Shanghai, China) and custom synthesized (E)-vinylphosphonate phosphoramidites (33) were prepared as 0.15 M solutions in dry acetonitrile. Trityl groups were removed using 3% dichloroacetic acid (DCA) in dichloromethane for 80 s, phosphoramidites were coupled using 0.25 M 5-(benzylthio)-1H-tetrazole (BTT) in acetonitrile as an activator for 250 s and uncoupled monomers were capped with 16% N-methylimidazole in tetrahydrofuran (CAP A) and 80:10:10 (v/v/v) tetrahydrofuran:acetic anhydride:2,6-lutidine (CAP B) for 15 s. Sulfurizations were carried out with 0.1 M solution of 3-[(dimethylaminomethylene)amino]-3H-1,2,4-dithiazole-5-thione (DDTT) in acetonitrile for 3 min. Oxidation was performed in 0.02 M iodine in tetrahydrofuran:pyridine:water (70:20:10, v/v/v) for 80 s.

### Deprotection and purification of oligonucleotides

Sense strands were cleaved and deprotected using 1 ml of 40% aq. methylamine at 45°C for 1 h. Antisense strands were first deprotected with a solution of bromotrimethylsilane:pyridine (3:2, v/v) in dichloromethane (5 ml) for the (E)-vinylphosphonate deprotection and then cleaved and deprotected with 10 ml of 40% aq. methylamine at 45°C for 1 h. Sense and antisense oligonucleotide solutions were frozen in liquid nitrogen for a few minutes and dried overnight in a Speedvac under vacuum. The resulting pellets were suspended in water and purified using an Agilent Prostar System (Agilent, Santa Clara, CA, USA). Sense strands were purified over a Hamilton HxSil C18 column (150 × 21.2) in a continuous gradient of sodium acetate: 90% Buffer A1 (50 mM sodium acetate in 5% acetonitrile), 10% Buffer B1 (acetonitrile) to 10% Buffer A1, 90% Buffer B1 at a flow rate of 5 ml/min for 18 min at 70°C. Antisense strands were purified over a Dionex NucleoPac PA-100 (9 × 250) in a continuous gradient of sodium percholate: 100% Buffer A2 (30% acetonitrile in water) to 20% Buffer A2, 80% Buffer B2 (1 M sodium perchlorate, 30% acetonitrile) at a flow rate of 10 ml/min for 30 min at 65°C. Purified oligonucleotides were collected, desalted by size-exclusion chromatography using a Sephadex G25 column (GE Healthcare Life Sciences, Marlborough, MA, USA) and lyophilized.

### Analysis of oligonucleotides

Oligonucleotide purity and identity was determined by Liquid Chromatography-Mass Spectrometry (LC-MS) analysis on an Agilent 6530 accurate-mass Q-TOF LC/MS (Agilent technologies, Santa Clara, CA, USA). Liquid chromatography was performed using a 2.1 × 50-mm AdvanceBio oligonucleotide column (Agilent technologies, Santa Clara, CA) and a gradient of buffers A (9 mM trimethylamine, 100 mM hexafluoroisopropanol in water) and B (9 mM triethylamine/100 mM hexafluoroisopropanol in methanol). Sense strand gradient: 1% B to 40% B from 0 to 2 min, 40% B to 100% B from 2 to 10.5 min. Antisense strand gradient: 1% B to 12% B from 0 to 2 min, 12% B to 30% B from 2 to 10.5 min and 30% B to 100% B from 10.5 to 11 min. Relative hydrophobicities of conjugated siRNAs were determine by measuring the retention time on an Agilent Prostar System equipped with a Water HxSil C18 column (75 × 4.6) in a gradient of 100% buffer A (0.1 M trimethylamine acetate in water) to 100% buffer B (0.1 M trimethylamine acetate in acetonitrile) at a flow rate of 1 ml/min for 15 min at 60°C.

### Injection of conjugated siRNAs into mice

Animal experiments were performed in accordance with animal care ethics approval and guidelines of University of Massachusetts Medical School Institutional Animal Care and Use Committee (IACUC, protocol number A-2411). Female FB/NJ mice (The Jackson Laboratory) 7- to 8-weeks old were injected subcutaneously with phosphate buffered saline (PBS controls) or with 20 mg/kg siRNA (unconjugated or lipid-conjugated) suspended in phosphate-buffered saline (PBS) (160 μl). For distribution studies, three mice per conjugate were injected (*n* = 49, including controls). For the efficacy studies, eight mice per conjugate, per gene were studied (*n* = 272, including controls). For the toxicity studies, three mice per conjugate were injected (*n* = 30, including controls).

### Fluorescence microscopy

At 48 h post-injection, mice were euthanized and perfused with PBS. Tissues were collected and immersed in 10% formalin solution overnight at 4°C. Tissues were embedded in paraffin and sliced into 4-μm sections that were mounted on glass slides. Tissue sections on glass slides were deparaffinized by incubating twice in xylene for 8 min. Sections were rehydrated in an ethanol series from 100% to 95% to 80%, for 4 min each. Slides were then washed twice with PBS, 2 min each, incubated with DAPI (250 ng/ml, Molecular Probes) in PBS for 1 min and washed again in PBS for 2 min. Slides were mounted with PermaFluor mounting medium (Molecular Probes) coverslips, and dried overnight at 4°C. Sections were imaged at 5 × and 40 × using a Leica DM5500B microscope fitted with a DFC365 FX fluorescence camera.

### Peptide nucleic acid (PNA) hybridization assay

Tissue concentrations of antisense strands were determined using a peptide nucleic acid (PNA) hybridization assay (34,35). Tissues (15 mg) were placed in QIAGEN Collection Microtubes holding 3-mm tungsten beads and lysed in 300 μl MasterPure tissue lysis solution (EpiCentre) containing 0.2 mg/ml proteinase K (Invitrogen) using a QIAGEN TissueLyser II. Lysates were then centrifuged at 1000 × *g* for 10 min and incubated for 1 h at 55° to 60°C. Sodium dodecyl sulphate (SDS) was precipitated from lysates by adding 20 μl 3 M potassium chloride and pelleted centrifugation at 5000 × *g* for 15 min. Conjugated siRNAs in cleared supernatant were hybridized to a Cy3-labeled PNA probe fully complementary to the antisense strand (PNABio, Thousand Oaks, CA, USA). Samples were analyzed by HPLC (Agilent, Santa Clara, CA, USA) over a DNAPac PA100 anion-exchange column (Thermo Fisher Scientific), in a gradient of sodium perchlorate, as follows: Buffer A: 50% water; 50% acetonitrile; 25 mM Tris–HCl, pH 8.5; 1 mM ethylenediaminetetraacetate. Buffer B: 800 mM sodium perchlorate in buffer A. Gradient conditions: 10% buffer B within 4 min, 50% buffer B for 1 min and 50% to 100% buffer B within 5 min. Cy3 fluorescence was monitored and peaks integrated. Final concentrations were ascertained using calibration curves generated by spiking known quantities of lipid-conjugated siRNA into tissue lysates from an untreated animal. Spiked samples for calibration and experimental samples were processed and analyzed under the same laboratory conditions.

### *In vivo* mRNA silencing experiments

At 1-week post-injection, mice were euthanized and perfused with PBS. Tissues were collected and stored in RNAlater (Sigma) at 4°C overnight. mRNA was quantified using the QuantiGene 2.0 Assay (Affymetrix). The 1.5-mm punches (three punches per tissue) were placed in QIAGEN Collection Microtubes holding 3-mm tungsten beads and lysed in 300 μl Homogenizing Buffer (Affymetrix) containing 0.2 mg/ml proteinase K (Invitrogen) using a QIAGEN TissueLyser II. Samples were then centrifuged at 1000 × *g* for 10 min and incubated for 1 h at 55° to 60°C. Lysates and diluted probe sets (mouse *Htt*, mouse *Ppib* or mouse *Hprt*) were added to the bDNA capture plate and signal was amplified and detected as described by Coles *et al.* (36). Luminescence was detected on a Tecan M1000 (Tecan, Morrisville, NC, USA).

### Statistical analysis

Data were analyzed using GraphPad Prism 7.01 software (GraphPad Software, Inc., San Diego, CA, USA). For each independent efficacy experiment in mice, the level of silencing was normalized to the mean of the control (PBS) group. Data were analyzed using non-parametric one-way ANOVA with Bonferroni's test for multiple comparisons, with significance calculated relative to both PBS controls and non-targeting controls (*Ntc*).

## RESULTS

### Synthesis of conjugated siRNAs library

**Figure 1.**
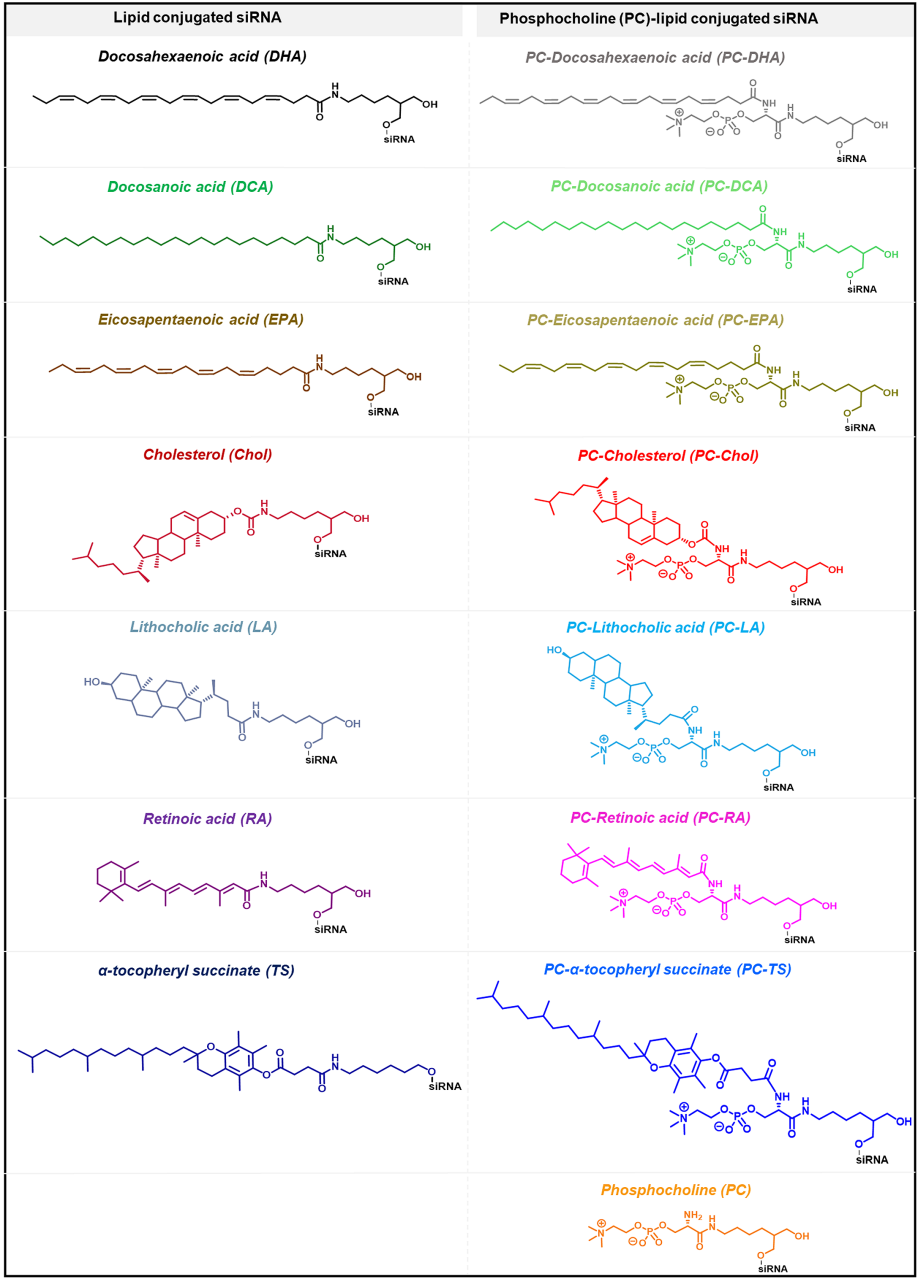
Library of studied lipid conjugated siRNAs. The desired conjugate is attached to the 3′ end of the sense strand of the siRNA though a C7 linker (left column) or a C7 linker functionalized with a phosphocholine group (PC) (right column).

To evaluate how lipid conjugates affect siRNA distribution *in vivo*, we synthesized a panel of siRNAs conjugated with naturally-occurring lipids, including: the unsaturated fatty acids docosahexaenoic acid (DHA, 22:6 n-3) and eicosapentaenoic acid (EPA, 20:5 n-3); the saturated fatty acid docosanoic acid (DCA, 22:0); the sterols cholesterol (Chol) and lithocholic acid (LA); and the vitamins retinoic acid (RA) and α-tocopheryl succinate (TS) (Figure 1). A significant fraction of circulating fatty acids are esterified with phosphatidylcholine or other head groups (37,38). Therefore, we synthesized each lipid conjugate with or without phosphocholine (PC) (33) to test whether this polar head group affects the distribution of lipid-conjugated siRNAs *in vivo*. As controls, we synthesized unconjugated siRNAs (Unconj.) and siRNAs with the PC head group only (no lipid). Each lipid conjugate was covalently attached to the 3′ end of the siRNA sense strand, which tolerates a range of covalent modifications (7,39,40). The siRNAs were fully chemically modified for maximal stability and minimal innate immune activation (see ‘Materials and Methods’ section).

The chemical compositions of lipids had a profound effect on the hydrophobicity of conjugated siRNAs, as determined by retention time in reversed-phase High Performance Liquid Chromatography (HPLC) (41). Lipid-conjugated siRNA retention times ranged from 1 to 12.2 min (Supplementary Figure S1). In general, compounds with a PC head group eluted 0.5 to 1.3 min earlier than their counterparts without a PC head group, suggesting that PC reduces hydrophobicity. Oligonucleotides without a lipid eluted the fastest, with PC-siRNA (1 min) eluting 4.7 min earlier than unconjugated siRNA (5.7 min).

To our knowledge, our panel of lipid-conjugated siRNAs is the largest collection to be synthesized, purified and characterized to date.

### Experimental design to evaluate the impact of lipid moiety on siRNA tissue accumulation and efficacy

**Figure 2.**
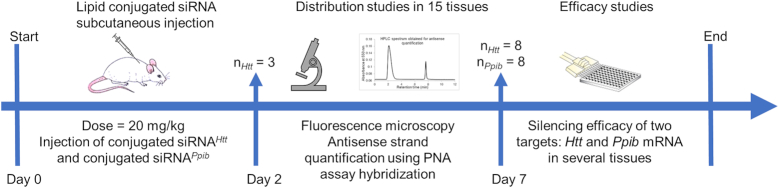
Experimental plan describing the study of one conjugated siRNA in mice. The experiment was performed for 15 conjugated and one unconjugated siRNA. In total, 321 mice were injected (included PBS-injected controls). For distribution studies, three mice per lipid-conjugated siRNA*^Htt^* were sacrificed. Fifteen tissues per mouse (liver, kidneys, lung, heart, thymus, spleen, pancreas, adrenal glands, intestine, fallopian tube, bladder, fat, muscle, skin at injection site and skin away from injection site) were collected and analyzed by fluorescence microscopy and High Performance Liquid Chromatography for antisense strand quantification using a PNA hybridization assay. For efficacy studies, eight mice injected with conjugated siRNA*^Htt^* and eight mice injected with conjugated siRNA*^Ppib^*per lipid conjugate were sacrificed. The expression of *Htt* mRNA or *Ppib* mRNA in tissues were measured using QuantiGene Assay.

Conjugated siRNA *in vitro* efficacy has minimal predictive value for *in vivo* distribution. To evaluate how the lipid moiety affects conjugated siRNA tissue distribution and efficacy, we designed an experiment (Figure 2) that uses three different assays. The experimental plan was performed for the 15 conjugated and one unconjugated siRNA, with each being injected subcutaneously into mice (*n* = 321 total mice, including controls) at a 20 mg/kg dose (Day 0).

For distribution studies, three mice per conjugated siRNA (*n* = 49 mice, with controls) were sacrificed at Day 2 post injection and 15 tissues per mouse—liver, kidney, adrenal gland, lung, heart, thymus, spleen, pancreas, intestine, fallopian tube, bladder, fat, muscle, injection site and skin—were collected (735 total samples). To evaluate the impact of lipid conjugates on spatial and cell-type specific siRNA distribution, we used fluorescence microscopy to visualize siRNA sense strands, which were labeled at the 5′-end with a Cy3 fluorophore. To quantify tissue antisense strand accumulation, we used a PNA hybridization assay (34,35). The PNA hybridization assay is not dependent on the presence of Cy3 on the sense strand; it directly quantifies the functionally-active non-labeled antisense strand and informs on the relative distribution of different compounds in different tissues. Thus, we could simultaneously evaluate spatial tissue distribution and quantify the antisense strands in the same samples. The PNA hybridization assay enables detection of a positive signal on a negative background, thus three mice per conjugate was sufficient to define tissue accumulation. We used overall tissue accumulation (all 15 tissues, adjusted by organ weight) to estimate injected-dose body retention versus initial kidney filtration.

The third assay used the remaining mice injected at Day 0 to examine the correlation between siRNA tissue accumulation and silencing. For this silencing efficacy study, eight mice per conjugated siRNA (*n* = 272 mice, with *Ntc*) were sacrificed at Day 7 post injection. Lipid-conjugated siRNAs targeted two different genes: *Huntingtin* (*Htt*) (31) or *Cyclophilin B* (*Ppib*) (42). We chose these targets because both (i) have validated siRNA sequences available, (ii) are widely expressed in the body, and (iii) are expressed at different levels (*Htt* low, and *Ppib* high). Per lipid conjugate, eight mice were injected with siRNA*^Htt^* and eight were injected with siRNA*^Ppib^* to detect signal reduction in the context of a positive background. Studies were powered to detect 15–20% modulation with 80% confidence. After sacrifice and tissue collection, we measured *Ppib, Htt*, and *Hprt* (hypoxanthine-guanine phosphoribosyl transferase, a housekeeping gene) mRNA levels using the Quantigene Assay.

### Cy3 dye has no major impact on tissue distribution profile of lipid-conjugated siRNA

**Figure 3.**
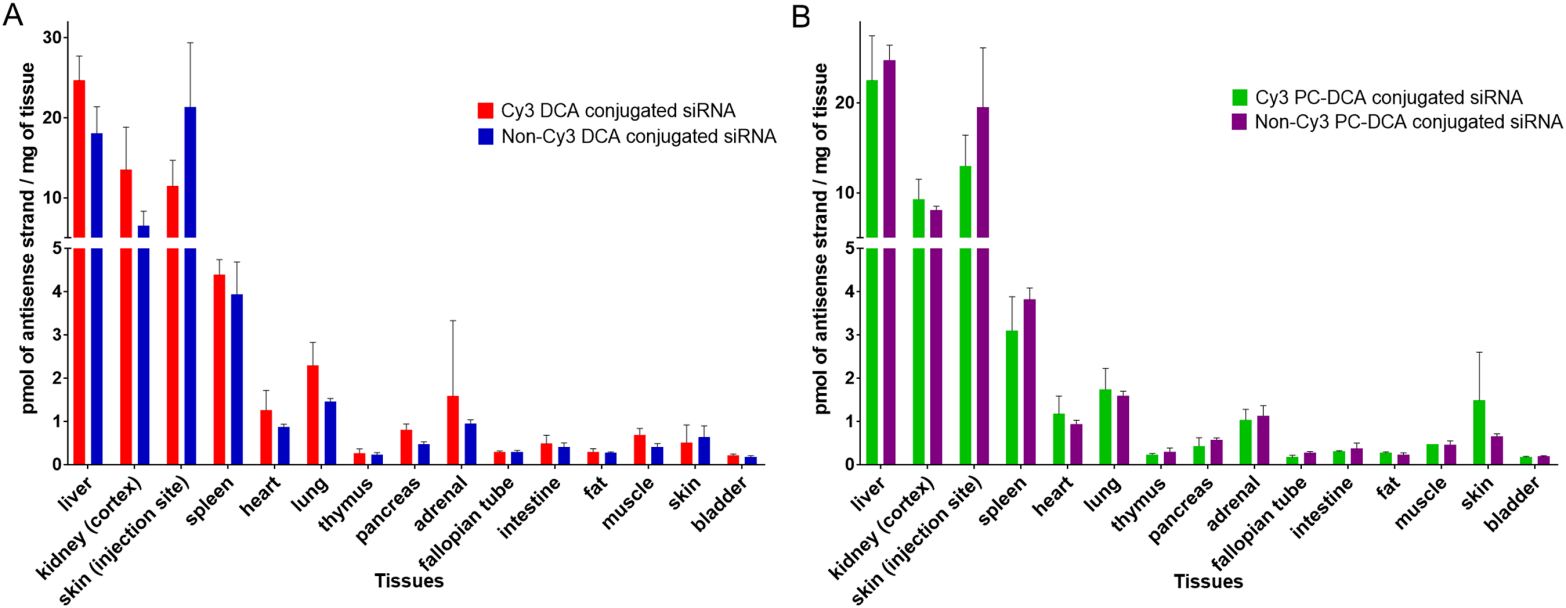
The presence of fluorophore Cy3 on the lipid-conjugated siRNA does not significantly affect siRNA distribution *in vivo*. Tissue accumulation levels of (**A**) Cy3-labeled and unlabeled DCA-conjugated siRNA (**B**) or Cy3-labeled and unlabeled PC-DCA conjugated siRNA were similar in 15 tissues. siRNA quantification was performed by PNA hybridization assay (average of three mice per conjugate ± SD) after a single subcutaneous injection of 20 mg/kg conjugated siRNA and tissue collection after 48 h.

The Cy3 fluorophore is slightly hydrophobic, and it has been previously stipulated that its presence may impact single stranded non-conjugated ASO delivery. In this study, we found that the relative contribution of Cy3 label to lipid-conjugated siRNA hydrophobicity was minimal. Indeed, while DCA or PC-DCA conjugation to the siRNA sense strand shifts reverse-phase HPLC retention times by 5 or 8 min compared to unconjugated sense strands, the relative impact of adding Cy3 dye is <0.5 min (Supplementary Figure S2). To evaluate if this slight change in hydrophobicity would affect siRNA tissue distribution profiles, we synthesized DCA and PC-DCA conjugated siRNAs with and without Cy3 label, injected them subcutaneously into mice (*n* = 3 per group), and evaluated tissue antisense strand accumulation (PNA hybridization assay, Figure 3). We observed no significant difference in primary and secondary tissue accumulations, indicating that the Cy3 label has no major impact on lipid-conjugated siRNA tissue distribution. Next, we evaluated the efficacy of Cy3 and non-Cy3 conjugated siRNAs (*n* = 6 per group) in nine tissues (liver, kidneys, skin, lung, heart, adrenal glands, fat, spleen and muscle, Supplementary Figure S3). We observed statistically significant silencing in all tissues except kidney, with no significant difference in the degree of silencing, except in the heart—Cy3 conjugated compounds were active (45% and 30% silencing, *P* < 0.0001) whereas non-Cy3 conjugated siRNAs were not.

These findings suggest that Cy3 has no impact on siRNA tissue distribution profile. However, minor changes in siRNA hydrophobicity due to Cy3 may impact productive silencing in a tissue-specific manner. This should be considered when interpreting the reported data.

### The chemical nature of the lipid conjugate defines the liver/kidney distribution profile

Oligonucleotides preferentially distribute to two tissues: liver and kidney. For ASOs, size and phosphorothioate (PS) modifications define the relative liver/kidney distribution via serum protein binding mechanisms. Large fully-PS ASOs preferentially distribute to liver due to tight serum protein binding. Decreasing ASO size/PS content reduces serum binding affinity, shifting accumulation to kidney proximal epithelia, which retain the non serum-binding ASO fraction during clearance (11,43,44).

**Figure 4.**
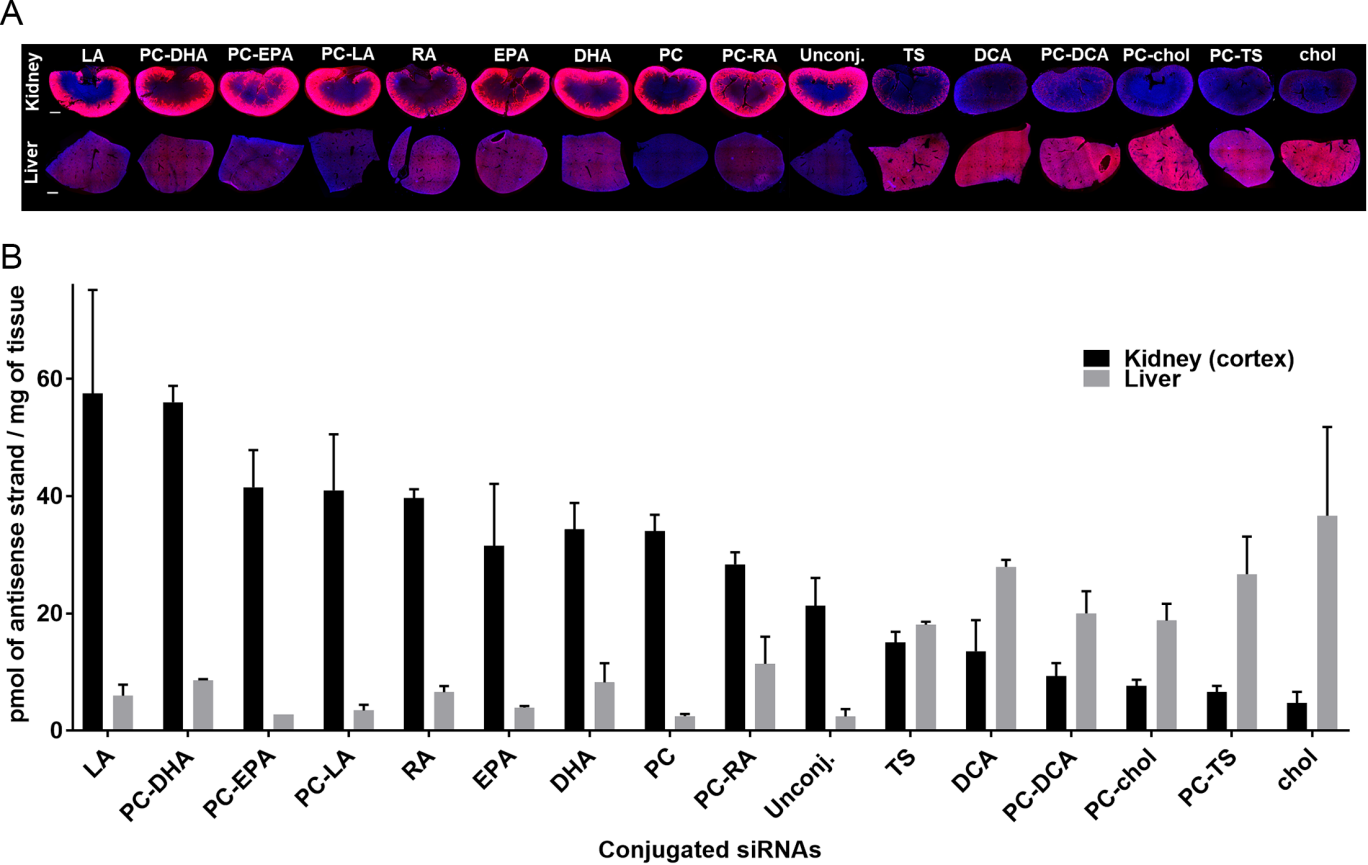
The conjugate defines the ratio of liver to kidney distribution. (**A**) Representative fluorescence images of kidney and liver sections from mice injected subcutaneously with 20 mg/kg Cy3-labeled lipid-conjugated siRNAs. Nuclei stained with DAPI. Three mice per conjugate were injected and tissue collected 48 h after injection. Images taken at 5 × magnification and collected at the same laser intensity and acquisition time. Scale, 1 mm. (**B**) siRNA quantification in kidney (cortex) and liver was performed by PNA hybridization assay (average of three mice per conjugate ± SD).

For conjugated siRNAs, the chemical nature of the lipid conjugate, rather than size and PS content, had a profound impact on the relative liver-to-kidney distribution. Figure 4 shows representative fluorescent images (Figure 4A) and antisense strand quantification (Figure 4B) of liver and kidney. Unconjugated siRNAs distribute mostly to kidneys; our siRNA configuration contains a 13-PS backbone (within 35 nt) that supports retention during filtration. Conjugated siRNAs exhibited enhanced kidney and liver accumulation compared to unconjugated siRNA. The highest kidney levels (57 pmol of antisense strand/mg of tissue) were achieved by LA-siRNAs (three times higher than unconjugated siRNA). Cholesterol-siRNAs exhibited the highest liver accumulation (36 pmol of antisense strand/mg of tissue). Generally, compounds with higher kidney accumulation had lower liver accumulation, and vice versa. Like ASOs, this trend is defined by serum binding profile: compounds with higher delivery to kidney preferentially bind to high-density lipoproteins (HDL), and compounds with high liver delivery bind to low-density proteins (LDL) (Osborn*et al.*, NAR, in this issue) (22).

There is no direct correlation between degree of hydrophobicity and liver accumulation. The most hydrophobic compound (TS-siRNA, Supplementary Figure S1) showed equal liver and kidney accumulation, indicating that other factors contribute to the observed profile. Overall, these data demonstrate that lipid conjugation can be used to alter the liver-to-kidney distribution profile, and enhance kidney delivery.

### The chemical nature of the conjugates affects cell-type distribution in kidney and liver

**Figure 5.**
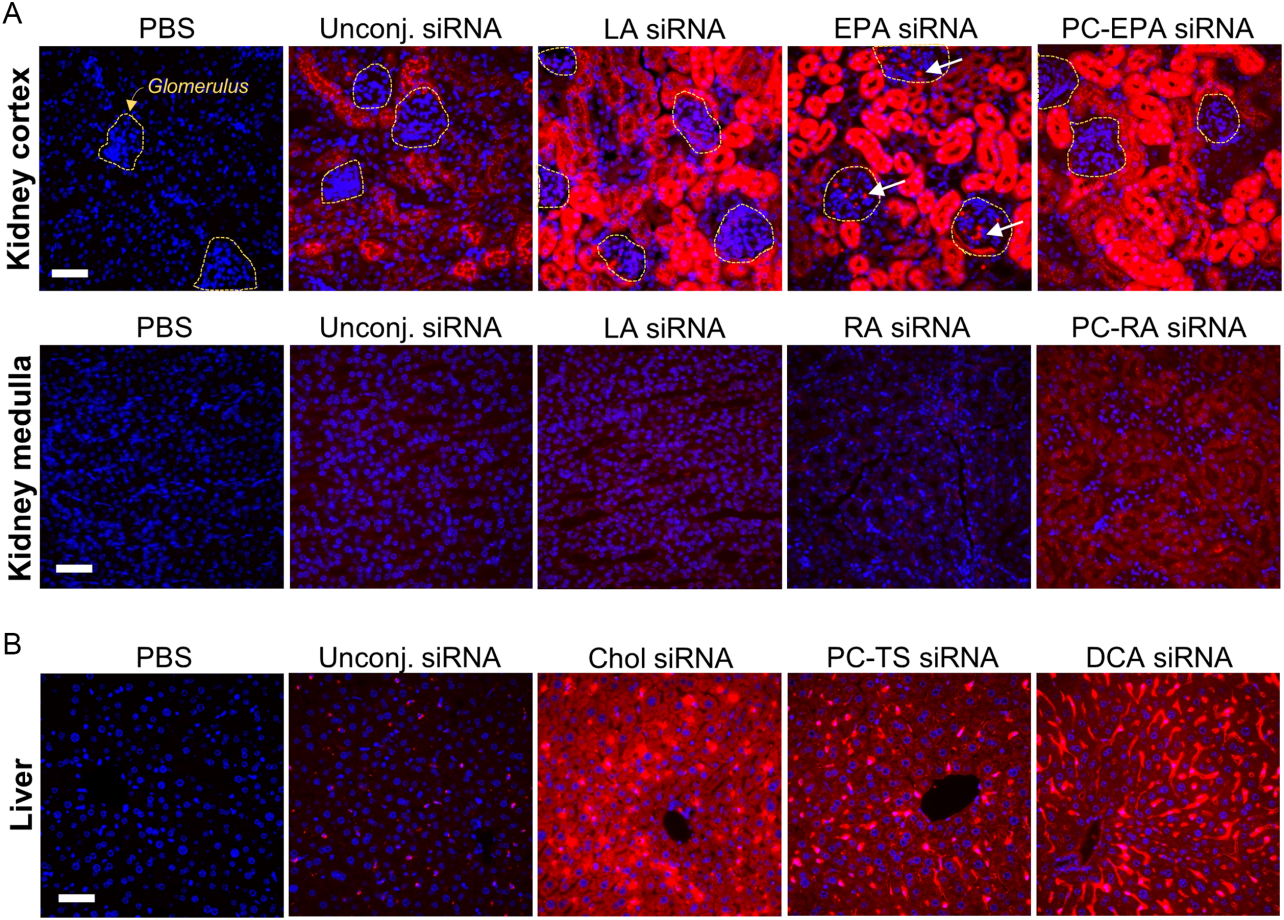
The conjugate impacts the cell-type distribution in kidney and liver. Representative fluorescence images of (**A**) kidney cortex and kidney medulla or (**B**) liver sections from mice injected subcutaneously with 20 mg/kg Cy3-labeled lipid-conjugated siRNAs. Nuclei stained with DAPI. Three mice per conjugate were injected and tissue collected 48 h after injection. Images taken at 40 × magnification and collected at the same laser intensity and acquisition time. Scale, 50 μm.

In the kidney, all conjugates preferentially delivered to kidney proximal epithelia, consistent with its role in filtration. Figure 5A shows representative examples of siRNA delivery to the medulla and glomerulus, an easily distinguishable histological structure responsible for large protein re-absorption and involved in many human diseases. The degree of distribution to glomerulus and medulla varied significantly among conjugated-siRNAs, and could not be explained by overall organ accumulation or compound hydrophobicity. LA-siRNAs achieved the highest accumulation in the kidney but were not distributed beyond epithelial cells. Unconjugated siRNAs, which differ in hydrophobicity from LA-siRNA, showed a similar distribution pattern: no accumulation in the glomerulus or medulla. By contrast, EPA- (and DHA-) siRNAs, which have relatively similar hydrophobicity to LA-siRNAs, showed very clear delivery to the glomerulus, with diffuse staining and distinct foci being detected. Interestingly, PC-EPA and PC-DHA siRNAs did not accumulate in the glomerulus. Moreover, PC-RA siRNAs showed significant accumulation in the medulla, whereas RA-siRNAs exhibited low distribution. These findings suggest that individual conjugate properties, including the PC moiety, impact distribution within the kidney.

A similar phenomenon was observed in the liver (Figure 5B). Three conjugated siRNAs (cholesterol, PC-TS and DCA siRNAs) with similar overall delivery to the liver exhibited distinct distributions within the organ. Cholesterol-siRNAs were detected in Kupffer cells (liver residual macrophages) and hepatocytes. PC-TS siRNAs had a similar distribution pattern, but with lower accumulation in hepatocytes and some detectable endothelial retention. The majority of DCA siRNAs accumulated in endothelial cells and Kupffer cells, with significantly reduced hepatocyte distribution. Since these compounds have similar hydrophobicity (Supplementary Figure S1) and serum binding profiles (Osborn *et al.*, NAR, in this issue) (22), the difference in delivery within the liver is likely due to individual conjugate properties. For unconjugated siRNA, which had minimal liver delivery, distribution was almost exclusively limited to Kupffer cells.

### The chemical nature of the lipid conjugate has a profound impact on the degree of overall siRNA retention

**Figure 6.**
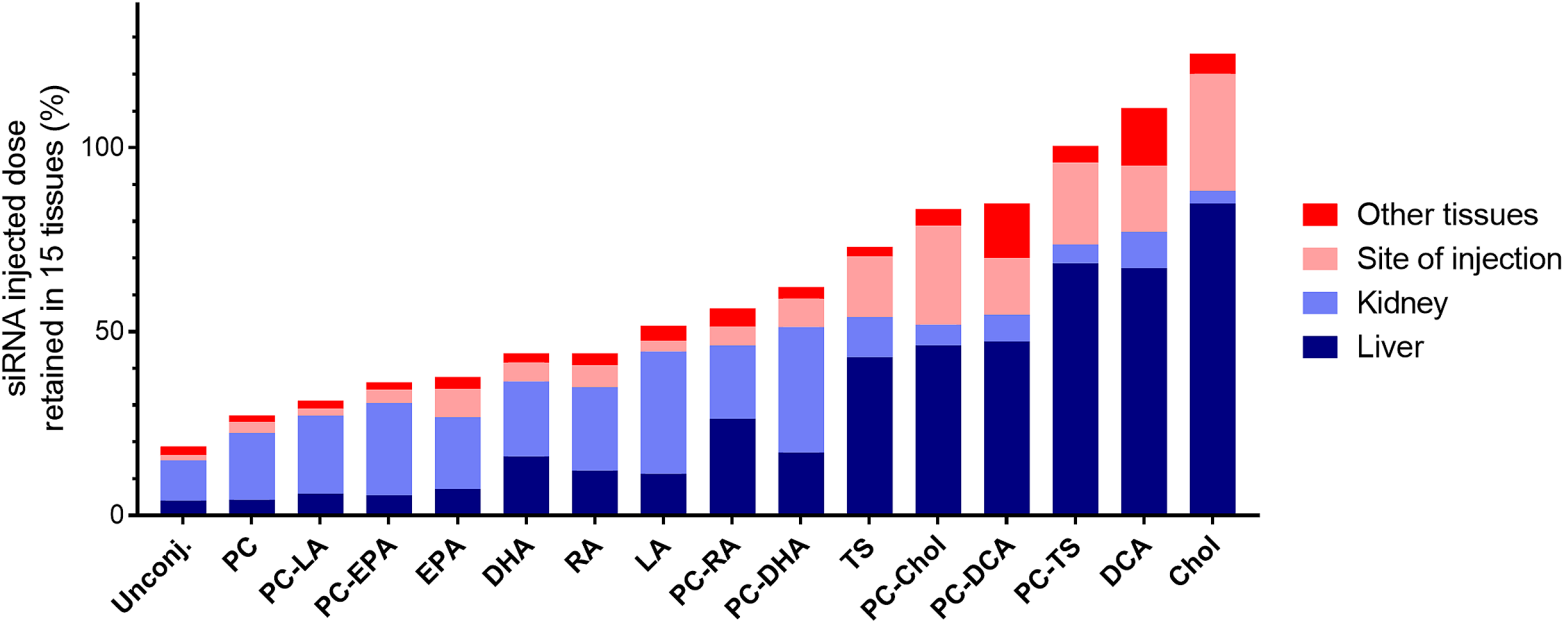
The nature of the lipid conjugate has a profound impact on siRNA tissue retention. Bar graph showing percent of the injected dose cumulatively retained in mice across all 15 tissues 48 h after a single subcutaneous injection of 20 mg/kg conjugated siRNA (average of *n* = 3 mice). Stacked bars indicate the percent of the retained dose in liver, kidney, site of injection and others tissues. More than 80% of unconjugated siRNAs are cleared while highly hydrophobic compounds are retained quantitatively.

We measured antisense strand accumulation in fifteen tissues that comprise most of the mouse body, allowing us to estimate the fraction of injected siRNA dose retained after 48 h (Figure 6). The relative mass of each tissue has been experimentally defined, or approximated based on published mouse organ weights (45–47).

More than 85% of unconjugated siRNA was cleared from the body within minutes after injection, and the majority of the retained siRNA accumulated in kidney proximal epithelial cells. The addition of a lipid conjugate improved siRNA overall body retention; highly hydrophobic conjugates like DCA and cholesterol allowed quantitative siRNA tissue distribution, indicating relatively low contribution of kidney filtration to serum clearance.

Generally, more hydrophobic compounds (PC-TS, DCA and cholesterol siRNAs) were retained to a higher extent than less hydrophobic compounds (PC, PC-LA and PC-EPA siRNAs), with some exceptions. For example, the presence of the PC group, which generally descreases hydrophobicity, had a variable impact on retention: PC-DHA, PC-TS and PC-RA siRNAs showed higher retention (∼20%) than their non-PC head group counterparts. This indicates that hydrophobicity is not the single determinant for overall tissue retention.

Consistent with this notion, compounds with significantly different hydrophobicity profiles (PC-DHA and TS siRNAs, Supplementary Figure S1) showed similar (∼65%) body retention. Thus, the nature of the conjugate has an impact on siRNA retention on top of overall serum binding-defined clearance (Osborn *et al.*, NAR, in this issue) (22).

### DCA and PC-DCA conjugates improve extra-hepatic delivery of siRNA compared to cholesterol

To date, cholesterol is the only conjugate that has been explored for extra-hepatic delivery of siRNAs. To evaluate the impact of different conjugates on siRNA extra-hepatic delivery, we analyzed the distribution of lipid-conjugated siRNAs 48 h after injection in adrenal glands, lung, heart, thymus, spleen, pancreas, intestine, fallopian tube, bladder, fat, muscle and skin (at and far from the injection site). Supplementary Figures S4–18 show fluorescence microscopy images (5 × and 40 × magnification) of each tissue for each lipid-siRNA, and antisense strand concentration in each tissue. The quantitative data for all 16 compounds in all 15 tissues are summarized in Supplementary Figure S19.

**Figure 7.**
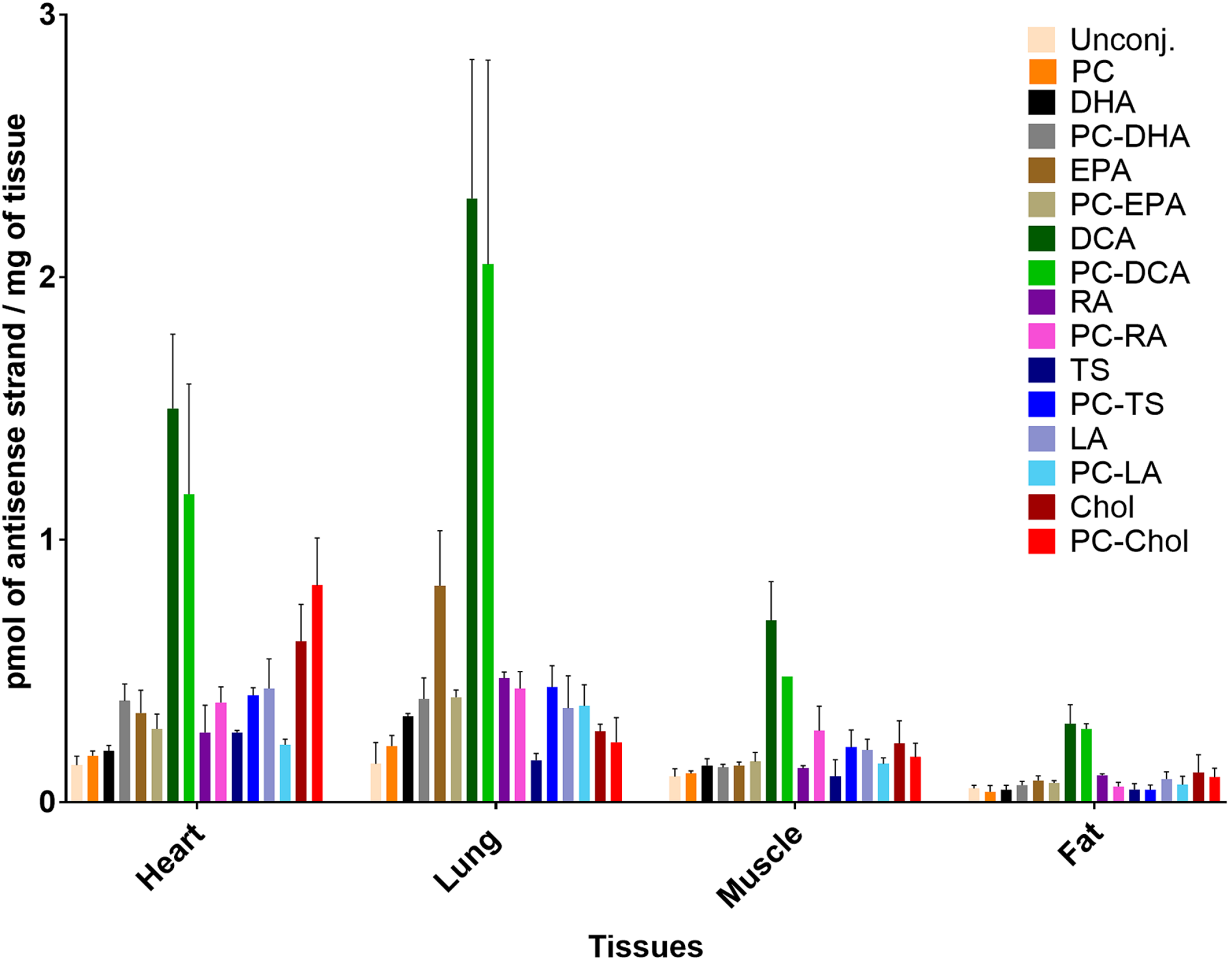
Conjugated siRNAs accumulate into extra-hepatic tissues, accumulation defines by the structure of the conjugate. Bar graph showing the quantity of the antisense strand of conjugated siRNAs present in heart, lung, muscle and fat 48 h after a single subcutaneous injection with 20 mg/kg (*n* = 3 mice per conjugate ± SD). siRNA quantification was measured by PNA hybridization assay. DCA and PC-DCA conjugates improve extra-hepatic siRNA accumulation compared to cholesterol.

**Figure 8.**
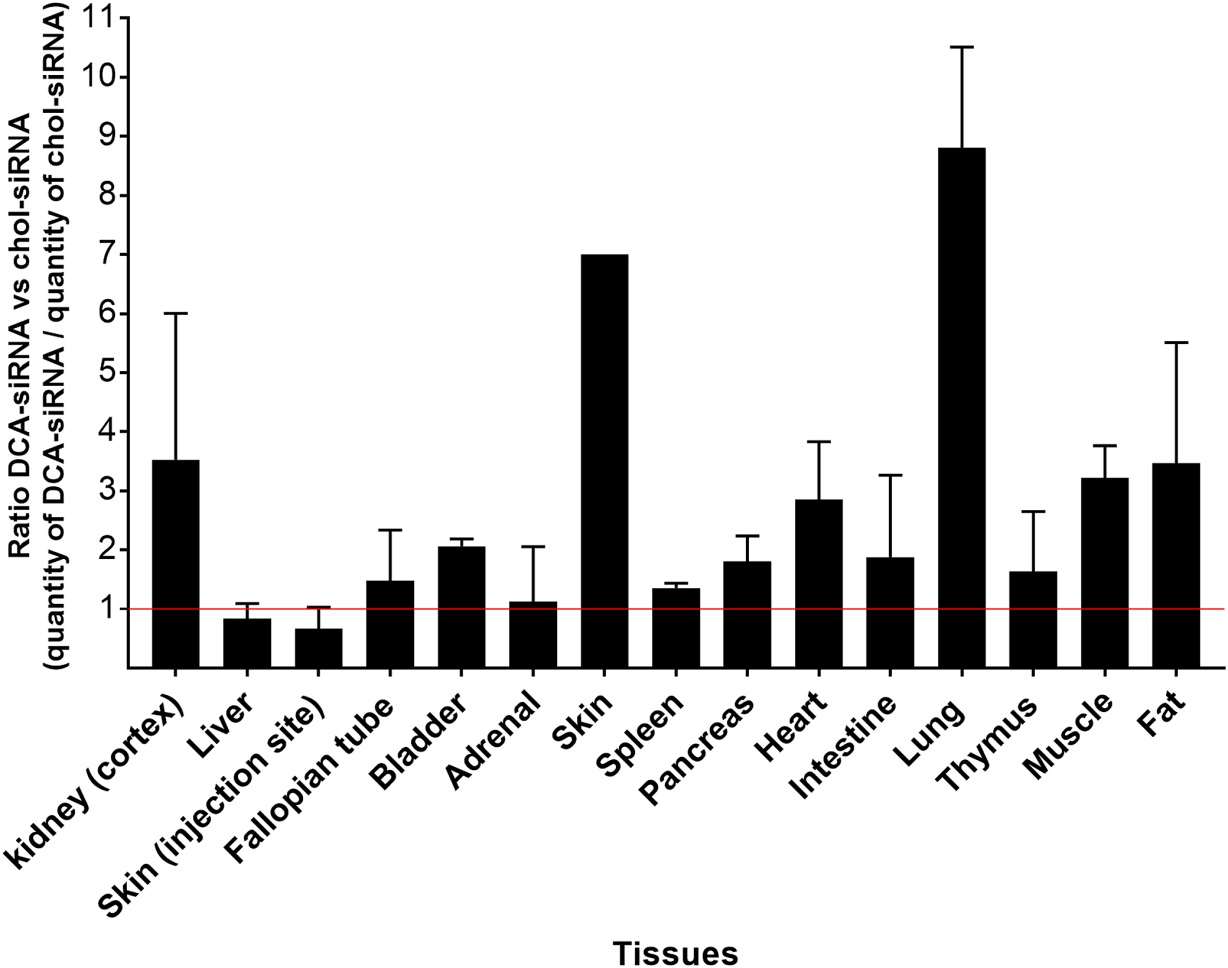
Relative improvement of DCA conjugated siRNA extra-hepatic tissue accumulation compared to cholesterol conjugated siRNA. Bar graph showing the ratio between the quantity of the DCA-siRNA antisense strands and cholesterol-siRNA antisense strands present in 15 tissues 48 h after a single subcutaneous injection with 20 mg/kg (*n* = 3 mice per conjugate ± SD). siRNA quantification was measured by PNA hybridization assay. DCA accumulation was 3- to 4-fold higher in heart, muscle and fat, ∼6-fold higher in skin (systemic) and 9-fold higher in lung.

Figure 7 shows quantification of antisense strand accumulation in heart, lung, muscle and fat. It is immediately clear that DCA and PC-DCA siRNAs distributed to extra-hepatic tissues significantly more than the others. In lung, EPA-siRNAs also showed significant accumulation. Figure 8 shows relative enhancement of tissue antisense strand accumulation of DCA-conjugated compounds compared to cholesterol-conjugated compounds. DCA accumulation was nine-fold higher in lung, and 3-fold higher in heart, fat and muscle compared to cholesterol. This finding suggests that modulating lipid conjugate configuration can alter compound tissue distribution, and potentially enhance extra-hepatic delivery.

### Lipid-conjugated siRNAs enable functional gene silencing in several extra-hepatic tissues, including heart, lung, muscle, fat and adrenal glands

To determine if lipid-conjugated siRNAs accumulate to levels sufficient for productive silencing, we injected mice with lipid-conjugated siRNAs targeting *Htt* (31) or *Ppib* (42). A week later, we measured *Ppib, Htt* and *Hprt* mRNA levels. At the dose tested (20 mg/kg), all conjugated siRNAs were well tolerated: we observed no adverse events or changes in blood chemistry (Supplementary Figure S37). For tissues that retained, for the majority of the compounds, more than 5 ng of conjugated siRNA per mg of tissue (liver, kidney, skin injection site, spleen, adrenal glands, heart, lung and skin), we measured the activity of all 15 conjugated siRNAs and the unconjugated siRNA. For the remaining organs (intestine, bladder, fat, muscle, pancreas, thymus and fallopian tube), we measured the activity of the conjugated siRNA with the highest accumulation level for each tissue. The silencing efficiency of each compound in each tissue is shown in Supplementary Figures S20–34, and data are summarized in a heat map in Supplementary Figure S35. *Ntc* showed no significant reduction in target gene expression, indicating that the observed silencing is due to sequence-specific effects, not the general chemical scaffold. Unconjugated siRNA induced silencing only in the kidney, confirming that statistically significant silencing depends on conjugate-mediated delivery. In general, the ability of a conjugated siRNA to silence in a tissue was similar for both targets.

**Figure 9.**
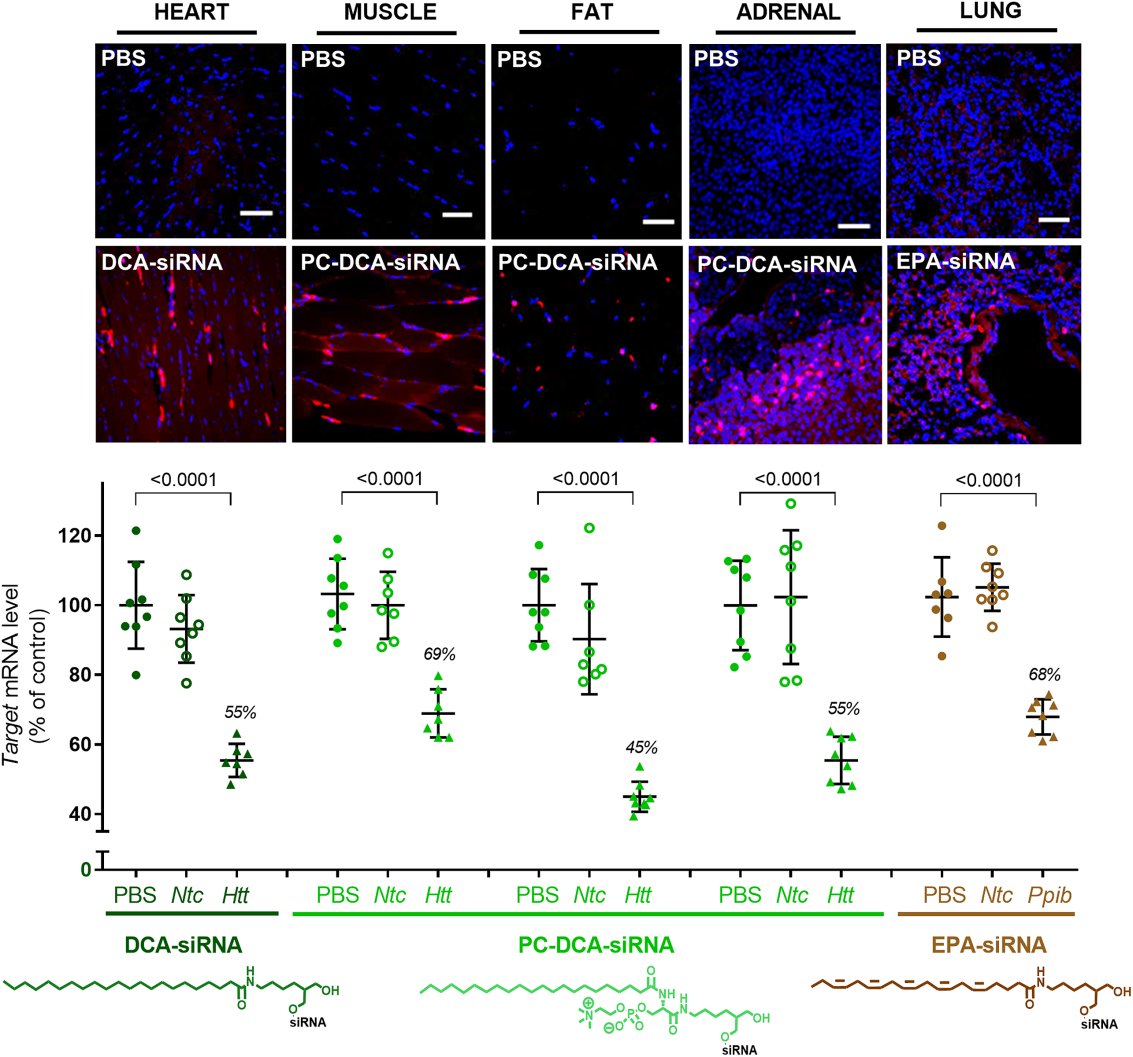
Accumulation of conjugated siRNA in several extra-hepatic tissues such as heart, muscle, fat, adrenal glands and lung are sufficient to induce mRNA silencing. Representative fluorescence images of heart, muscle, fat, adrenal glands and lung sections from mice injected subcutaneously with 20 mg/kg Cy3-labeled lipid-conjugated siRNAs or PBS. Nuclei stained with DAPI. Three mice per conjugate were injected and tissue collected after 48 h. Images taken at 40 × magnification and collected at the same laser intensity and acquisition time. Scale, 50 μm. For the measurement of *Huntingtin* (*Htt*) or *Cyclophilin* B (*Ppib*) mRNA levels, mice were injected subcutaneously with 20 mg/kg of conjugated siRNA (*n* = 16 per conjugate: 8 with siRNA targeting *Htt* and 8 with siRNA targeting *Ppib*, as well as non-targeting controls). The tissues were collected after 1 week and mRNA levels were measured using QuantiGene^®^ (Affymetrix), normalized to a housekeeping gene, *Hprt* (Hypoxanthine-guanine phosphoribosyl transferase) and presented as percent of PBS control (mean ± SD).

Figure 9 shows representative data for delivery and functional silencing in key tissues for the best-delivered conjugated siRNA per tissue: DCA siRNA for heart, PC-DCA siRNA for muscle, fat, and adrenal gland, and EPA siRNA for lung. These compounds induce 31–55% silencing and all observed effects were statistically significant (*P* < 0.001, One-way ANOVA with Bonferroni correction) relative to PBS and *Ntc*. The exact degree of silencing differed slightly between the two tested targets, likely due to variable cell-type specific expression and degree of nuclear mRNA retention.

These data demonstrate the feasibility of functional extra-hepatic delivery of siRNAs, and identify particular conjugates that enable the highest reported siRNA accumulation in extra-hepatic tissues to date. Further sequence optimization combined with repetitive dosing will be necessary to achieve full silencing.

**Figure 10.**
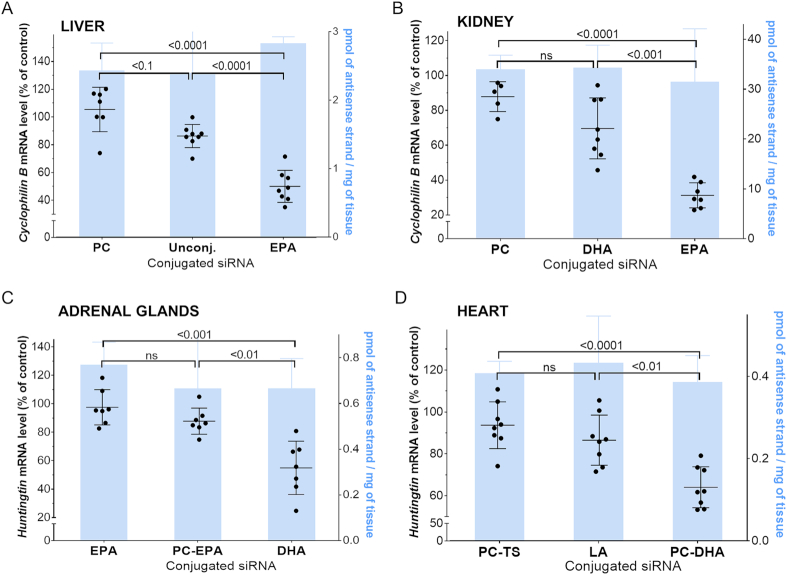
The efficiency of target mRNA silencing does not necessarily correlate with siRNA accumulation level. Graphs showing target mRNA silencing (relative to PBS) for different conjugates with similar tissue accumulation (right *Y*-axis, bar). Mice were injected subcutaneously with 20 mg/kg lipid-conjugated siRNAs (*n* = 3 ± SD, 48 h for antisense strand quantification; *n* = 8 ± SD, 1 week for target gene silencing QuantiGene). While several conjugates show similar tissue accumulation, the functional silencing is conjugate-specific.

We observed that higher oligonucleotide tissue accumulation tended to translate into silencing at high degrees of accumulation (Supplementary Figure S36). However, this relationship was not absolute, indicating that oligonucleotide tissue accumulation levels do not directly predict functionality. Figure 10 shows examples of several conjugated siRNAs that accumulate in tissues to the same extent but produce functionally different results. Unconjugated, PC-conjugated, and EPA-conjugated siRNAs showed similar liver accumulation, but EPA-siRNAs induced ∼50% silencing while PC and unconjugated siRNAs were not functional. The same phenomena can be observed in kidneys, adrenal glands and heart. This was specifically striking for kidneys, where many compounds accumulate significantly (>300 ng/mg), yet do not demonstrate functional silencing. For example, PC-LA siRNAs (536 ng/mg; Supplementary Figure S5) did not support silencing, while PC-EPA siRNAs, which accumulate in kidney to the same degree, induced 30% silencing (Supplementary Figure S21). This suggests that the chemical nature of the lipid conjugate influences internalization mechanisms and/or degree of endosomal escape, which profoundly affects functional silencing.

## DISCUSSION

GalNAc-conjugated siRNAs have dominated the recent development of therapeutic oligonucleotides for liver indications. The GalNAc moiety drives specific delivery to and activity in hepatocytes (17–19). Here we show that lipid conjugates support much broader delivery of siRNAs and enable functional silencing in many tissues, including liver, kidney, lung, heart, muscle, spleen, fat and adrenal glands. To date, only one hydrophobic conjugate—cholesterol—has been explored for extra-hepatic delivery, with demonstrated functional silencing in placenta (27) and muscle (26). Our study found that some lipid conjugates, particularly DCA and PC-DCA, support higher siRNA accumulation than cholesterol in extra-hepatic tissues, including muscle, heart, fat and lung, without causing overt toxicity. This suggests that lipid conjugate engineering can enhance extra-hepatic delivery, and expand the therapeutic potential of siRNAs beyond the liver.

To broaden delivery of siRNA to tissues, they must be retained in the body. Yet, the majority of unconjugated siRNAs are expected to be cleared on first passage through the kidney because their size, ∼13 kDa, is within the limit of active filtration (∼40 kDa). Indeed, ∼85% of unconjugated siRNA was rapidly cleared from the body. The retained unconjugated siRNA mainly accumulated in kidney proximal epithelia. This retention is due to the PS modifications in our siRNA design (13 PS bonds within a 35-nt scaffold), and is similar to the retention mechanism of short single-stranded PS-ASOs (11,43,44). Lipid conjugates significantly increased overall siRNA tissue retention, with a trend for highly hydrophobic siRNAs (cholesterol, DCA, PC-TS conjugated) being retained to a higher degree than less hydrophobic siRNAs (EPA- and DHA-conjugated), but this relationship was not absolute.

The observed increase in overall tissue retention is likely due to reduced clearance rates (34), which increase tissue exposure. Similar to small molecules, lipid conjugates differentially bind to serum proteins, which affects siRNA distribution (liver-to-kidney distribution ratio) (23) (Osborn *et al.*, NAR in this issue) (22). However, serum binding is not the only factor defining distribution. Both cholesterol and DCA-siRNA preferentially interact with serum LDL (Osborn *et al.*, NAR, in this issue) (22). Yet their distribution *in vivo* was very different. DCA-siRNA showed higher accumulation in extra-hepatic tissues, such as fat, lung, muscle and heart compared to cholesterol-siRNA. Moreover, while their overall liver delivery was similar, cholesterol-siRNA accumulated in hepatocytes and Kupffer cells and DCA siRNA showed a clear preference for endothelial cells. Thus, serum binding profile may define organ exposure, but the nature of the conjugate impacts spatial intra-organ distribution and relative rates of internalization by different cell types.

Similarly, in the kidney, only EPA- and DHA-siRNA delivered to the glomerulus. However, functionalization of EPA- and DHA-siRNA with a PC head group eliminated this accumulation. The PC moiety also impacted medulla distribution: PC-RA, but not RA, enabled significant siRNA medulla delivery. These data support the notion that chemical engineering of lipid-conjugated siRNA can be used to alter intra-organ distribution. Future studies should evaluate whether cell-specific kidney delivery is functionally active. This will require the development of compounds targeting genes expressed exclusively in one cell type.

The imperfect correlation between siRNA tissue accumulation and productive silencing was most pronounced in the kidney, where many conjugated siRNAs accumulated significantly (>30 μg/g) but were not efficacious. The nature of the conjugate may define productive silencing by impacting cellular internalization and/or endosomal escape. In general, relatively high siRNA accumulation was necessary to produce efficacy in the kidney, indicating that the primary mechanism of kidney retention is tubular filtration. When retention occurs as a byproduct of filtration, the majority of internalized siRNA is trapped non-productively. By contrast, cellular internalization in muscle and fat results from active endocytosis, and relatively low accumulation (4–7 μg/g) is sufficient for productive silencing. This observation is consistent with a recent report that found required siRNA muscle accumulation levels for productive silencing are much lower than that for liver (Vasant Jadhav, OTS meeting, 2017).

Similar to the years of chemical evolution that have substantially improved the efficacy and safety of another siRNA delivery approach: lipid-based nanoparticles (LNP) (48,49), we expect that further lipid engineering will enhance conjugated siRNA extra-hepatic efficacy and delivery. One advantage of direct siRNA conjugation over LNP is the elimination of the ‘particle size’ effect, which largely defines initial LNP clearance (50). Indeed, differences in fenestrated capillary between tissues lead to the extravasation of particles with different diameters: ∼2 nm in heart, muscle, lung, and skin; ∼30 nm in kidneys; and ∼150 nm in liver and spleen. Thus, advancements in chemical engineering of lipid-conjugated siRNAs will open up a wider array of tissues for therapeutic intervention.

The potential for widespread siRNA delivery also comes with limitations. Lipid-conjugated siRNAs are not delivered to specific tissues, and most of the injected dose will be delivered to primary clearance tissues, including liver, kidney and spleen. Nevertheless, lipid-siRNAs could be used to silence: (i) targets that are exclusively expressed in the disease tissue, or (ii) targets that are widely expressed, but loss of activity is tolerated in healthy tissues. For example, fully chemically stabilized cholesterol-conjugated siRNAs have been used to silence soluble FTL1, which is overexpressed in placentas of pregnant subjects with preeclampsia (27). Though cholesterol-siRNA targeting sFLT1 is efficiently delivered to placenta (∼8% of injected dose), it also accumulates in the liver and kidney, where sFLT1 silencing is believed to be tolerated and irrelevant for disease progression. DUX4 in muscular dystrophy is a similar type of target (51). The use of lipid conjugates offers potential gene modulation in many tissues, as long as target and clinical indication are carefully considered and matched to the pharmacologic and safety profile of the lipid-siRNA conjugate.

This study reports the first example of chemically engineered lipid-conjugated siRNAs for systemic delivery and shows that medicinal chemistry efforts to better improve extra-hepatic tissue accumulation and efficacy is essential to advance the RNA therapeutic field. Future expansion of the chemical space of lipid moieties will establish a path toward enhancing siRNA delivery and potency.

## Supplementary Material

Supplementary Data
