# Supplementary material for: Diverse lipid conjugates for functional extra-hepatic siRNA delivery in vivo
Source: Nucleic Acids Res. 2018 Dec 14;47(3):1082–96. doi: 10.1093/nar/gky1239 (PMC6379722; doi:10.1093/nar/gky1239)
Supplement: Supplementary Data [file gky1239_supplemental_files.pdf]

Supplementary Information

Diverse lipid conjugates for functional extra-hepatic siRNA delivery *in vivo*

Annabelle Biscans,[1,2] Andrew Coles,[1,2] Reka Haraszti,[1,2] Dimas Echeverria,[1,2] Matthew Hassler, [1,2] Maire Osborn,[1,2] and Anastasia Khvorova[1,2*]

[1] RNA Therapeutics Institute, University of Massachusetts Medical School, Worcester, 01604, MA, USA

[2] Program in Molecular Medicine, University of Massachusetts Medical School, Worcester, 01604, MA, USA

*Correspondence should be addressed to A.K. (Anastasia.khvorova@umassmed.edu)

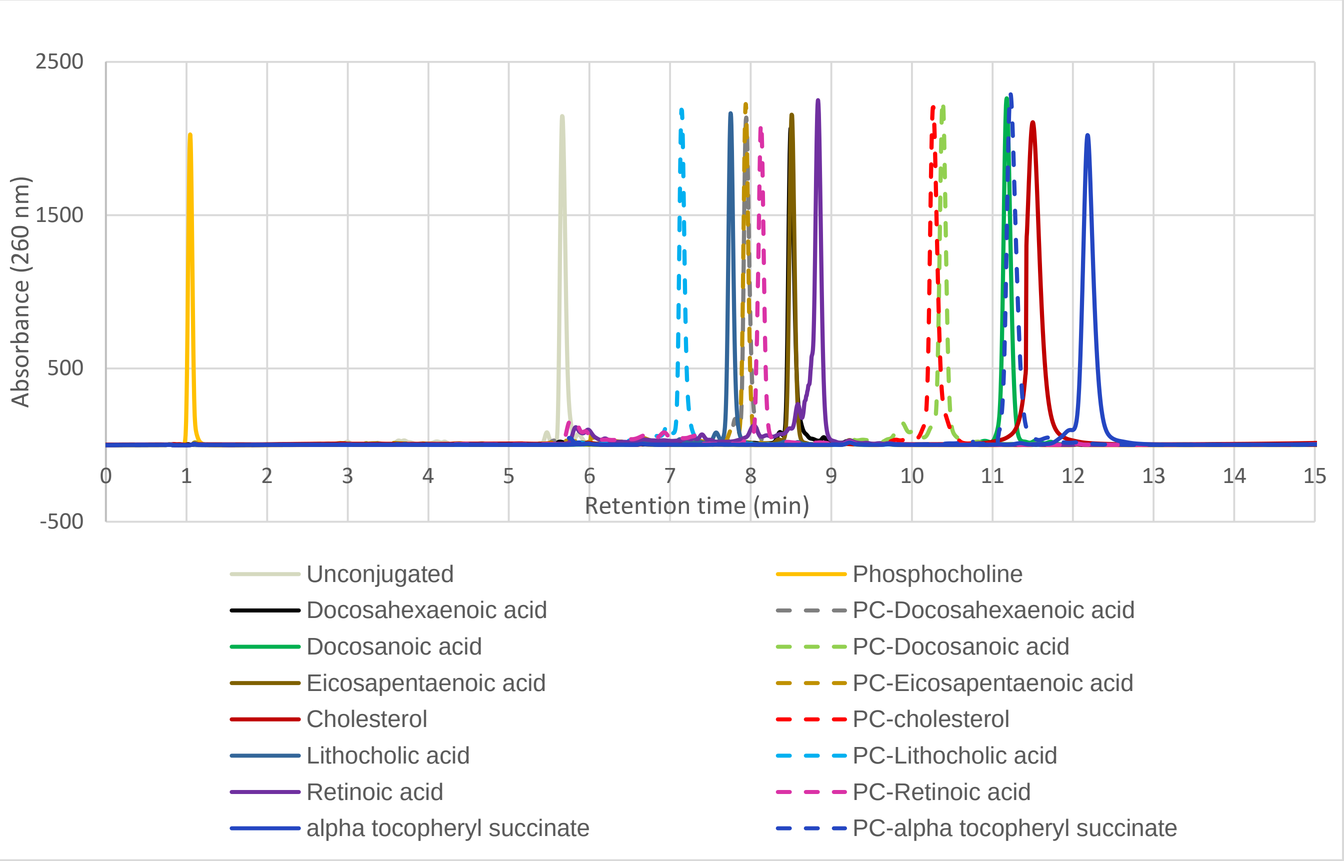

Supplementary Figure 1: The chemical composition of the conjugates has a significant impact on siRNA hydrophobicity. High Performance Liquid Chromatography spectra of conjugated Cy3-siRNA*^{Htt}* sense strands showing variation in retention time (hydrophobicity) according the conjugate (C18, Buffer A = 0.1 M Triethylammonium acetate in water, Buffer B = Acetonitrile, Gradient = 0-100 % in B in 15 min, Temperature = 60C, Flow = 1 mL/min)

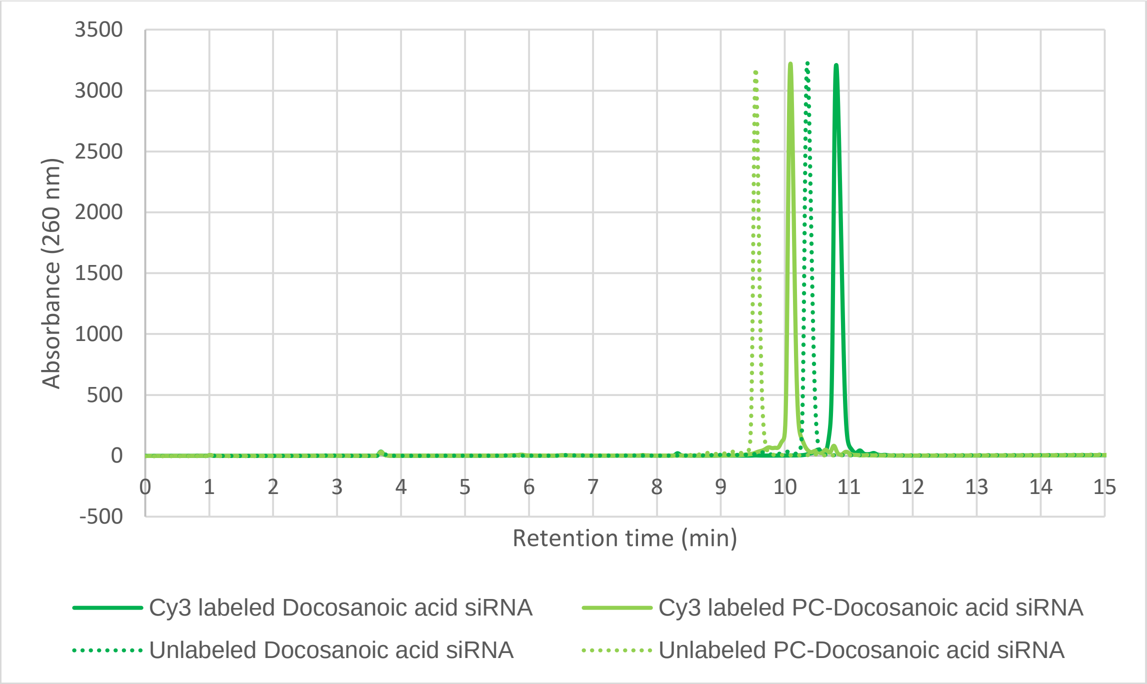

Supplementary Figure 2: The presence of Cy3 has a minimal impact on siRNA hydrophobicity. High Performance Liquid Chromatography spectra of unlabeled DCA or PC-DCA conjugated siRNA^{Htt} sense strands or Cy3-labeled or PC-DCA conjugated siRNA^{Htt} sense strands (C18, Buffer A = 0.1 M Triethylammonium acetate in water, Buffer B = Acetonitrile, Gradient = 0-100 % in B in 15 min, Temperature = 60C, Flow = 1 mL/min)

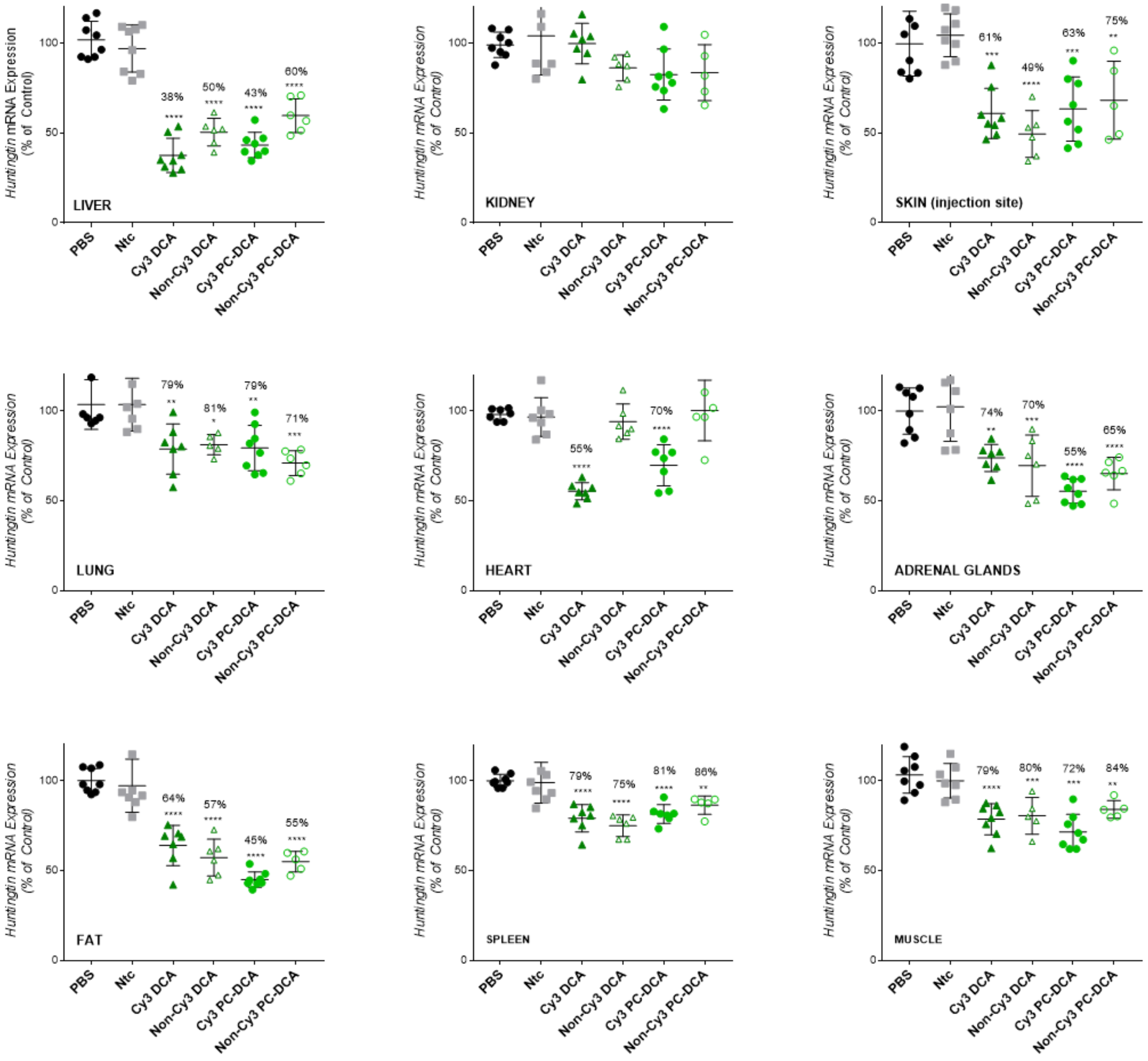

Supplementary Figure 3: Efficacy of Cy3 and non-Cy3 DCA or PC-DCA siRNA^{Htt} in liver, kidneys, skin (injection site), lung, hear, adrenal glands, fat, spleen and muscle. Subcutaneous injection (FVB/N mice); 20 mg/kg; collection of tissues one week after injection; n = 6 per conjugate. Huntingtin (*Htt*) mRNA levels were measured using QuantiGene® (Affymetrix), normalized to a housekeeping gene, *Hprt* (Hypoxanthine-guanine phosphoribosyl transferase), and presented as percent of PBS (Phosphate buffered saline) control (mean ± SD). Data analysis: Outliners define with Grubb's method (alpha = 0.1%); Multiple comparisons = One-way ANOVA, Bonferroni test (****P<0.0001, ***P<0.001, **P<0.01, *P<0.1).

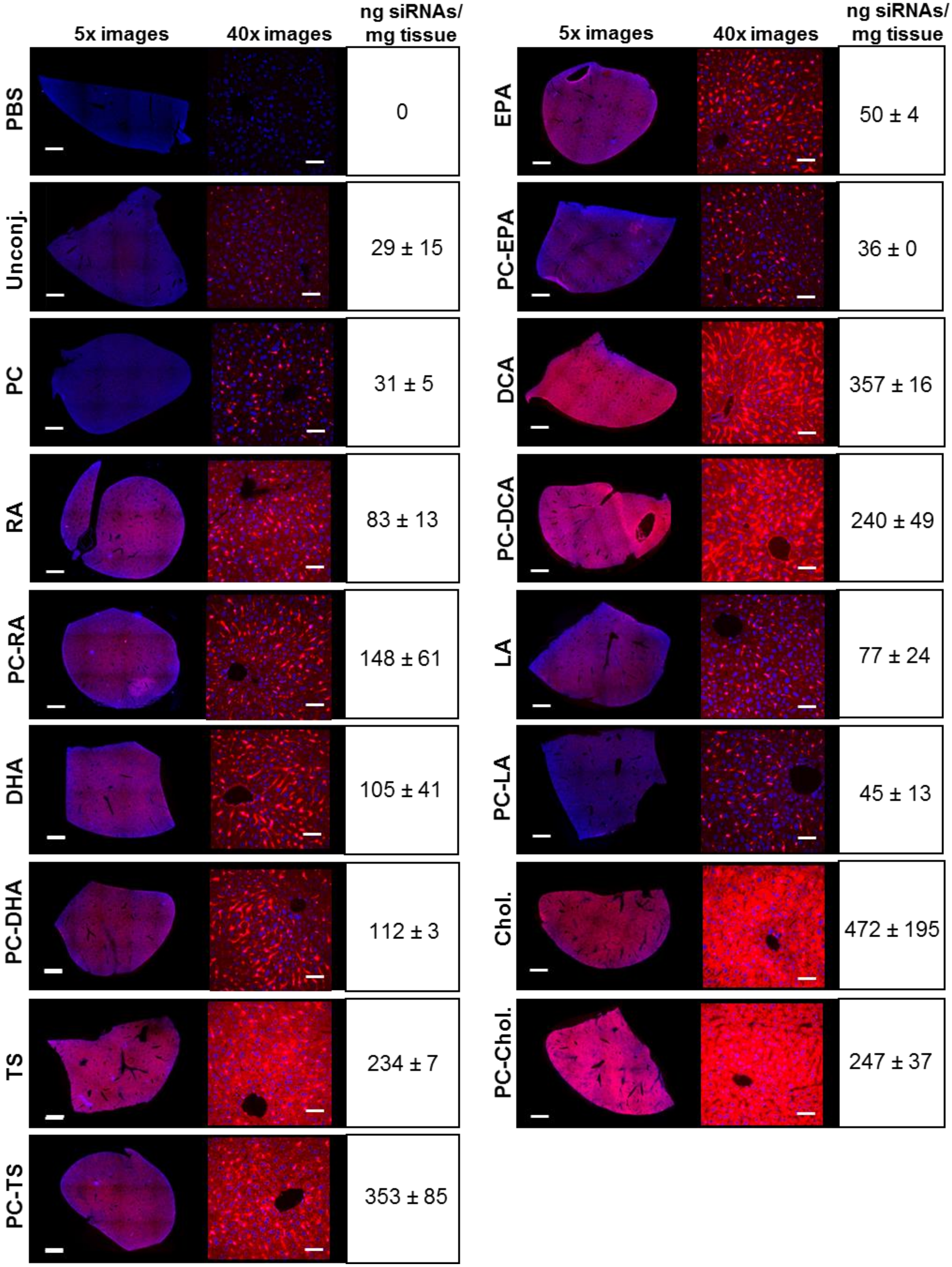

Supplementary Figure 4: Liver distribution of Cy3-conjugated siRNAs. Subcutaneous injection (FVB/N mice); 20 mg/kg; collection of tissues 48h after injection; n = 3 per conjugate. DAPI in blue; Cy3-siRNAs in red. 5x tiled arrays bar scale = 1 mm; 40x images bar scale = 50 µm; siRNA quantification by PNA hybridization assay (average of 3 animals ± SD).

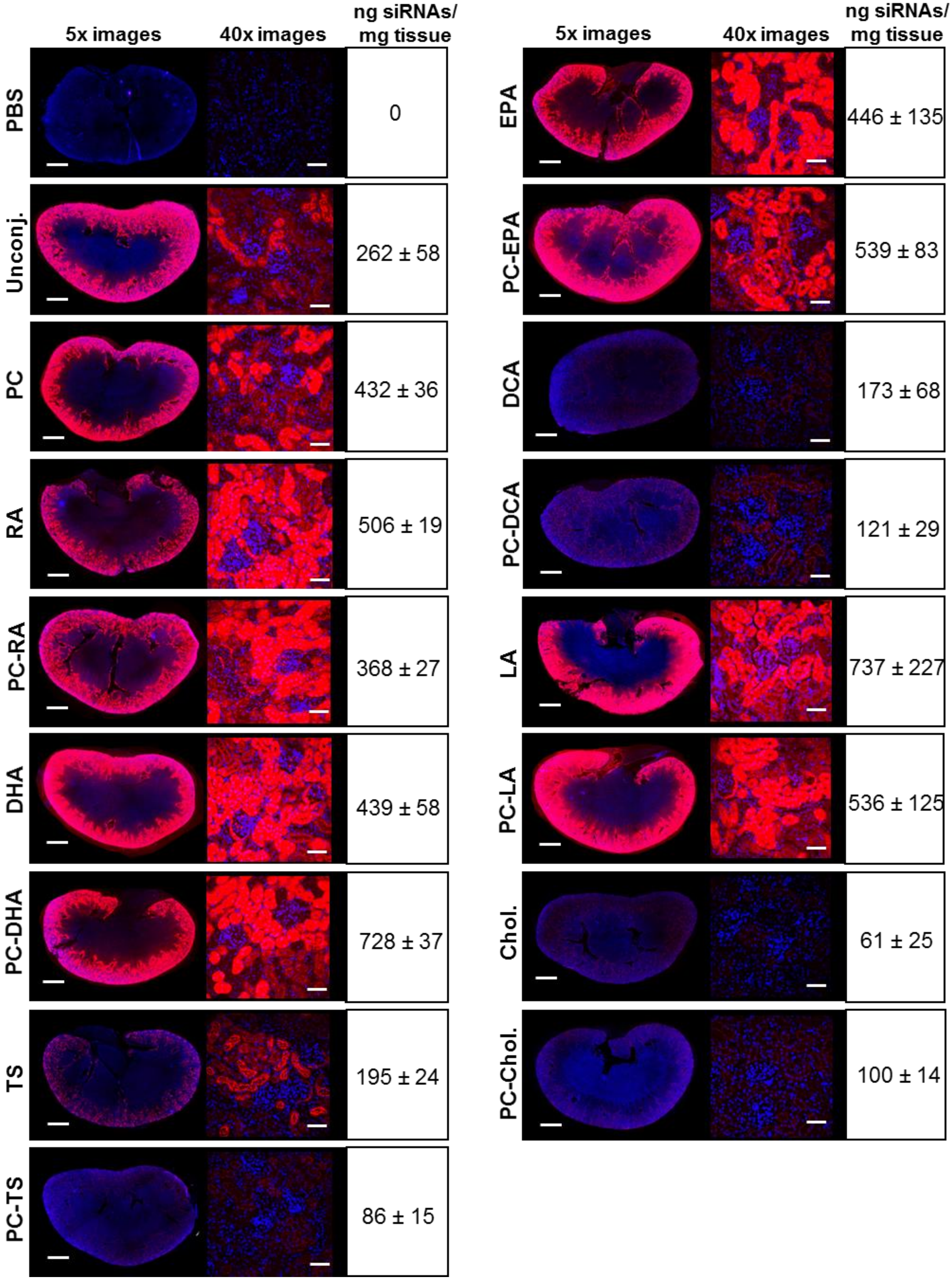

Supplementary Figure 5: Kidney distribution of Cy3-conjugated siRNAs. Subcutaneous injection (FVB/N mice); 20 mg/kg; collection of tissues 48h after injection; n = 3 per conjugate. DAPI in blue; Cy3-siRNAs in red. 5x tiled arrays bar scale = 1 mm; 40x images bar scale = 50 µm; siRNA quantification by PNA hybridization assay (average of 3 animals ± SD).

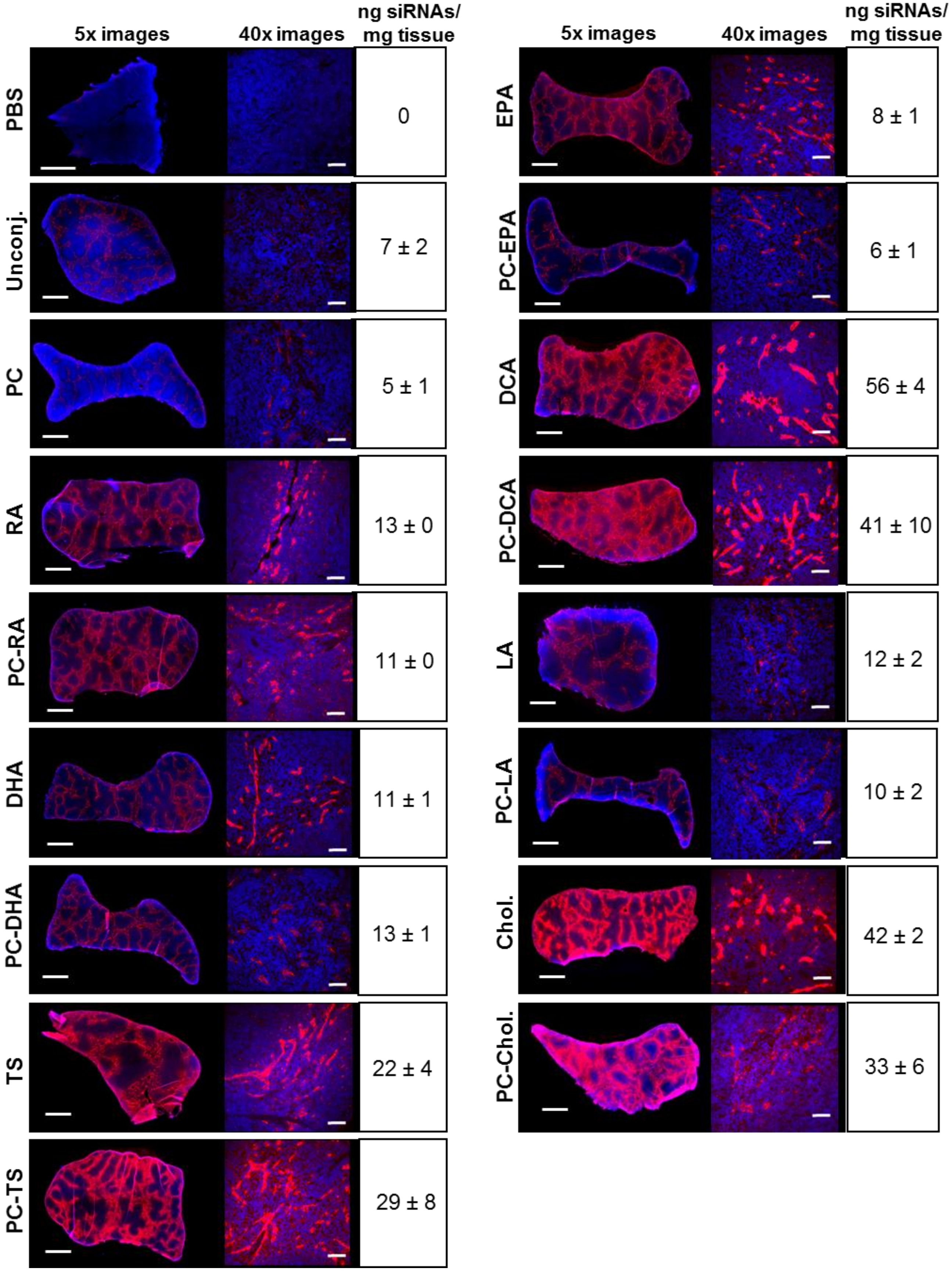

Supplementary Figure 6: Spleen distribution of Cy3-conjugated siRNAs. Subcutaneous injection (FVB/N mice); 20 mg/kg; collection of tissues 48h after injection; n = 3 per conjugate. DAPI in blue; Cy3-siRNAs in red. 5x tiled arrays bar scale = 1 mm; 40x images bar scale = 50 µm; siRNA quantification by PNA hybridization assay (average of 3 animals ± SD).

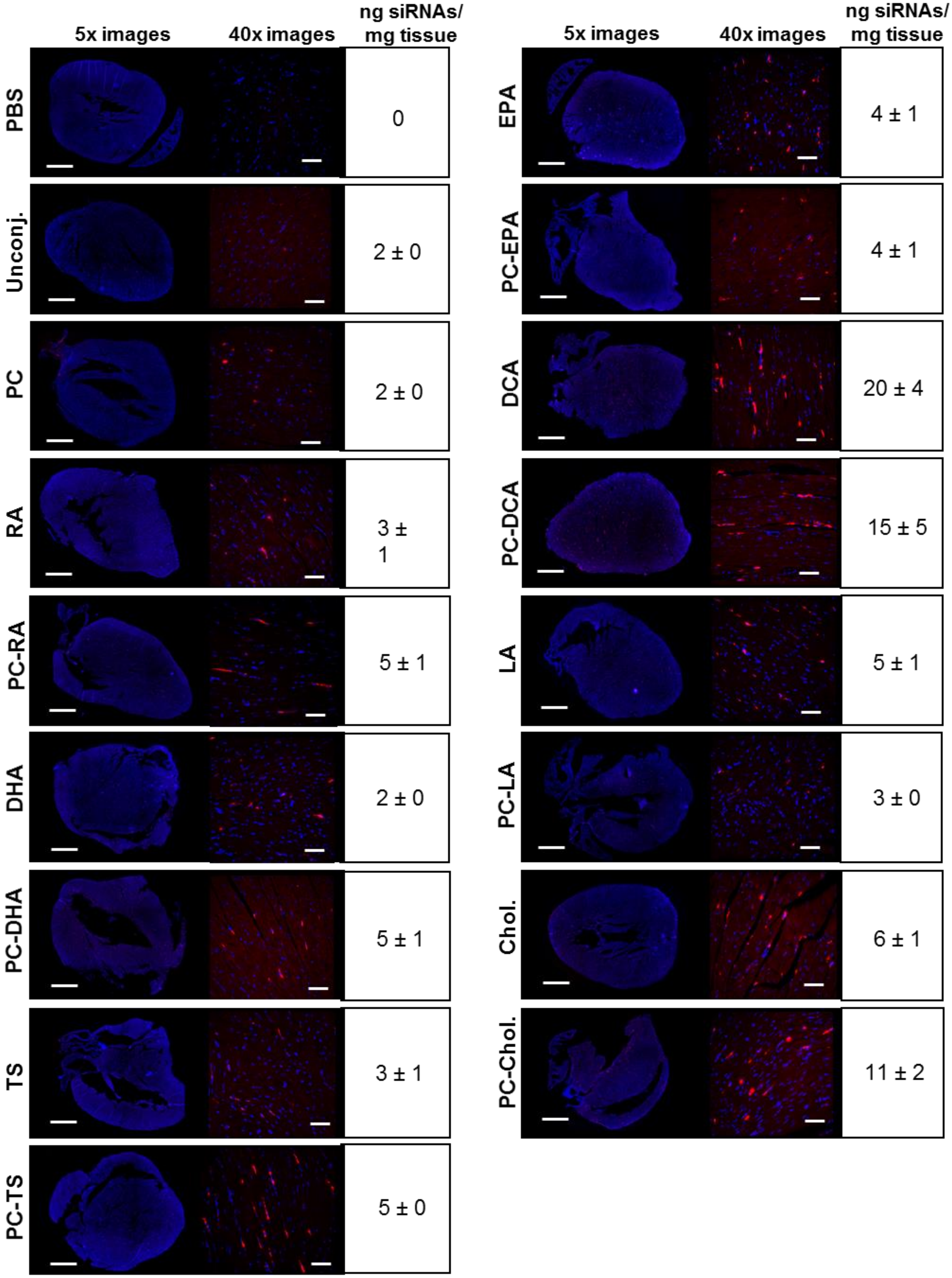

Supplementary Figure 7: Heart distribution of Cy3-conjugated siRNAs. Subcutaneous injection (FVB/N mice); 20 mg/kg; collection of tissues 48h after injection; n = 3 per conjugate. DAPI in blue; Cy3-siRNAs in red. 5x tiled arrays bar scale = 1 mm; 40x images bar scale = 50 µm; siRNA quantification by PNA hybridization assay (average of 3 animals ± SD).

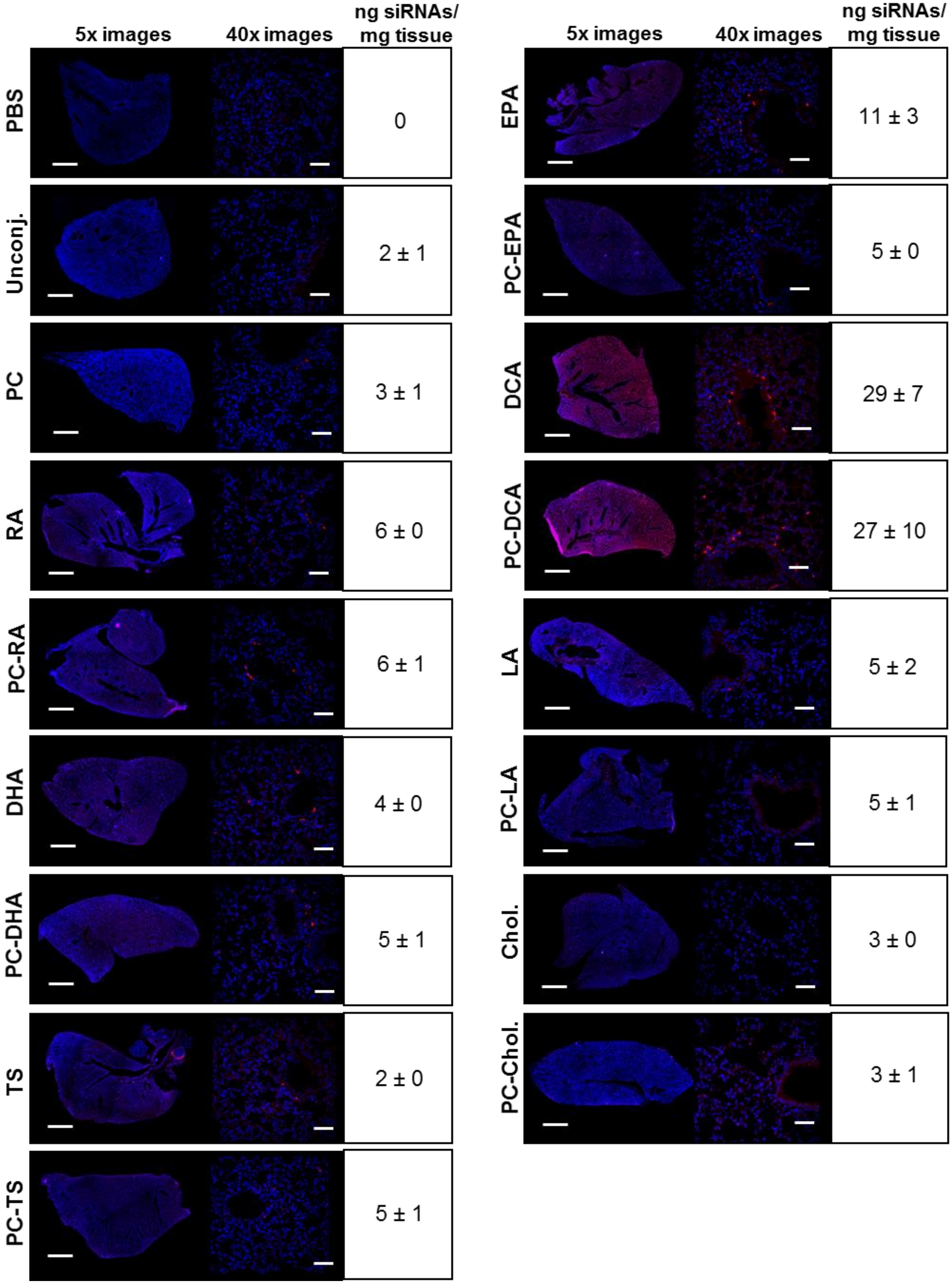

Supplementary Figure 8: Lung distribution of Cy3-conjugated siRNAs. Subcutaneous injection (FVB/N mice); 20 mg/kg; collection of tissues 48h after injection; n = 3 per conjugate. DAPI in blue; Cy3-siRNAs in red. 5x tiled arrays bar scale = 1 mm; 40x images bar scale = 50 µm; siRNA quantification by PNA hybridization assay (average of 3 animals ± SD).

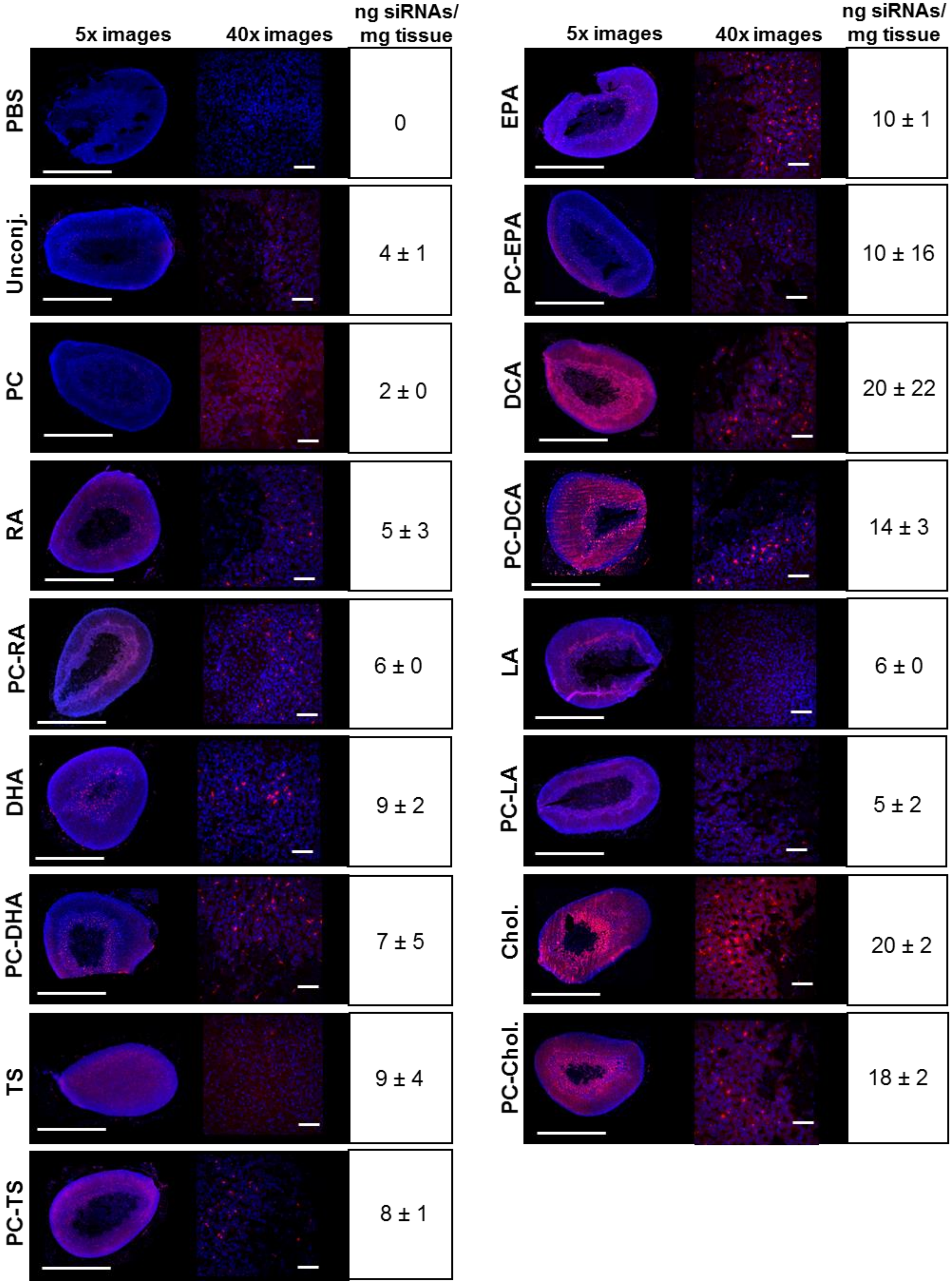

Supplementary Figure 9: Adrenal glands distribution of Cy3-conjugated siRNAs. Subcutaneous injection (FVB/N mice); 20 mg/kg; collection of tissues 48h after injection; n = 3 per conjugate. DAPI in blue; Cy3-siRNAs in red. 5x tiled arrays bar scale = 1 mm; 40x images bar scale = 50 μm; siRNA quantification by PNA hybridization assay (average of 3 animals ± SD).

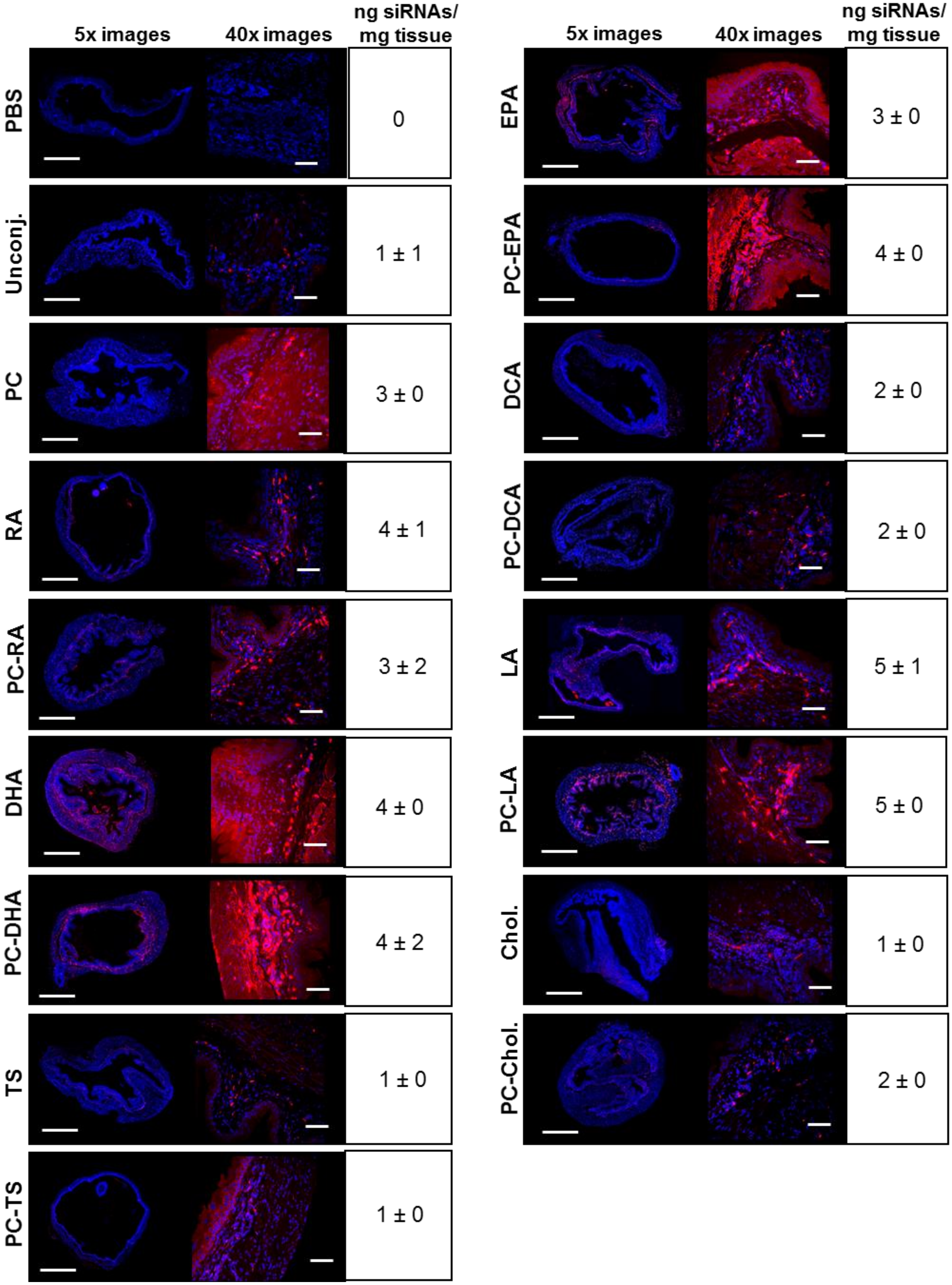

Supplementary Figure 10: Bladder distribution of Cy3-conjugated siRNAs. Subcutaneous injection (FVB/N mice); 20 mg/kg; collection of tissues 48h after injection; n = 3 per conjugate. DAPI in blue; Cy3-siRNAs in red. 5x tiled arrays bar scale = 1 mm; 40x images bar scale = 50 µm; siRNA quantification by PNA hybridization assay (average of 3 animals ± SD).

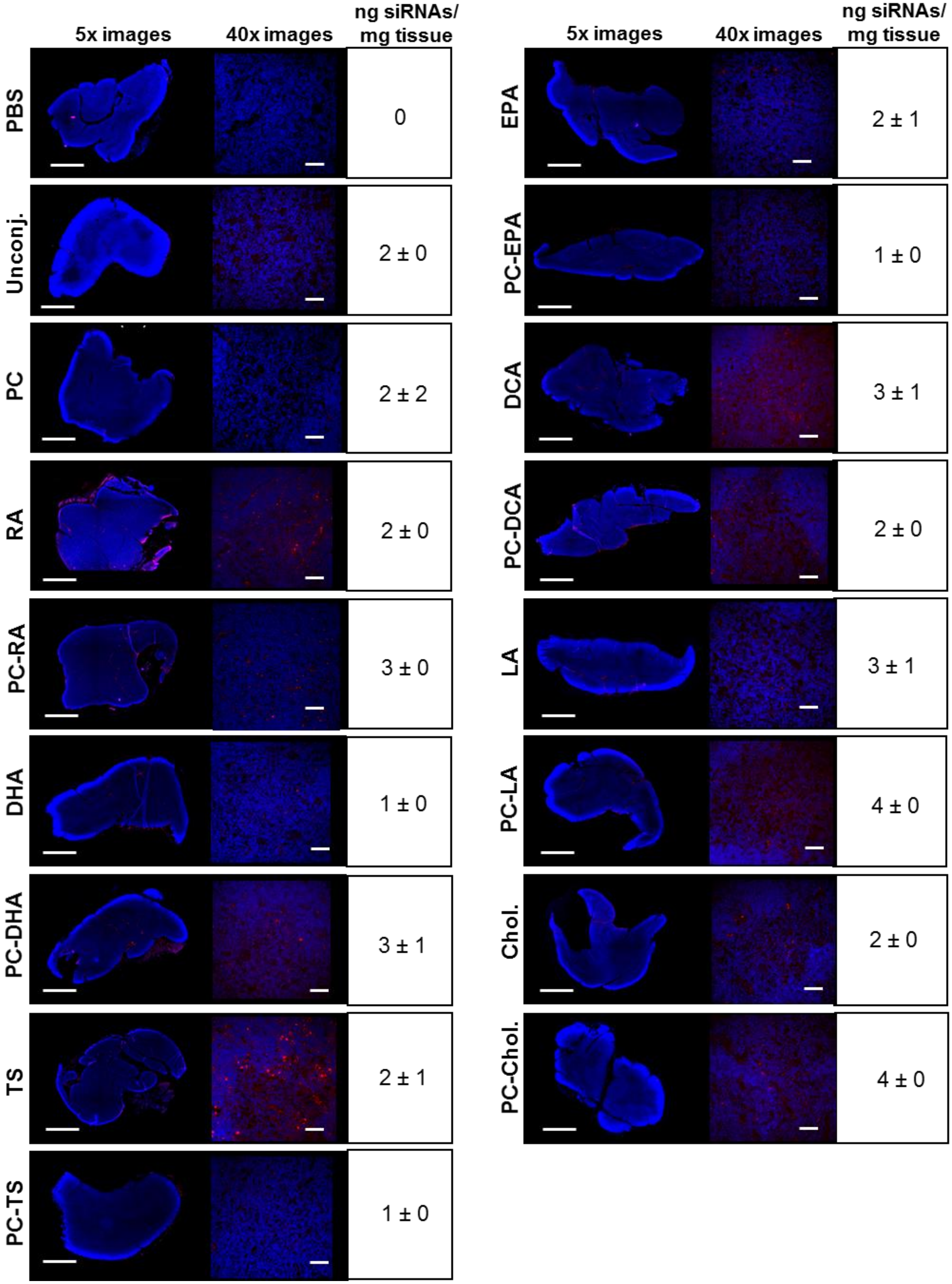

Supplementary Figure 11: Thymus distribution of Cy3-conjugated siRNAs. Subcutaneous injection (FVB/N mice); 20 mg/kg; collection of tissues 48h after injection; n = 3 per conjugate. DAPI in blue; Cy3-siRNAs in red. 5x tiled arrays bar scale = 1 mm; 40x images bar scale = 50 µm; siRNA quantification by PNA hybridization assay (average of 3 animals ± SD).

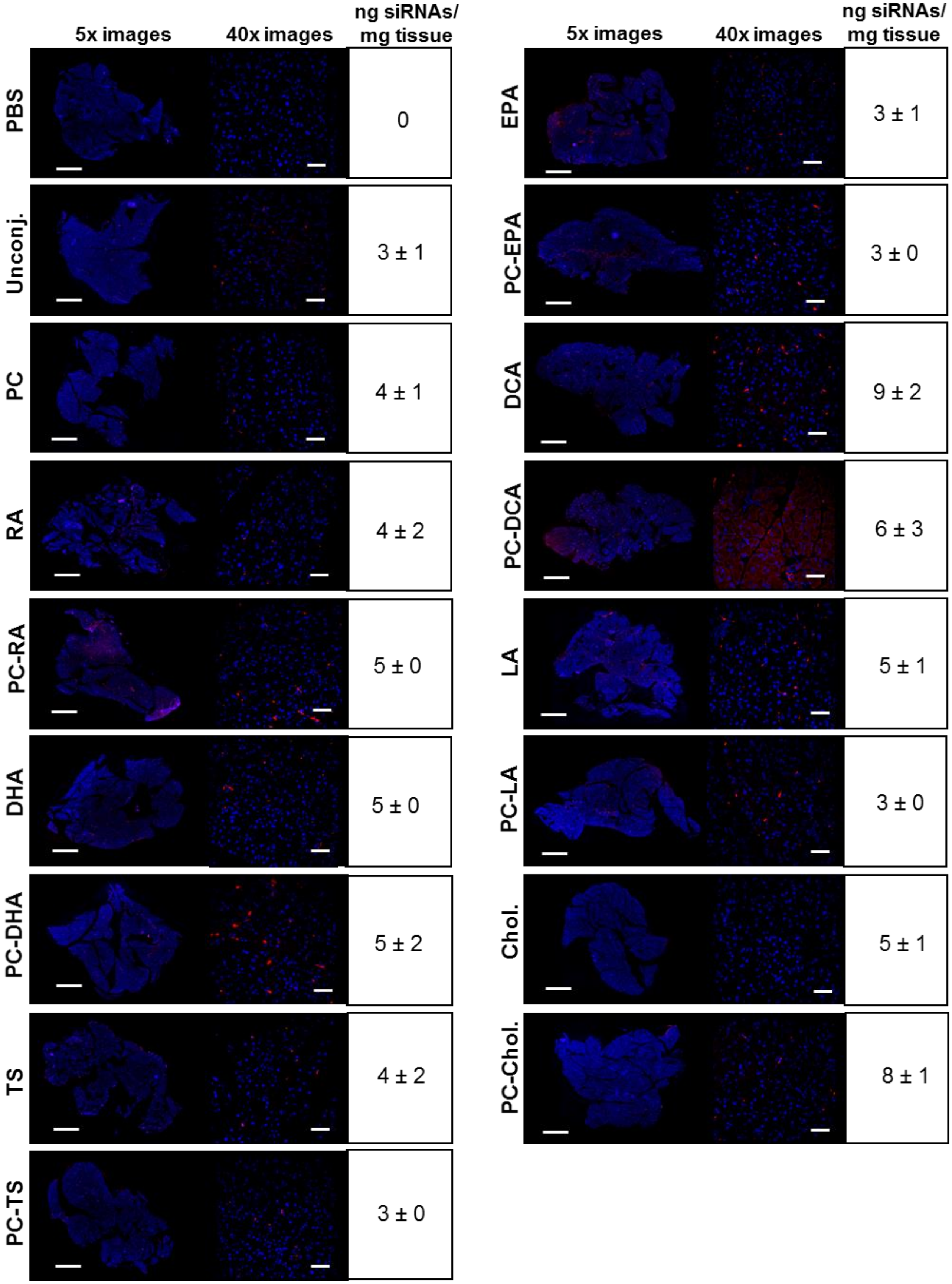

Supplementary Figure 12: Pancreas distribution of Cy3-conjugated siRNAs. Subcutaneous injection (FVB/N mice); 20 mg/kg; collection of tissues 48h after injection; n = 3 per conjugate. DAPI in blue; Cy3-siRNAs in red. 5x tiled arrays bar scale = 1 mm; 40x images bar scale = 50 µm; siRNA quantification by PNA hybridization assay (average of 3 animals ± SD).

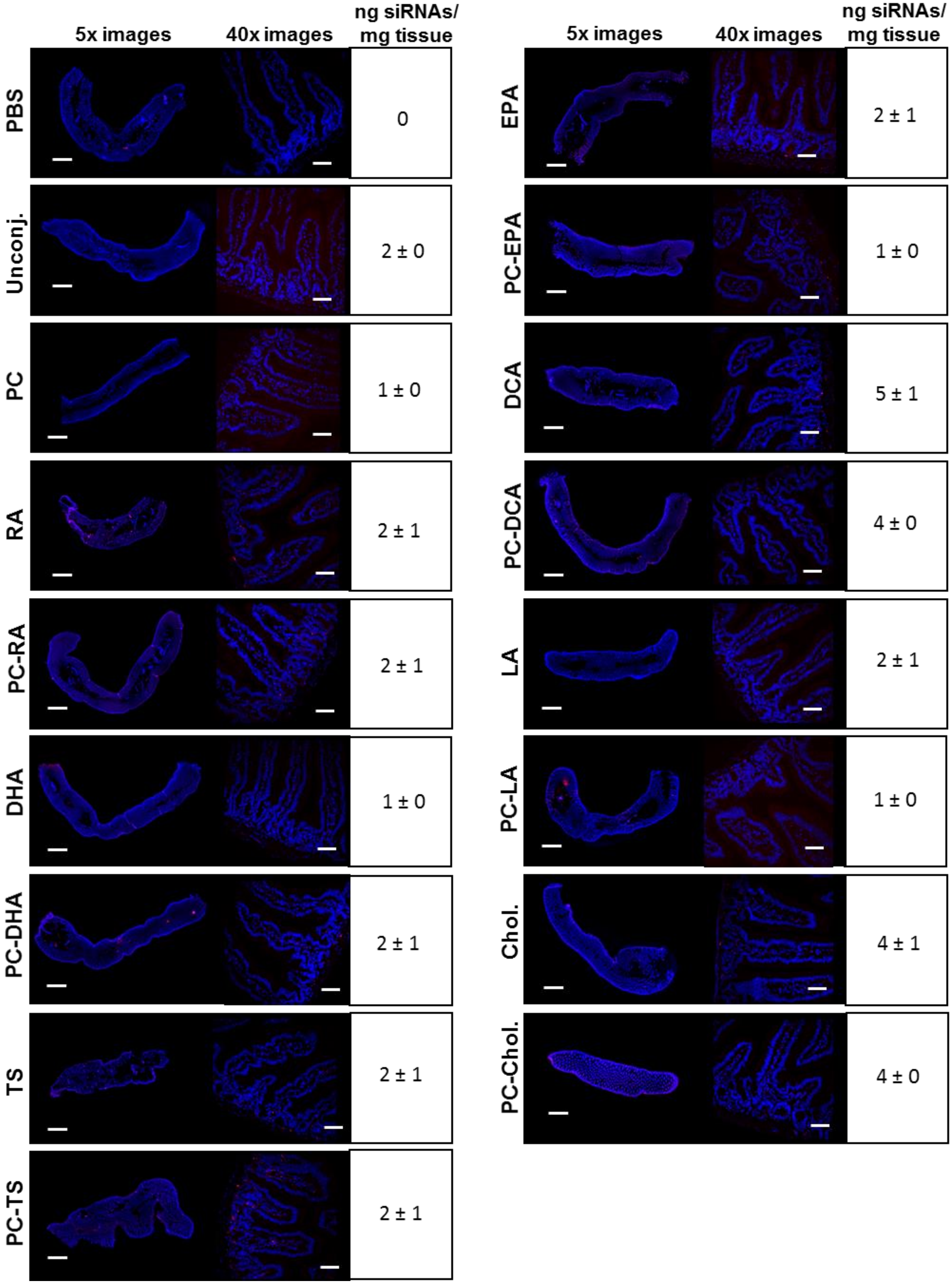

Supplementary Figure 13: Intestine distribution of Cy3-conjugated siRNAs. Subcutaneous injection (FVB/N mice); 20 mg/kg; collection of tissues 48h after injection; n = 3 per conjugate. DAPI in blue; Cy3-siRNAs in red. 5x tiled arrays bar scale = 1 mm; 40x images bar scale = 50 µm; siRNA quantification by PNA hybridization assay (average of 3 animals ± SD).

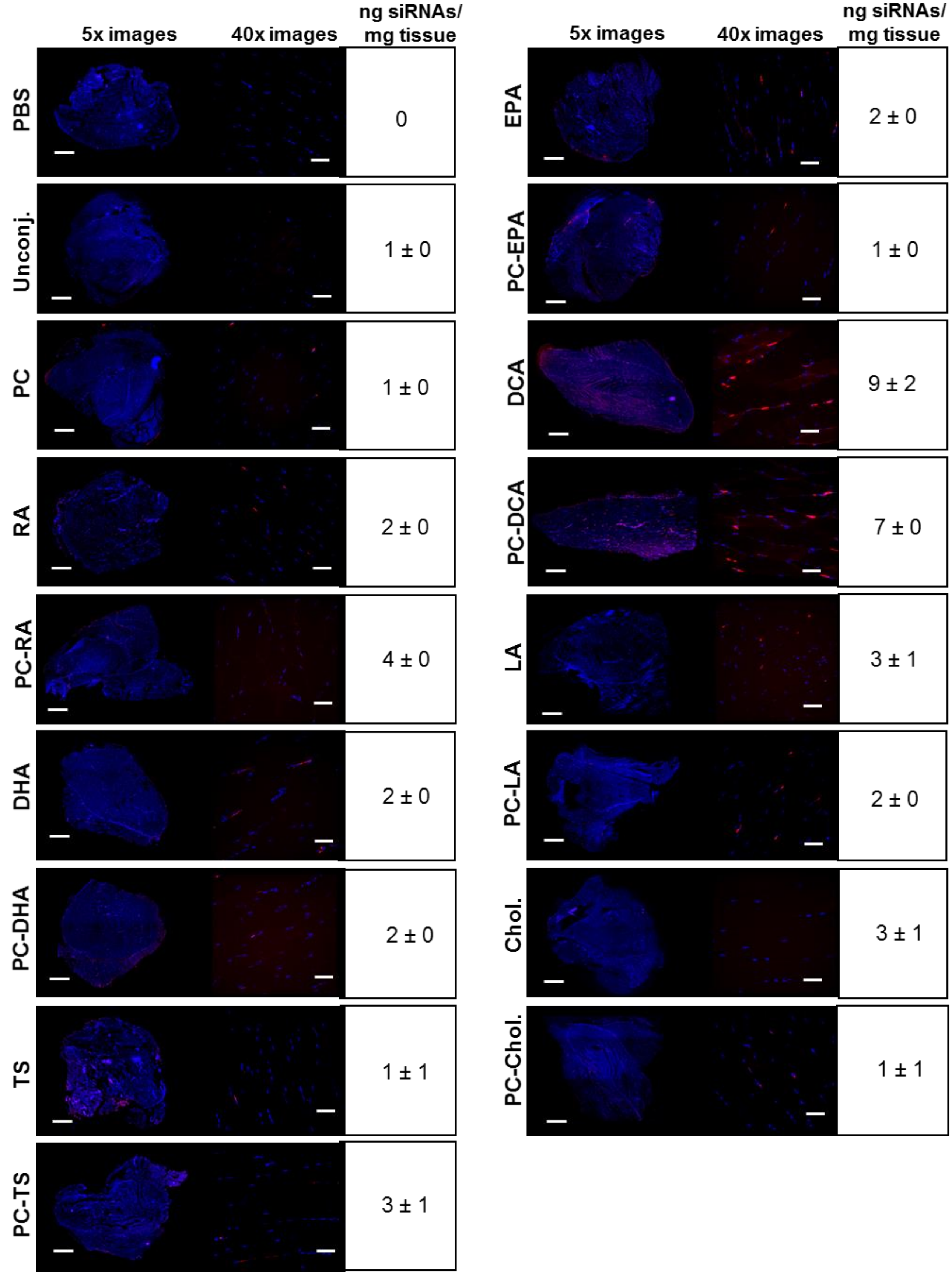

Supplementary Figure 14: Muscle distribution of Cy3-conjugated siRNAs. Subcutaneous injection (FVB/N mice); 20 mg/kg; collection of tissues 48h after injection; n = 3 per conjugate. DAPI in blue; Cy3-siRNAs in red. 5x tiled arrays bar scale = 1 mm; 40x images bar scale = 50 µm; siRNA quantification by PNA hybridization assay (average of 3 animals ± SD).

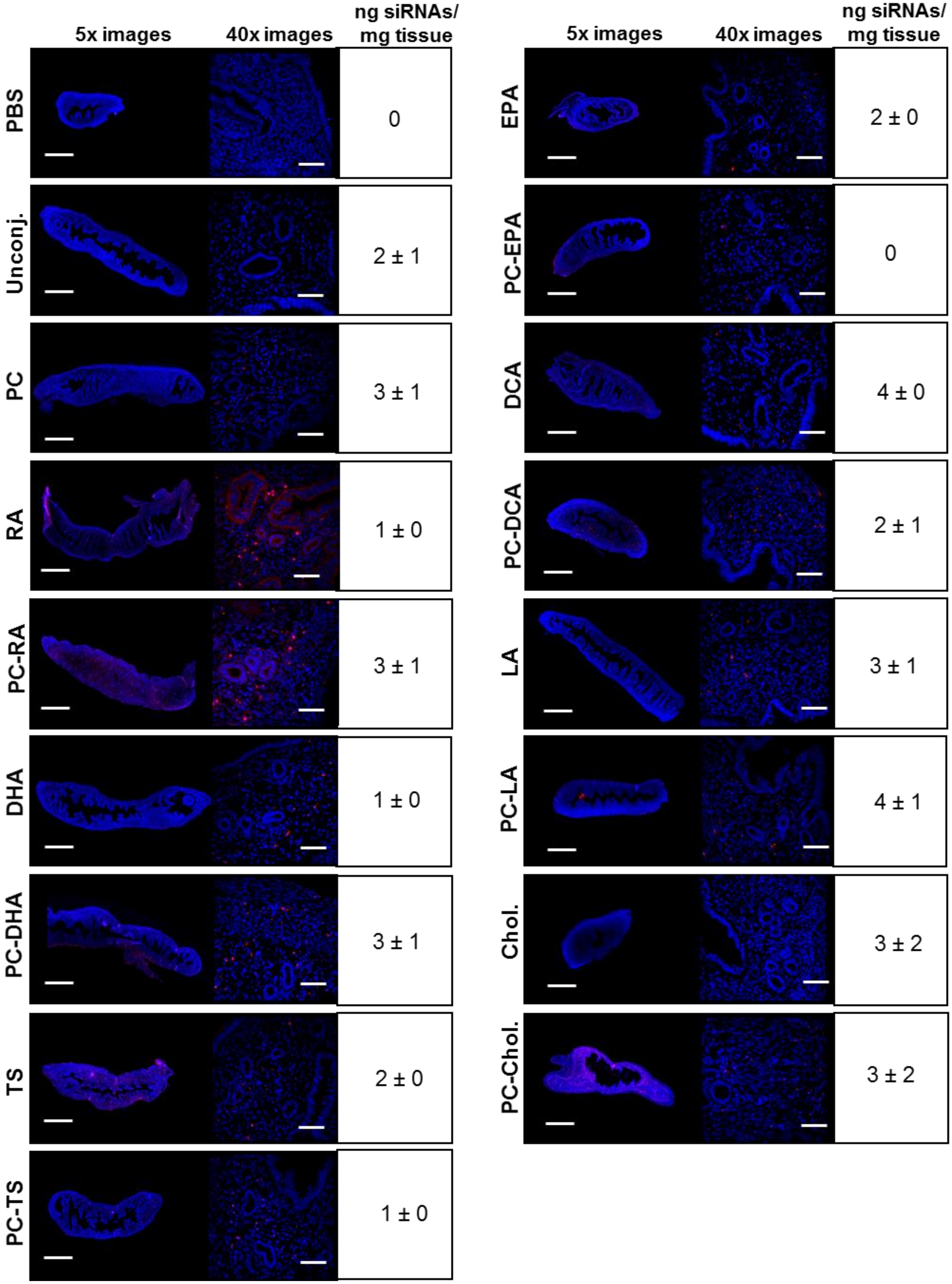

Supplementary Figure 15: Fallopian tube distribution of Cy3-conjugated siRNAs. Subcutaneous injection (FVB/N mice); 20 mg/kg; collection of tissues 48h after injection; n = 3 per conjugate. DAPI in blue; Cy3-siRNAs in red. 5x tiled arrays bar scale = 1 mm; 40x images bar scale = 50 µm; siRNA quantification by PNA hybridization assay (average of 3 animals ± SD).

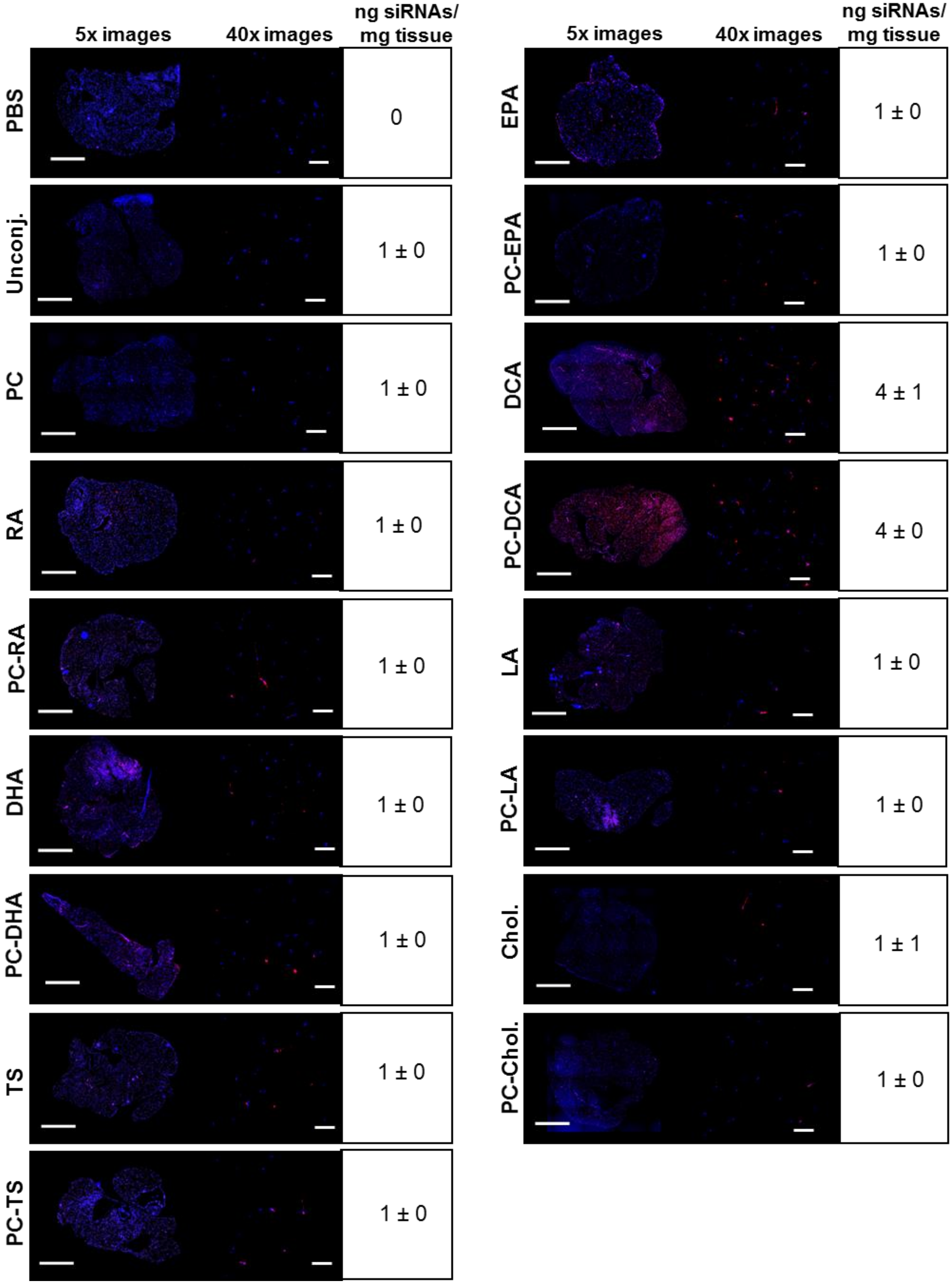

Supplementary Figure 16: Fat distribution of Cy3-conjugated siRNAs. Subcutaneous injection (FVB/N mice); 20 mg/kg; collection of tissues 48h after injection; n = 3 per conjugate. DAPI in blue; Cy3-siRNAs in red. 5x tiled arrays bar scale = 1 mm; 40x images bar scale = 50 µm; siRNA quantification by PNA hybridization assay (average of 3 animals ± SD).

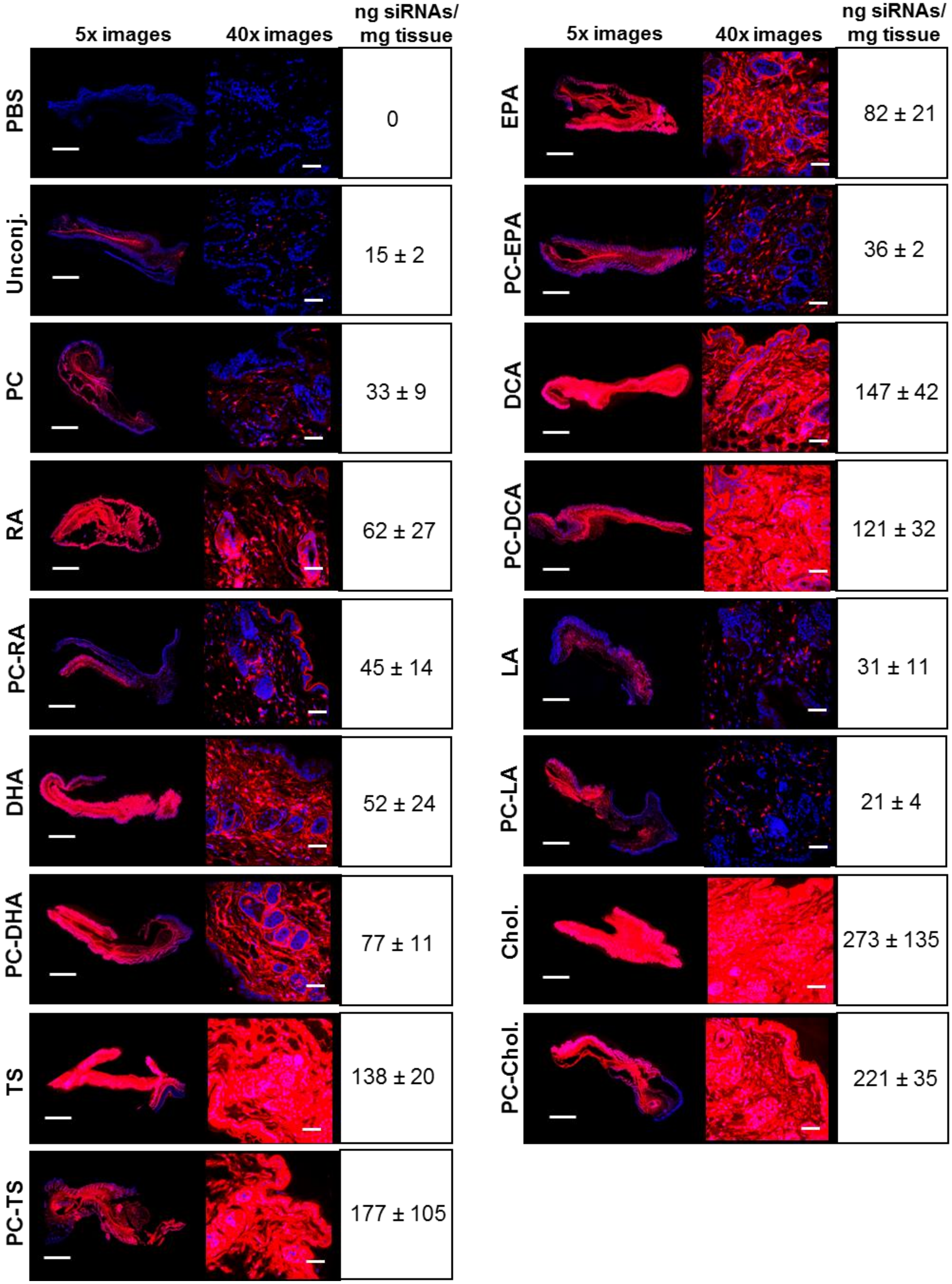

Supplementary Figure 17: Skin (site of injection) of Cy3-conjugated siRNAs. Subcutaneous injection (FVB/N mice); 20 mg/kg; collection of tissues 48h after injection; n = 3 per conjugate. DAPI in blue; Cy3-siRNAs in red. 5x tiled arrays bar scale = 1 mm; 40x images bar scale = 50 µm; siRNA quantification by PNA hybridization assay (average of 3 animals ± SD).

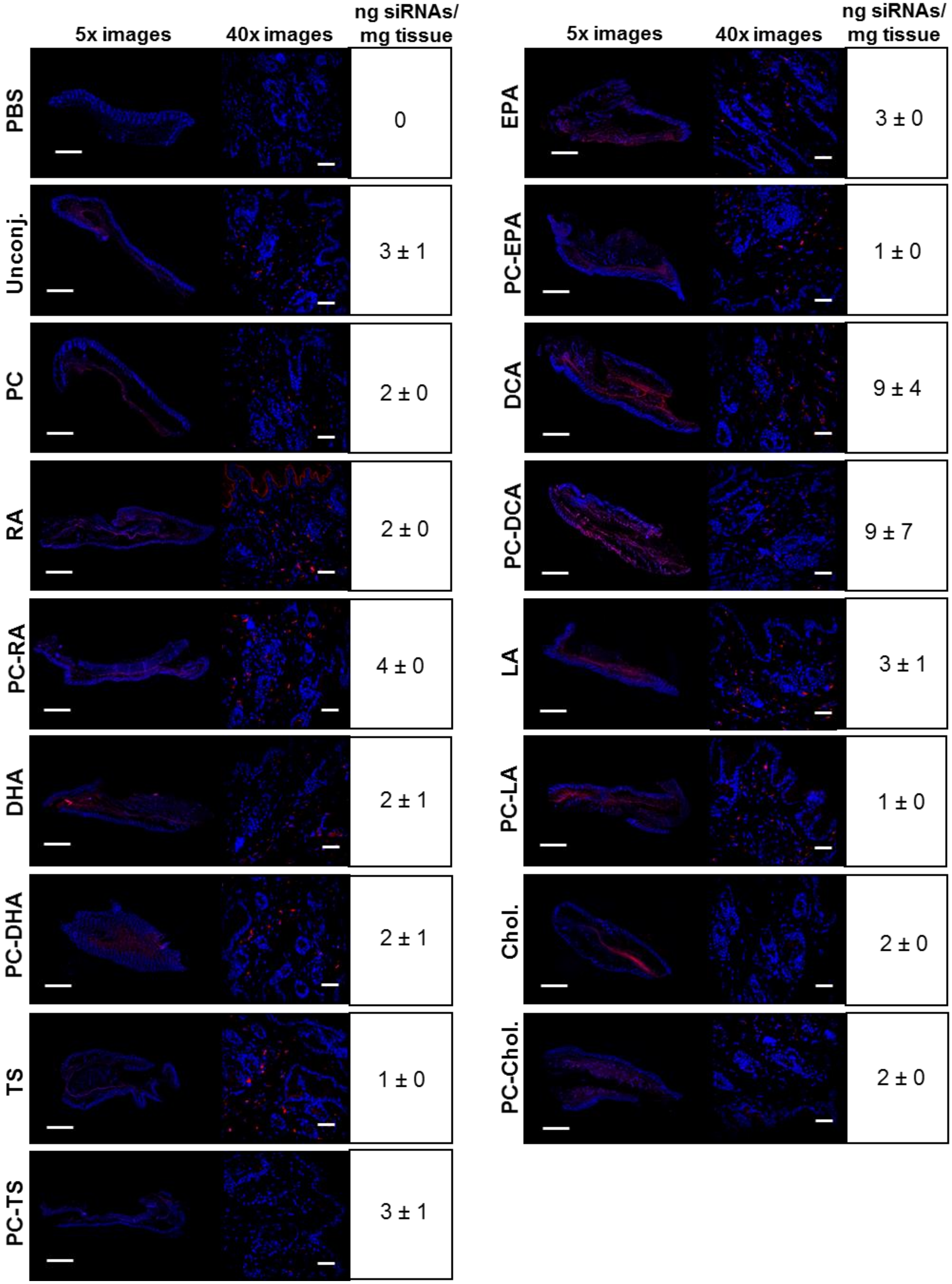

Supplementary Figure 18: Skin of Cy3-conjugated siRNAs. Subcutaneous injection (FVB/N mice); 20 mg/kg; collection of tissues 48h after injection; n = 3 per conjugate. DAPI in blue; Cy3-siRNAs in red. 5x tiled arrays bar scale = 1 mm; 40x images bar scale = 50 µm; siRNA quantification by PNA hybridization assay (average of 3 animals ± SD).

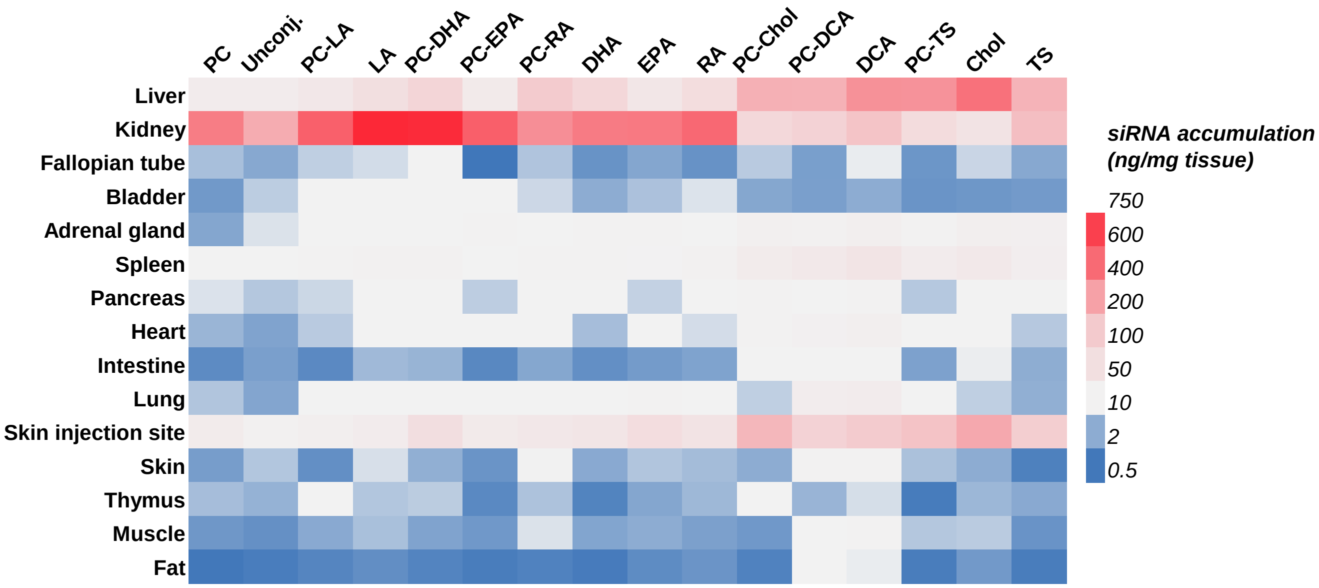

Supplementary Figure 19. The structure of the conjugate defines siRNA distribution. Heat map representation of the tissue concentrations of siRNA antisense strands. Data represent the average of 3 experiments. Subcutaneous injection (FVB/N mice) ; 20 mg/kg ; collection of tissues 48 h after injection ; n = 3 per conjugate.

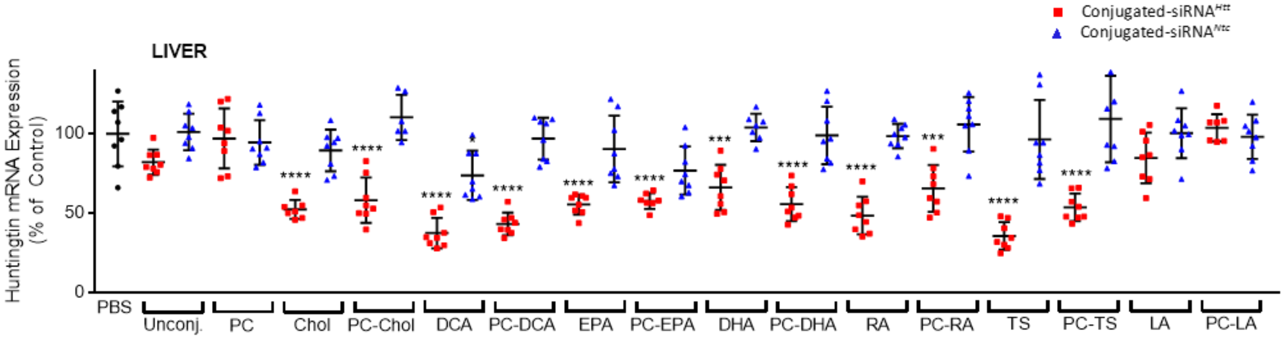

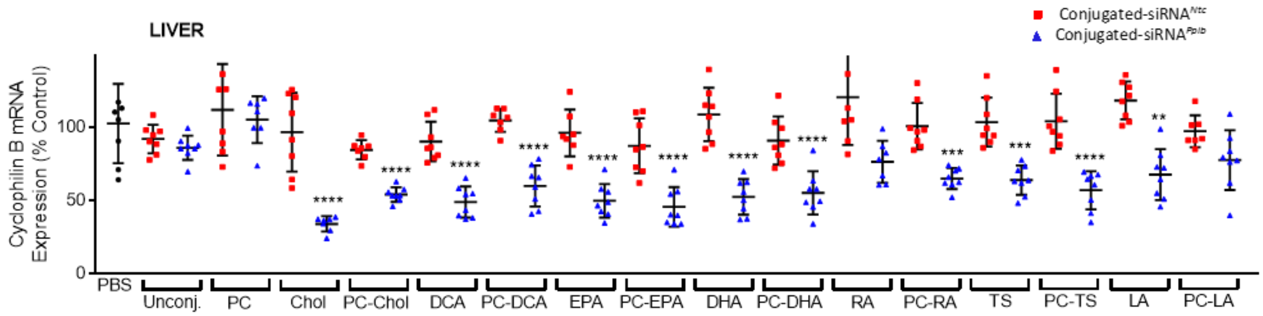

	PBS	Unconj.	PC	Chol	PC-Chol	DCA	PC-DCA	EPA	PC-EPA	DHA	PC-DHA	RA	PC-RA	TS	PC-TS	LA	PC-LA
**Huntingtin mRNA silencing (% compared to *Ntc*)**	0 ± 20	19 ± 8	0 ± 19	37 ± 6	52 ± 14	36 ± 9	54 ± 7	35 ± 6	19 ± 5	38 ± 14	43 ± 11	50 ± 12	40 ± 15	61 ± 9	56 ± 9	16 ± 16	0 ± 9
**Significance (compared to *Ntc*)**	/	ns	ns	***	****	***	****	**	ns	***	****	****	****	****	****	ns	ns
**Cyclophilin B mRNA silencing (% compared to *Ntc*)**	0 ± 27	6 ± 8	7 ± 16	63 ± 5	30 ± 5	41 ± 11	45 ± 14	46 ± 12	41 ± 14	56 ± 12	36 ± 15	44 ± 14	36 ± 7	40 ± 10	47 ± 13	51 ± 17	20 ± 20
**Significance (compared to *Ntc*)**	/	ns	ns	****	**	***	***	****	***	****	**	***	**	**	****	****	ns

Supplementary Figure 20: Efficacy of conjugated siRNAs in liver. Subcutaneous injection (FVB/N mice); 20 mg/kg; collection of tissues one week after injection; n = 16 per gene and per conjugate (included non-targeting controls or *Ntc*). Huntingtin (*Htt*) (upper panel) and Cyclophilin B (*Ppib*) (lower panel) mRNA levels were measured using QuantiGene® (Affymetrix), normalized to a housekeeping gene, *Hprt* (Hypoxanthine-guanine phosphoribosyl transferase), and presented as percent of PBS (Phosphate buffered saline) control (mean ± SD). Data analysis: Outliners define with Grubb's method (alpha = 0.1%); Multiple comparisons = One-way ANOVA, Bonferroni test (****P<0.0001, ***P<0.001, **P<0.01, *P<0.1). The table indicates the average of inhibition percentages (n = 8) and significances for each target and conjugate compared to *Ntc* (mean ± SD; ns = non-significant).

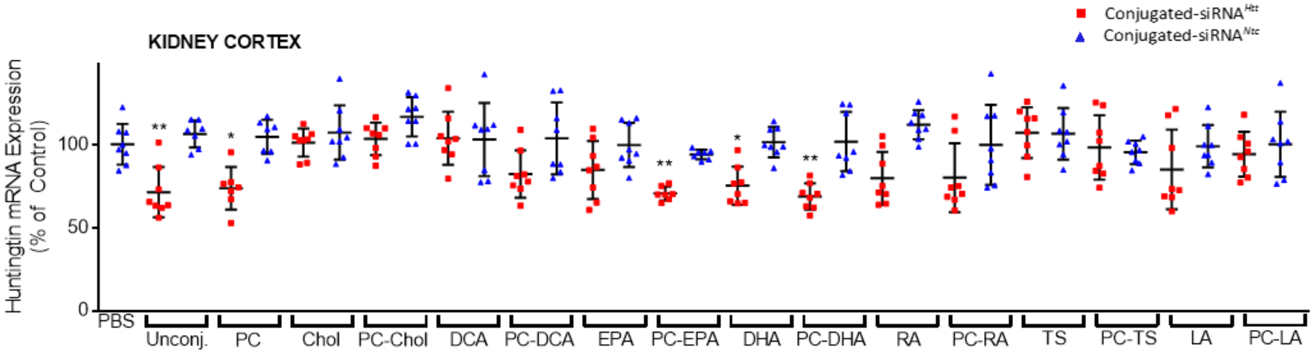

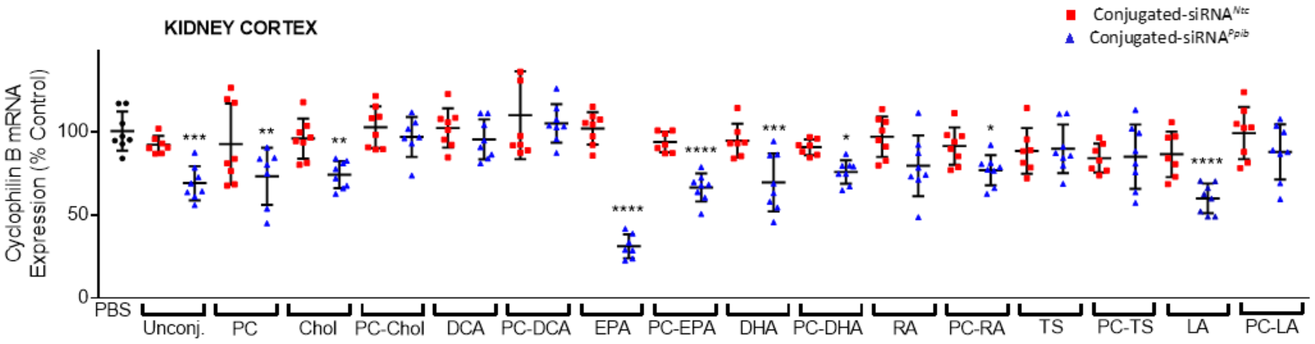

	PBS	Unconj.	PC	Chol	PC-Chol	DCA	PC-DCA	EPA	PC-EPA	DHA	PC-DHA	RA	PC-RA	TS	PC-TS	LA	PC-LA
**Huntingtin mRNA silencing (% compared to *Ntc*)**	0 ± 12	35 ± 15	31 ± 13	6 ± 8	13 ± 10	0 ± 16	22 ± 14	15 ± 17	24 ± 4	26 ± 11	33 ± 8	32 ± 16	20 ± 21	0 ± 15	0 ± 19	14 ± 24	6 ± 14
**Significance (compared to *Ntc*)**	/	**	*	ns	ns	ns	ns	ns	*	ns	**	*	ns	ns	ns	ns	ns
**Cyclophilin B mRNA silencing (% compared to *Ntc*)**	0 ± 12	23 ± 10	11 ± 24	22 ± 8	6 ± 12	7 ± 12	5 ± 12	71 ± 7	27 ± 8	25 ± 17	15 ± 7	18 ± 18	15 ± 9	0 ± 15	0 ± 19	27 ± 9	11 ± 17
**Significance (compared to *Ntc*)**	/	*	ns	ns	ns	ns	ns	****	**	*	ns	ns	ns	ns	ns	*	ns

Supplementary Figure 21: Efficacy of conjugated siRNAs in kidney (cortex). Subcutaneous injection (FVB/N mice); 20 mg/kg; collection of tissues one week after injection; n = 16 per gene and per conjugate (included non-targeting controls or *Ntc*). Huntingtin (*Htt*) (upper panel) and Cyclophilin B (*Ppib*) (lower panel) mRNA levels were measured using QuantiGene® (Affymetrix), normalized to a housekeeping gene, *Hprt* (Hypoxanthine-guanine phosphoribosyl transferase), and presented as percent of PBS (Phosphate buffered saline) control (mean ± SD). Data analysis: Outliners define with Grubb's method (alpha = 0.1%); Multiple comparisons = One-way ANOVA, Bonferroni test (****P<0.0001, ***P<0.001, **P<0.01, *P<0.1). The table indicates the average of inhibition percentages (n = 8) and significances for each target and conjugate compared to *Ntc* (mean ± SD; ns = non-significant).

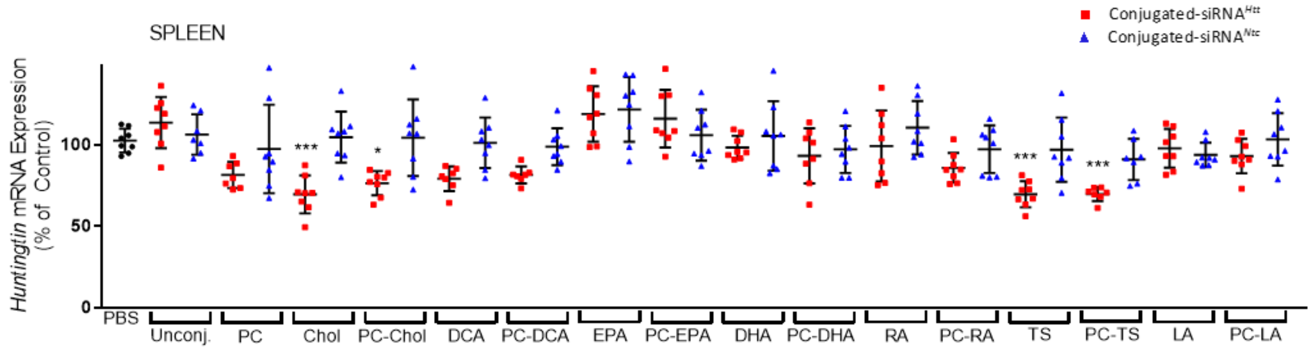

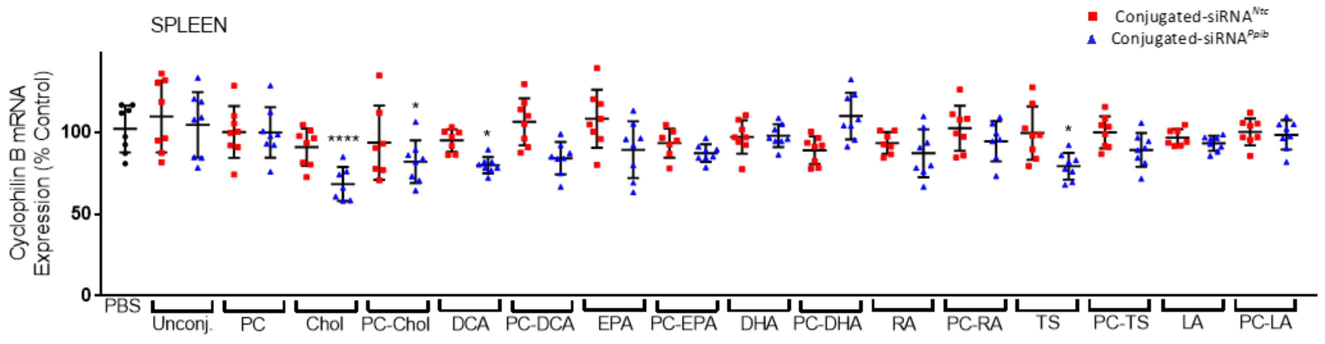

	PBS	Unconj.	PC	Chol	PC-Chol	DCA	PC-DCA	EPA	PC-EPA	DHA	PC-DHA	RA	PC-RA	TS	PC-TS	LA	PC-LA
Huntingtin mRNA silencing (% compared to *Ntc*)	0 ± 7	0 ± 16	16 ± 6	35 ± 12	28 ± 7	22 ± 8	17 ± 5	3 ± 17	0 ± 18	7 ± 7	4 ± 17	11 ± 22	11 ± 9	27 ± 8	21 ± 4	0 ± 12	10 ± 11
Significance (compared to *Ntc*)	/	ns	ns	**	ns	*	*	ns	ns	ns	ns	ns	ns	*	*	ns	ns
Cyclophilin B mRNA silencing (% compared to *Ntc*)	0 ± 14	5 ± 20	0 ± 15	23 ± 10	12 ± 13	15 ± 5	22 ± 10	19 ± 17	6 ± 5	0 ± 7	0 ± 14	6 ± 15	8 ± 12	20 ± 8	11 ± 10	4 ± 5	2 ± 9
Significance (compared to *Ntc*)	/	ns	ns	*	ns	*	**	ns	ns	ns	ns	ns	ns	*	ns	ns	ns

Supplementary Figure 22: Efficacy of conjugated siRNAs in spleen. Subcutaneous injection (FVB/N mice); 20 mg/kg; collection of tissues one week after injection; n = 16 per gene and per conjugate (included non-targeting controls or *Ntc*). Huntingtin (*Htt*) (upper panel) and Cyclophilin B (*Ppib*) (lower panel) mRNA levels were measured using QuantiGene® (Affymetrix), normalized to a housekeeping gene, *Hprt* (Hypoxanthine-guanine phosphoribosyl transferase), and presented as percent of PBS (Phosphate buffered saline) control (mean ± SD). Data analysis: Outliners define with Grubb's method (alpha = 0.1%); Multiple comparisons = One-way ANOVA, Bonferroni test (****P<0.0001, ***P<0.001, **P<0.01, *P<0.1). The table indicates the average of inhibition percentages (n = 8) and significances for each target and conjugate compared to *Ntc* (mean ± SD; ns = non-significant).

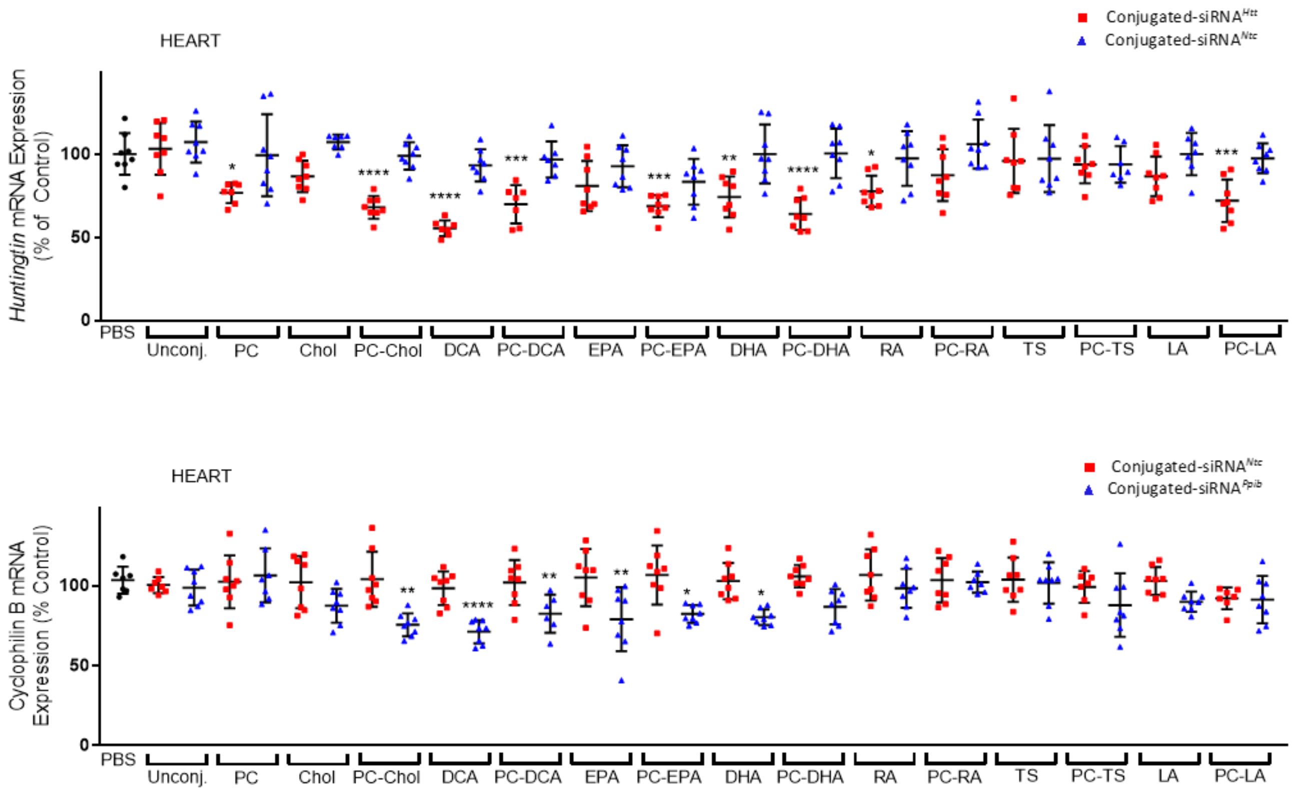

	PBS	Unconj.	PC	Chol	PC-Chol	DCA	PC-DCA	EPA	PC-EPA	DHA	PC-DHA	RA	PC-RA	TS	PC-TS	LA	PC-LA
**Huntingtin mRNA silencing (% compared to *Ntc*)**	0 ± 12	4 ± 16	23 ± 6	21 ± 9	31 ± 7	38 ± 5	20 ± 13	12 ± 15	15 ± 7	26 ± 12	36 ± 10	20 ± 9	19 ± 16	1 ± 19	0 ± 11	13 ± 12	25 ± 13
**Significance (compared to *Ntc*)**	/	ns	ns	*	**	****	**	ns	ns	**	****	ns	ns	ns	ns	ns	*
**Cyclophilin B mRNA silencing (% compared to *Ntc*)**	0 ± 8	2 ± 11	0 ± 17	15 ± 11	29 ± 7	27 ± 7	11 ± 19	26 ± 20	24 ± 5	23 ± 5	19 ± 11	8 ± 12	1 ± 7	2 ± 13	11 ± 20	13 ± 6	1 ± 15
**Significance (compared to *Ntc*)**	/	ns	ns	ns	**	**	ns	*	ns	ns	ns	ns	ns	ns	ns	ns	ns

Supplementary Figure 23: Efficacy of conjugated siRNAs in heart. Subcutaneous injection (FVB/N mice); 20 mg/kg; collection of tissues one week after injection; n = 16 per gene and per conjugate (included non-targeting controls or *Ntc*). Huntingtin (*Htt*) (upper panel) and Cyclophilin B (*Ppib*) (lower panel) mRNA levels were measured using QuantiGene® (Affymetrix), normalized to a housekeeping gene, *Hprt* (Hypoxanthine-guanine phosphoribosyl transferase), and presented as percent of PBS (Phosphate buffered saline) control (mean ± SD). Data analysis: Outliners define with Grubb's method (alpha = 0.1%); Multiple comparisons = One-way ANOVA, Bonferroni test (****P<0.0001, ***P<0.001, **P<0.01, *P<0.1). The table indicates the average of inhibition percentages (n = 8) and significances for each target and conjugate compared to *Ntc* (mean ± SD; ns = non-significant).

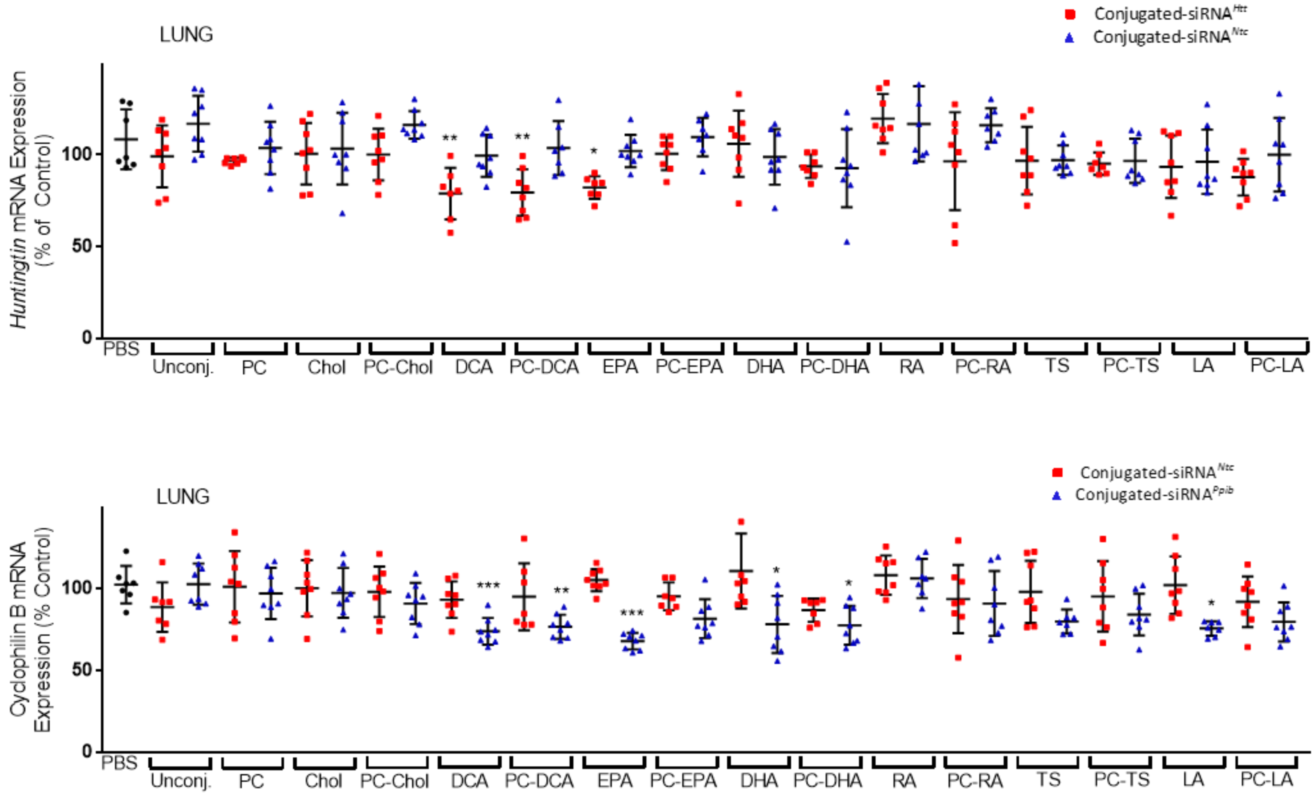

	PBS	Unconj.	PC	Chol	PC-Chol	DCA	PC-DCA	EPA	PC-EPA	DHA	PC-DHA	RA	PC-RA	TS	PC-TS	LA	PC-LA
**Huntingtin mRNA silencing (% compared to *Ntc*)**	0 ± 16	18 ± 17	7 ± 2	3 ± 17	16 ± 14	18 ± 15	24 ± 13	20 ± 6	9 ± 9	0 ± 18	0 ± 6	0 ± 13	20 ± 27	0 ± 18	2 ± 6	3 ± 17	12 ± 10
**Significance (compared to *Ntc*)**	/	ns	ns	ns	ns	*	*	*	ns	ns	ns	ns	ns	ns	ns	ns	ns
**Cyclophilin B mRNA silencing (% compared to *Ntc*)**	0 ± 11	0 ± 13	4 ± 16	3 ± 15	7 ± 13	19 ± 8	15 ± 7	35 ± 5	14 ± 12	33 ± 17	9 ± 12	2 ± 12	3 ± 20	18 ± 7	11 ± 12	27 ± 4	12 ± 12
**Significance (compared to *Ntc*)**	/	ns	ns	ns	ns	*	ns	***	ns	**	ns	ns	ns	ns	ns	ns	ns

Supplementary Figure 24: Efficacy of conjugated siRNAs in lung. Subcutaneous injection (FVB/N mice); 20 mg/kg; collection of tissues one week after injection; n = 16 per gene and per conjugate (included non-targeting controls or *Ntc*). Huntingtin (*Htt*) (upper panel) and Cyclophilin B (*Ppib*) (lower panel) mRNA levels were measured using QuantiGene® (Affymetrix), normalized to a housekeeping gene, *Hprt* (Hypoxanthine-guanine phosphoribosyl transferase), and presented as percent of PBS (Phosphate buffered saline) control (mean ± SD). Data analysis: Outliners define with Grubb's method (alpha = 0.1%); Multiple comparisons = One-way ANOVA, Bonferroni test (***P<0.001, **P<0.01, *P<0.1). The table indicates the average of inhibition percentages (n = 8) and significances for each target and conjugate compared to *Ntc* (mean ± SD; ns = non-significant).

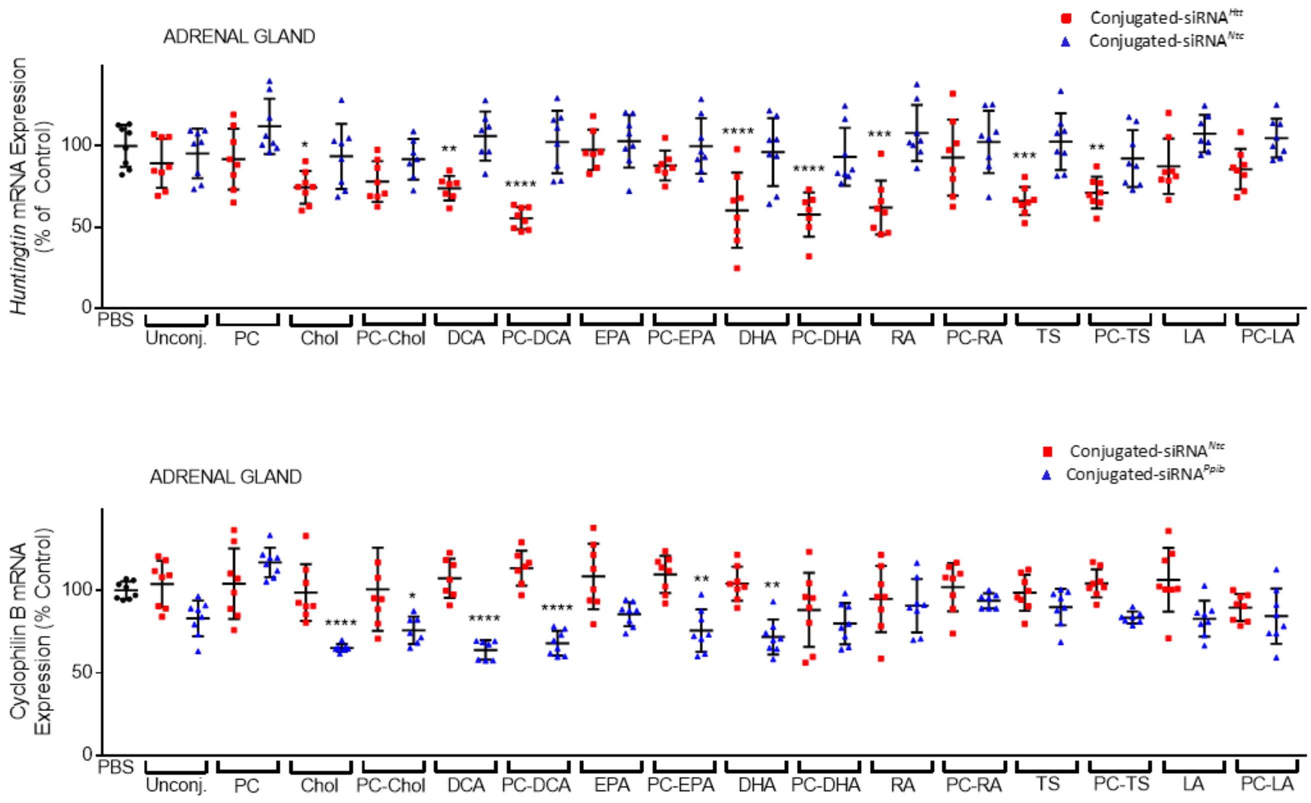

	PBS	Unconj.	PC	Chol	PC-Chol	DCA	PC-DCA	EPA	PC-EPA	DHA	PC-DHA	RA	PC-RA	TS	PC-TS	LA	PC-LA
**Huntingtin mRNA silencing (% compared to *Ntc*)**	0 ± 13	6 ± 15	20 ± 19	19 ± 10	14 ± 13	23 ± 12	47 ± 7	5 ± 12	12 ± 9	36 ± 23	36 ± 14	46 ± 16	10 ± 23	37 ± 9	21 ± 10	20 ± 17	19 ± 13
**Significance (compared to *Ntc*)**	/	ns	ns	ns	ns	*	****	ns	ns	*	*	****	ns	**	ns	ns	ns
**Cyclophilin B mRNA silencing (% compared to *Ntc*)**	0 ± 5	21 ± 11	0 ± 9	34 ± 2	25 ± 8	46 ± 6	48 ± 7	23 ± 7	34 ± 13	32 ± 11	8 ± 13	4 ± 16	8 ± 5	9 ± 11	21 ± 4	24 ± 11	5 ± 17
**Significance (compared to *Ntc*)**	/	ns	ns	**	ns	****	****	ns	***	**	ns	ns	ns	ns	ns	ns	ns

Supplementary Figure 25: Efficacy of conjugated siRNAs in adrenal glands. Subcutaneous injection (FVB/N mice); 20 mg/kg; collection of tissues one week after injection; n = 16 per gene and per conjugate (included non-targeting controls or *Ntc*). Huntingtin (*Htt*) (upper panel) and Cyclophilin B (*Ppib*) (lower panel) mRNA levels were measured using QuantiGene® (Affymetrix), normalized to a housekeeping gene, *Hprt* (Hypoxanthine-guanine phosphoribosyl transferase), and presented as percent of PBS (Phosphate buffered saline) control (mean ± SD). Data analysis: Outliners define with Grubb's method (alpha = 0.1%); Multiple comparisons = One-way ANOVA, Bonferroni test (****P<0.0001, ***P<0.001, **P<0.01, *P<0.1). The table indicates the average of inhibition percentages (n = 8) and significances for each target and conjugate compared to *Ntc* (mean ± SD; ns = non-significant).

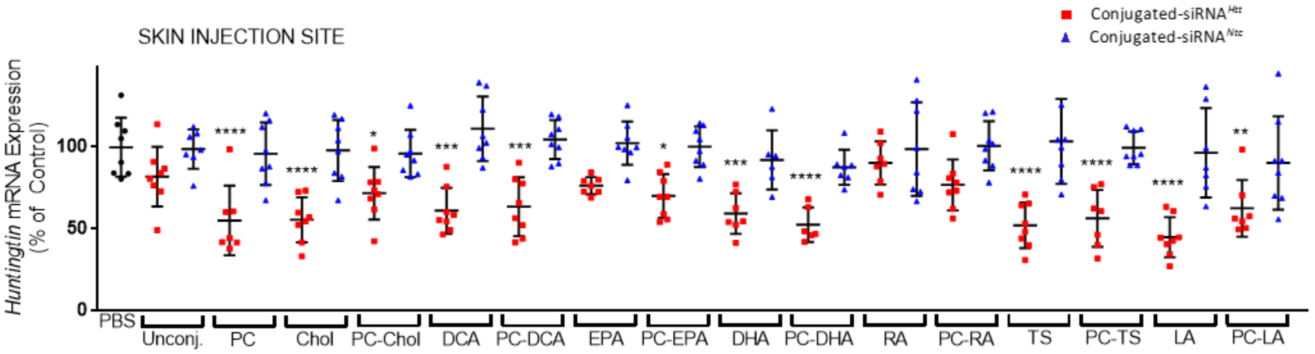

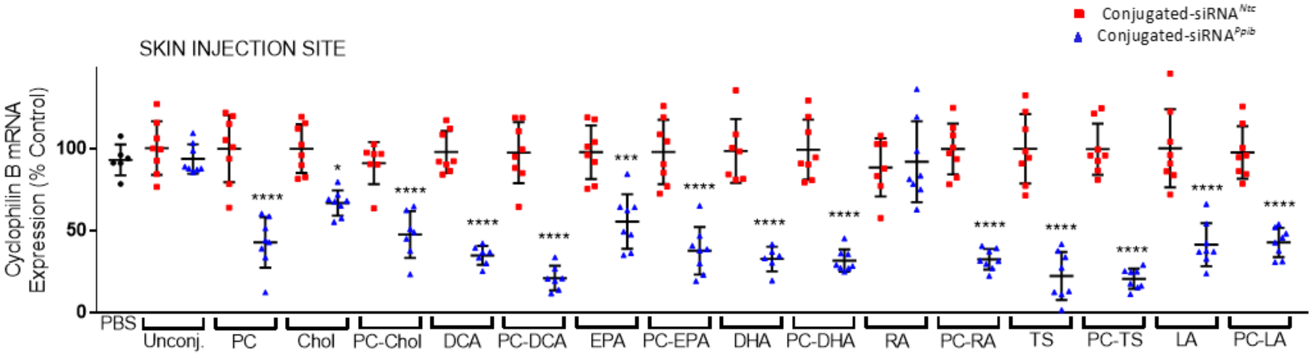

	PBS	Unconj.	PC	Chol	PC-Chol	DCA	PC-DCA	EPA	PC-EPA	DHA	PC-DHA	RA	PC-RA	TS	PC-TS	LA	PC-LA
**Huntingtin mRNA silencing (% compared to *Ntc*)**	0 ± 18	17 ± 18	41 ± 21	42 ± 14	24 ± 16	50 ± 14	41 ± 18	26 ± 5	30 ± 13	33 ± 12	35 ± 11	9 ± 13	24 ± 16	51 ± 14	43 ± 17	52 ± 12	28 ± 17
**Significance (compared to *Ntc*)**	/	ns	**	***	*	****	**	ns	*	**	**	ns	ns	****	***	****	ns
**Cyclophilin B mRNA silencing (% compared to *Ntc*)**	0 ± 9	7 ± 9	58 ± 15	33 ± 8	44 ± 14	63 ± 6	77 ± 7	43 ± 17	60 ± 15	66 ± 8	68 ± 7	0 ± 25	67 ± 6	78 ± 15	79 ± 6	59 ± 13	55 ± 9
**Significance (compared to *Ntc*)**	/	ns	****	**	***	****	****	****	****	****	****	ns	****	****	****	****	****

Supplementary Figure 26: Efficacy of conjugated siRNAs in skin (site of injection). Subcutaneous injection (FVB/N mice); 20 mg/kg; collection of tissues one week after injection; n = 16 per gene and per conjugate (included non-targeting controls or *Ntc*). Huntingtin (*Htt*) (upper panel) and Cyclophilin B (*Ppib*) (lower panel) mRNA levels were measured using QuantiGene® (Affymetrix), normalized to a housekeeping gene, *Hprt* (Hypoxanthine-guanine phosphoribosyl transferase), and presented as percent of PBS (Phosphate buffered saline) control (mean ± SD). Data analysis: Outliners define with Grubb's method (alpha = 0.1%); Multiple comparisons = One-way ANOVA, Bonferroni test (****P<0.0001, ***P<0.001, **P<0.01, *P<0.1). The table indicates the average of inhibition percentages (n = 8) and significances for each target and conjugate compared to *Ntc* (mean ± SD; ns = non-significant).

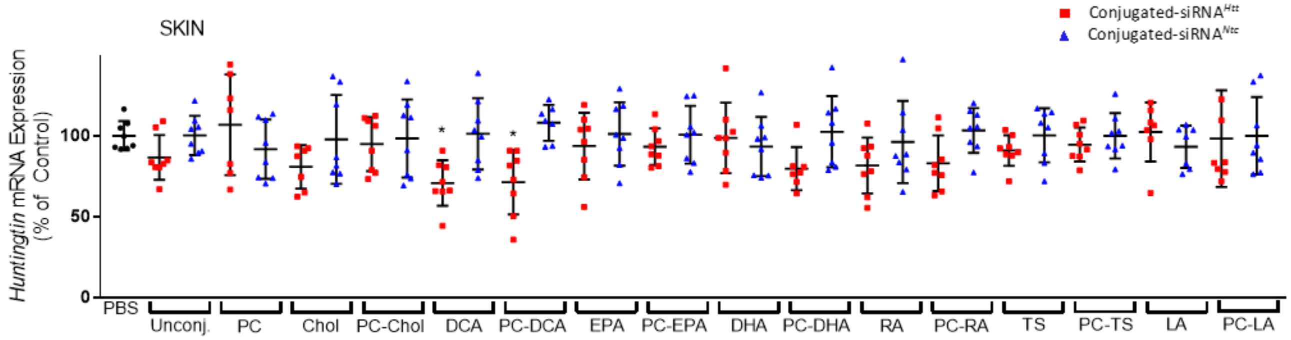

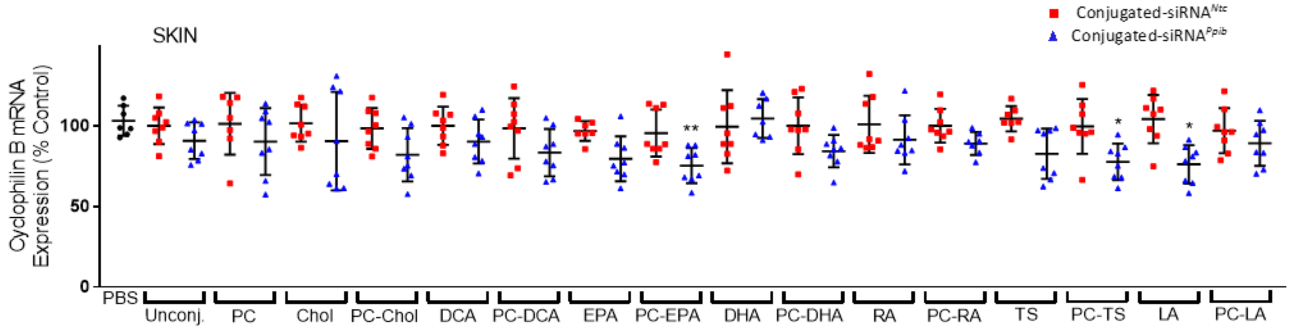

	PBS	Unconj.	PC	Chol	PC-Chol	DCA	PC-DCA	EPA	PC-EPA	DHA	PC-DHA	RA	PC-RA	TS	PC-TS	LA	PC-LA
Huntingtin mRNA silencing (% compared to *Ntc*)	0 ± 9	14 ± 14	0 ± 31	17 ± 13	4 ± 17	31 ± 14	37 ± 20	8 ± 21	7 ± 11	0 ± 22	23 ± 13	15 ± 17	20 ± 17	9 ± 10	5 ± 11	0 ± 18	2 ± 30
Significance (compared to *Ntc*)	/	ns	ns	ns	ns	ns	*	ns	ns	ns	ns	ns	ns	ns	ns	ns	ns
Cyclophilin B mRNA silencing (% compared to *Ntc*)	0 ± 9	9 ± 11	11 ± 21	11 ± 30	16 ± 17	10 ± 14	15 ± 15	27 ± 14	20 ± 11	0 ± 12	16 ± 10	10 ± 15	11 ± 7	22 ± 16	22 ± 11	28 ± 12	8 ± 14
Significance (compared to *Ntc*)	/	ns	ns	ns	ns	ns	ns	ns	ns	ns	ns	ns	ns	ns	ns	ns	ns

Supplementary Figure 27: Efficacy of conjugated siRNAs in skin. Subcutaneous injection (FVB/N mice); 20 mg/kg; collection of tissues one week after injection; n = 16 per gene and per conjugate (included non-targeting controls or *Ntc*). Huntingtin (*Htt*) (upper panel) and Cyclophilin B (*Ppib*) (lower panel) mRNA levels were measured using QuantiGene® (Affymetrix), normalized to a housekeeping gene, *Hprt* (Hypoxanthine-guanine phosphoribosyl transferase), and presented as percent of PBS (Phosphate buffered saline) control (mean ± SD). Data analysis: Outliners define with Grubb's method (alpha = 0.1%); Multiple comparisons = One-way ANOVA, Bonferroni test (**P<0.01, *P<0.1). The table indicates the average of inhibition percentages (n = 8) and significances for each target and conjugate compared to *Ntc* (mean ± SD; ns = non-significant).

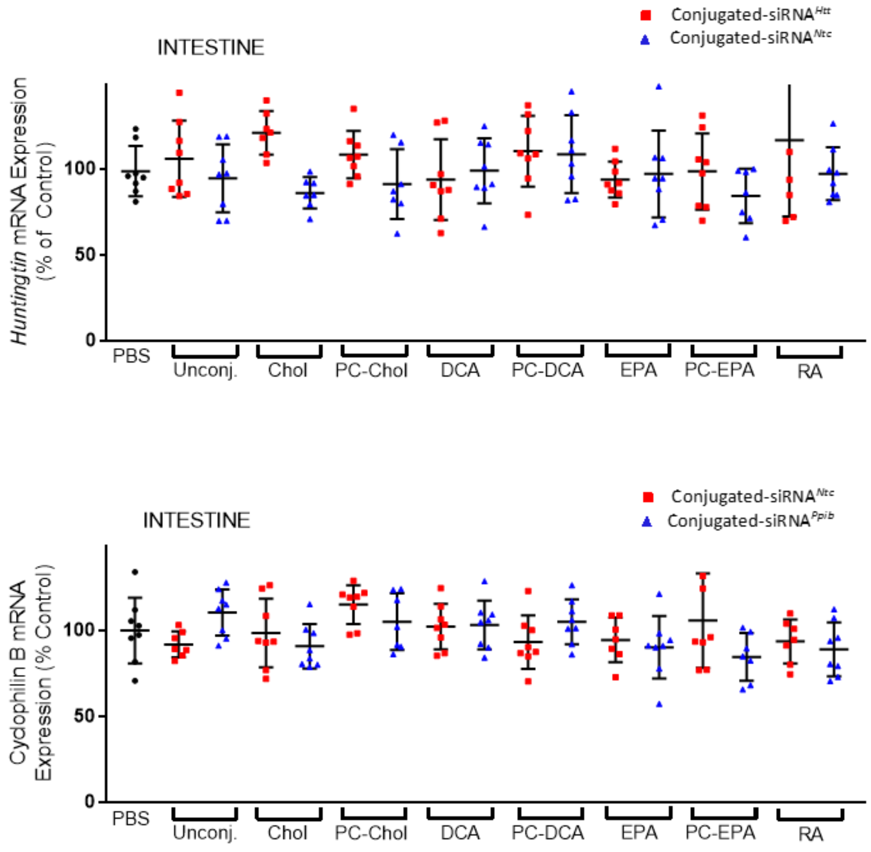

	PBS	Unconj.	Chol	PC-Chol	DCA	PC-DCA	EPA	PC-EPA	RA
**Huntingtin mRNA silencing (% compared to *Ntc*)**	0 ± 15	0 ± 22	0 ± 13	0 ± 14	5 ± 23	0 ± 21	3 ± 11	0 ± 22	0 ± 44
**Significance (compared to *Ntc*)**	/	ns	ns	ns	ns	ns	ns	ns	ns
**Cyclophilin B mRNA silencing (% compared to *Ntc*)**	0 ± 19	0 ± 13	8 ± 16	10 ± 13	0 ± 14	0 ± 13	4 ± 18	21 ± 14	5 ± 15
**Significance (compared to *Ntc*)**	/	ns	ns	ns	ns	ns	ns	ns	ns

Supplementary Figure 28: Efficacy of conjugated siRNAs in intestine. Subcutaneous injection (FVB/N mice); 20 mg/kg; collection of tissues one week after injection; n = 16 per gene and per conjugate (included non-targeting controls or *Ntc*). Huntingtin (*Htt*) (upper panel) and Cyclophilin B (*Ppib*) (lower panel) mRNA levels were measured using QuantiGene® (Affymetrix), normalized to a housekeeping gene, *Hprt* (Hypoxanthine-guanine phosphoribosyl transferase), and presented as percent of PBS (Phosphate buffered saline) control (mean ± SD). Data analysis: Outliners define with Grubb's method (alpha = 0.1%); Multiple comparisons = One-way ANOVA, Bonferroni test. The table indicates the average of inhibition percentages (n = 8) and significances for each target and conjugate as percent of *Ntc* (mean ± SD; ns = non-significant).

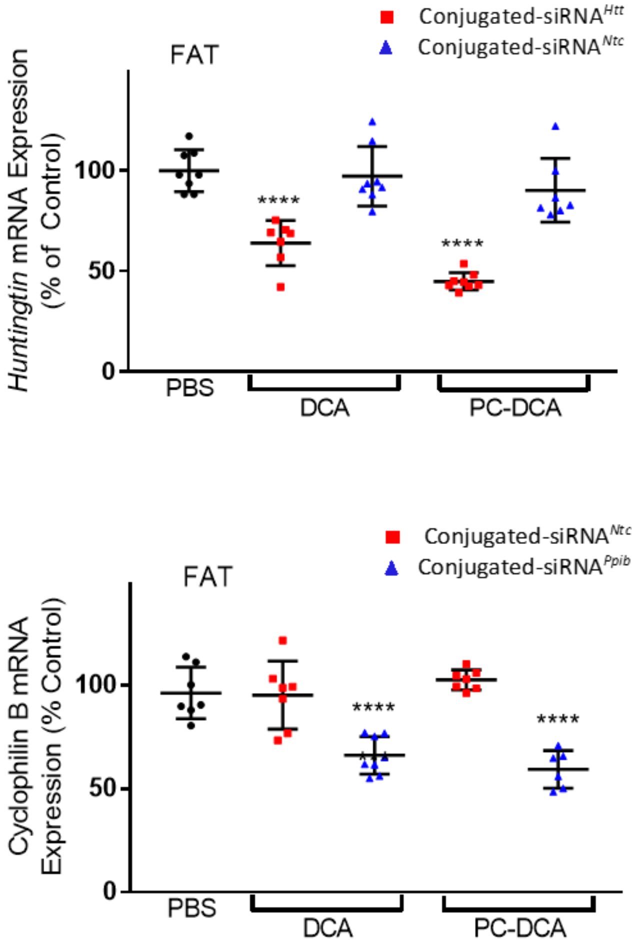

	PBS	DCA	PC-DCA
Huntingtin mRNA silencing (% compared to *Ntc*)	0 ± 10	30 ± 18	51 ± 4
Significance (compared to *Ntc*)	/	****	****
Cyclophilin B mRNA silencing (% compared to *Ntc*)	0 ± 16	35 ± 9	31 ± 20
Significance (compared to *Ntc*)	/	***	****

Supplementary Figure 29: Efficacy of conjugated siRNAs in fat. Subcutaneous injection (FVB/N mice); 20 mg/kg; collection of tissues one week after injection; n = 16 per gene and per conjugate (included non-targeting controls or *Ntc*). Huntingtin (*Htt*) (upper panel) and Cyclophilin B (*Ppib*) (lower panel) mRNA levels were measured using QuantiGene® (Affymetrix), normalized to a housekeeping gene, *Hprt* (Hypoxanthine-guanine phosphoribosyl transferase), and presented as percent of PBS (Phosphate buffered saline) control (mean ± SD). Data analysis: Outliners define with Grubb's method (alpha = 0.1%); Multiple comparisons = One-way ANOVA, Bonferroni test (****P<0.0001, ***P<0.001). The table indicates the average of inhibition percentages (n = 8) and significances for each target using DCA and PC-DCA conjugated siRNAs compared to *Ntc* (mean ± SD).

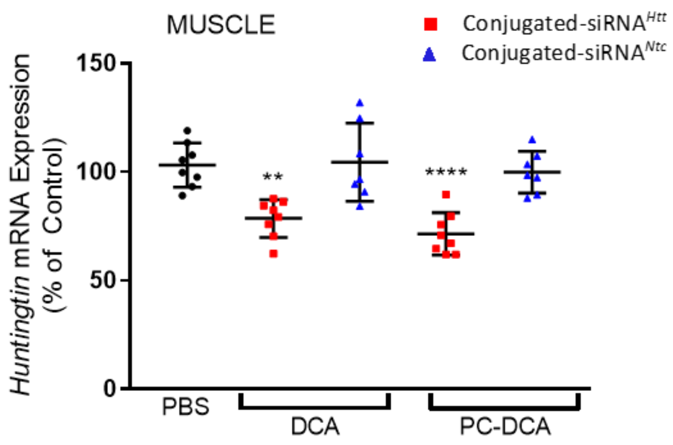

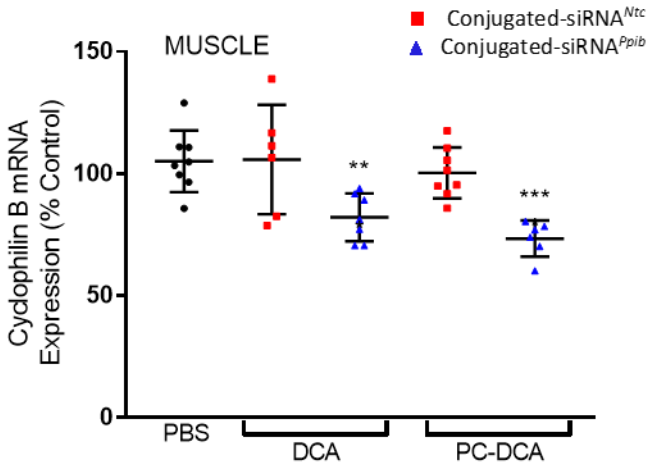

	PBS	DCA	PC-DCA
Huntingtin mRNA silencing (% compared to *Ntc*)	0 ± 10	21 ± 9	24 ± 10
Significance (compared to *Ntc*)	/	**	***
Cyclophilin B mRNA silencing (% compared to *Ntc*)	0 ± 13	29 ± 16	30 ± 11
Significance (compared to *Ntc*)	/	ns	***

Supplementary Figure 30: Efficacy of conjugated siRNAs in muscle. Subcutaneous injection (FVB/N mice); 20 mg/kg; collection of tissues one week after injection; n = 16 per gene and per conjugate (included non-targeting controls or *Ntc*). Huntingtin (*Htt*) (upper panel) and Cyclophilin B (*Ppib*) (lower panel) mRNA levels were measured using QuantiGene® (Affymetrix), normalized to a housekeeping gene, *Hprt* (Hypoxanthine-guanine phosphoribosyl transferase), and presented as percent of PBS (Phosphate buffered saline) control (mean ± SD). Data analysis: Outliners define with Grubb's method (alpha = 0.1%); Multiple comparisons = One-way ANOVA, Bonferroni test (****P<0.0001, ***P<0.001, **P<0.01). The table indicates the average of inhibition percentages (n = 8) and significances for each target using DCA and PC-DCA conjugated siRNAs compared to *Ntc* (mean ± SD ; ns = non-significant).

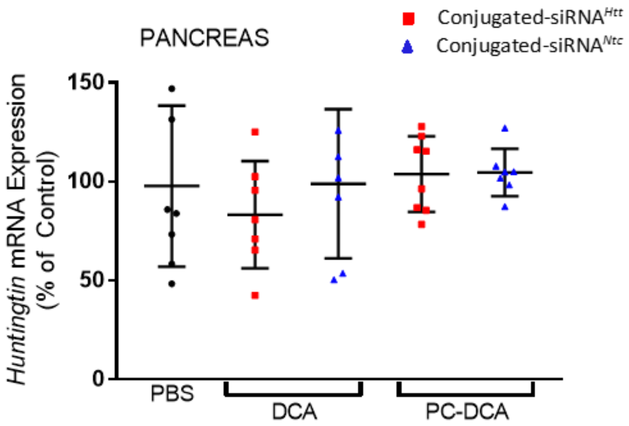

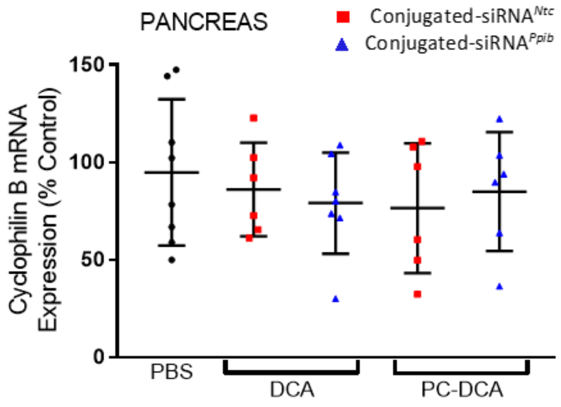

	PBS	DCA	PC-DCA
**Huntingtin mRNA silencing (% compared to *Ntc*)**	0 ± 41	16 ± 17	0 ± 19
**Significance (compared to *Ntc*)**	/	ns	ns
**Cyclophilin B mRNA silencing (% compared to *Ntc*)**	0 ± 37	7 ± 26	0 ± 30
**Significance (compared to *Ntc*)**	/	ns	ns

Supplementary Figure 31: Efficacy of conjugated siRNAs in pancreas. Subcutaneous injection (FVB/N mice); 20 mg/kg; collection of tissues one week after injection; n = 16 per gene and per conjugate (included non-targeting controls or *Ntc*). Huntingtin (*Htt*) (upper panel) and Cyclophilin B (*Ppib*) (lower panel) mRNA levels were measured using QuantiGene® (Affymetrix), normalized to a housekeeping gene, *Hprt* (Hypoxanthine-guanine phosphoribosyl transferase), and presented as percent of PBS (Phosphate buffered saline) control (mean ± SD). Data analysis: Outliners define with Grubb’s method (alpha = 0.1%); Multiple comparisons = One-way ANOVA, Bonferroni test. The table indicates the average of inhibition percentages (n = 8) and significances for each target using DCA and PC-DCA conjugated siRNAs compared to *Ntc* (mean ± SD; ns = non-significant).

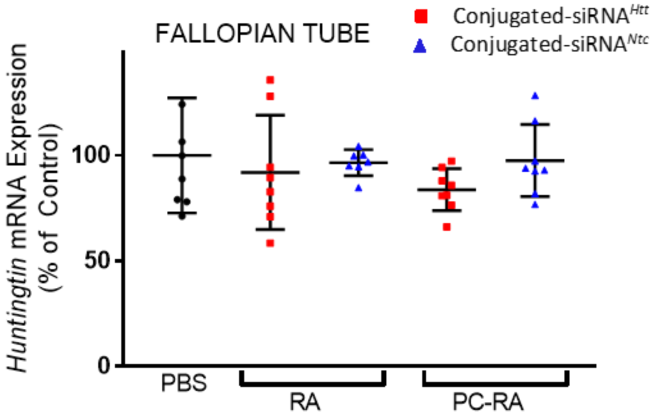

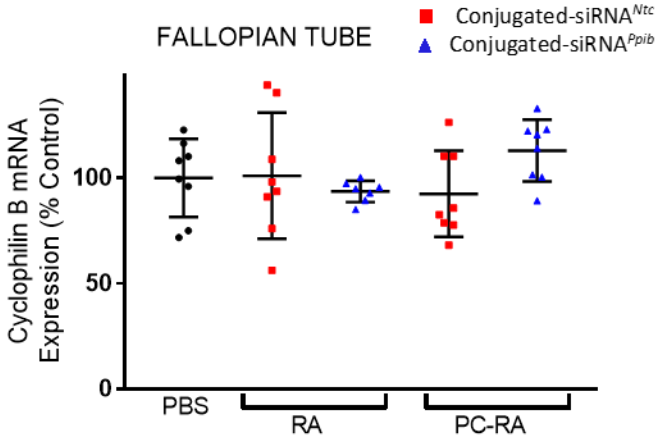

	PBS	RA	PC-RA
Huntingtin mRNA silencing (% compared to *Ntc*)	0 ± 27	5 ± 27	14 ± 10
Significance (compared to *Ntc*)	/	ns	ns
Cyclophilin B mRNA silencing (% compared to *Ntc*)	0 ± 18	7 ± 5	0 ± 15
Significance (compared to *Ntc*)	/	ns	ns

Supplementary Figure 32: Efficacy of conjugated siRNAs in fallopian tube. Subcutaneous injection (FVB/N mice); 20 mg/kg; collection of tissues one week after injection; n = 16 per gene and per conjugate (included non-targeting controls or *Ntc*). Huntingtin (*Htt*) (upper panel) and Cyclophilin B (*Ppib*) (lower panel) mRNA levels were measured using QuantiGene® (Affymetrix), normalized to a housekeeping gene, *Hprt* (Hypoxanthine-guanine phosphoribosyl transferase), and presented as percent of PBS (Phosphate buffered saline) control (mean ± SD). Data analysis: Outliners define with Grubb's method (alpha = 0.1%); Multiple comparisons = One-way ANOVA, Bonferroni test. The table indicates the average of inhibition percentages (n = 8) and significances for each target using RA and PC-RA conjugated siRNAs compared to *Ntc* (mean ± SD; ns = non-significant).

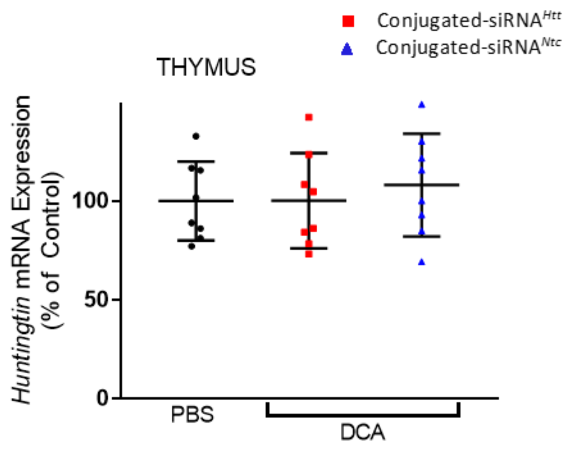

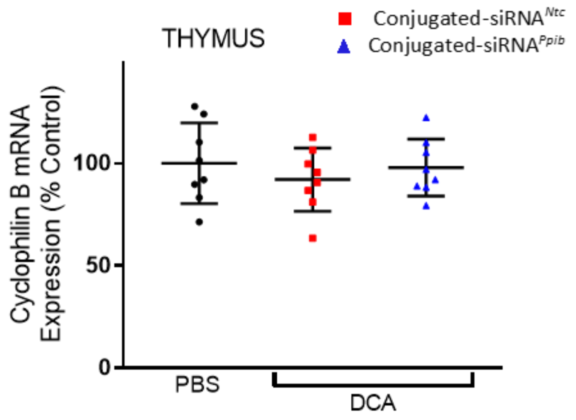

	PBS	DCA
Huntingtin mRNA silencing (% compared to *Ntc*)	0 ± 20	8 ± 24
Significance (compared to *Ntc*)	/	ns
Cyclophilin B mRNA silencing (% compared to *Ntc*)	0 ± 20	0 ± 14
Significance (compared to *Ntc*)	/	ns

Supplementary Figure 33: Efficacy of conjugated siRNAs in thymus. Subcutaneous injection (FVB/N mice); 20 mg/kg; collection of tissues one week after injection; n = 16 per gene and per conjugate (included non-targeting controls or *Ntc*). Huntingtin (*Htt*) (upper panel) and Cyclophilin B (*Ppib*) (lower panel) mRNA levels were measured using QuantiGene® (Affymetrix), normalized to a housekeeping gene, *Hprt* (Hypoxanthine-guanine phosphoribosyl transferase), and presented as percent of PBS (Phosphate buffered saline) control (mean ± SD). Data analysis: Outliners define with Grubb's method (alpha = 0.1%); Multiple comparisons = One-way ANOVA, Bonferroni test. The table indicates the average of inhibition percentages (n = 8) and significances for each target using DCA conjugated siRNAs compared to *Ntc* (mean ± SD; ns = non-significant).

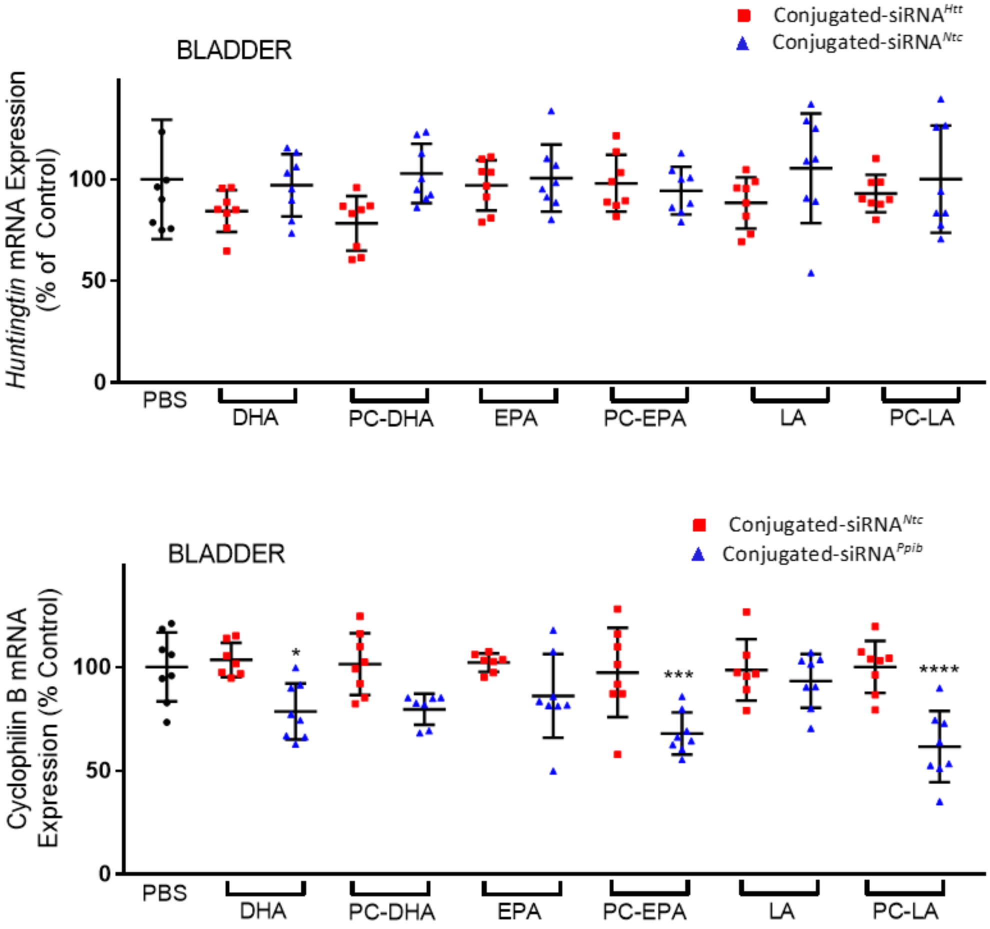

	PBS	DHA	PC-DHA	EPA	PC-EPA	LA	PC-LA
Huntingtin mRNA silencing (% compared to ***Ntc***)	0 ± 29	13 ± 10	25 ± 13	4 ± 12	0 ± 14	17 ± 13	7 ± 9
Significance (compared to ***Ntc***)	/	ns	ns	ns	ns	ns	ns
Cyclophilin B mRNA silencing (% compared to ***Ntc***)	0 ± 17	25 ± 14	22 ± 8	16 ± 20	30 ± 10	5 ± 13	39 ± 17
Significance (compared to ***Ntc***)	/	*	ns	**	*	ns	****

Supplementary Figure 34: Efficacy of conjugated siRNAs in bladder. Subcutaneous injection (FVB/N mice); 20 mg/kg; collection of tissues one week after injection; n = 16 per gene and per conjugate (included non-targeting controls or *Ntc*). Huntingtin (*Htt*) (upper panel) and Cyclophilin B (*Ppib*) (lower panel) mRNA levels were measured using QuantiGene® (Affymetrix), normalized to a housekeeping gene, *Hprt* (Hypoxanthine-guanine phosphoribosyl transferase), and presented as percent of PBS (Phosphate buffered saline) control (mean ± SD). Data analysis: Outliners define with Grubb's method (alpha = 0.1%); Multiple comparisons = One-way ANOVA, Bonferroni test (****P<0.0001, ***P<0.001, **P<0.01, *P<0.1). The table indicates the average of inhibition percentages (n = 8) and significances for each target and conjugates compared to *Ntc* (mean ± SD; ns = non-significant).

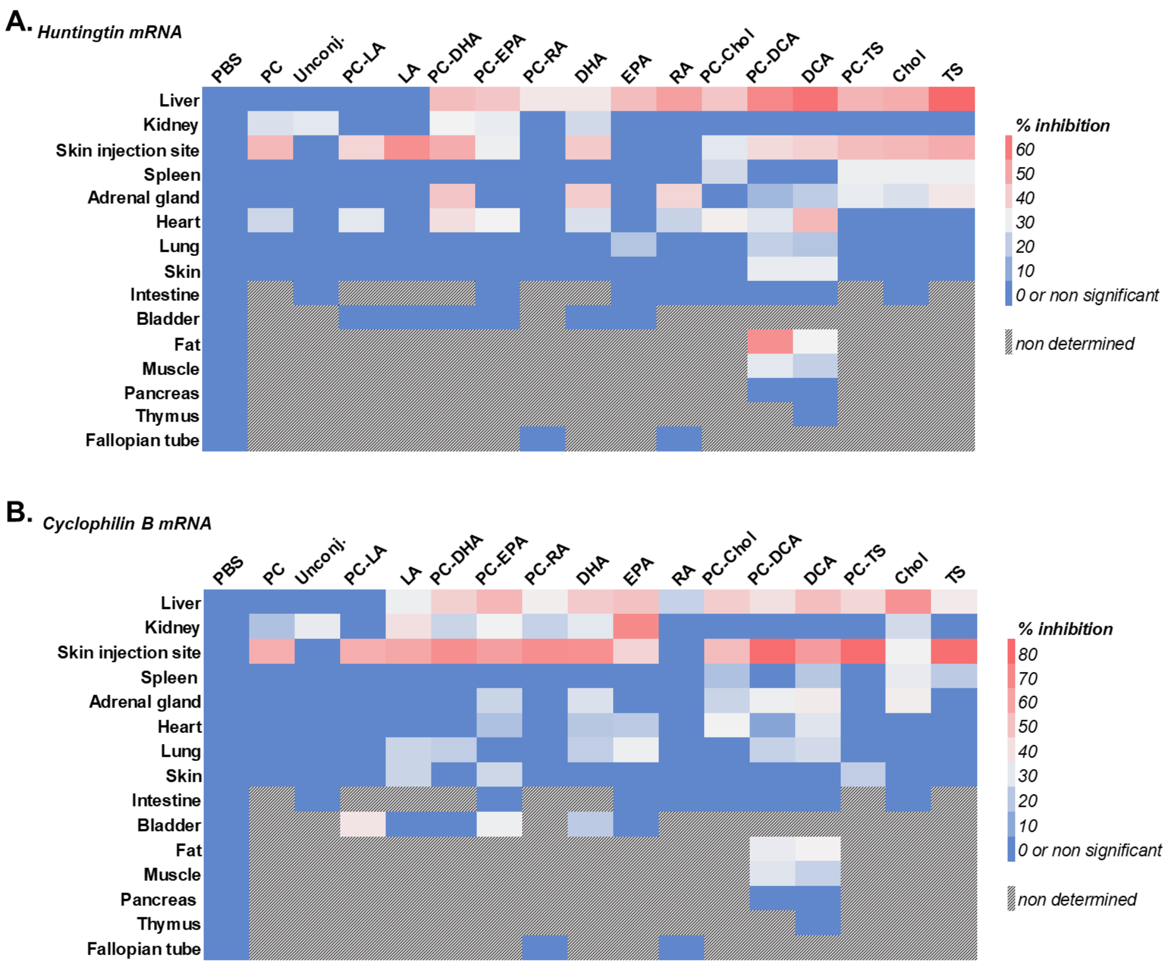

Supplementary Figure 35. Percentages of mRNA silencing depend on the conjugate attached to the siRNA. (A.) Target = Huntingtin mRNA (B.) Target = Cyclophilin B mRNA. Subcutaneous injection (FVB/N mice) ; 20 mg/kg ; collection of tissues 1 week after injection ; n = 16 per target and per conjugate (included non-targeting controls). mRNA levels were measured using QuantiGene® (Affymetrix), normalized to a housekeeping gene, *Hprt* (Hypoxanthine-guanine phosphoribosyl transferase), and presented as percent of PBS (Phosphate buffered saline) control (average of 8 mice).

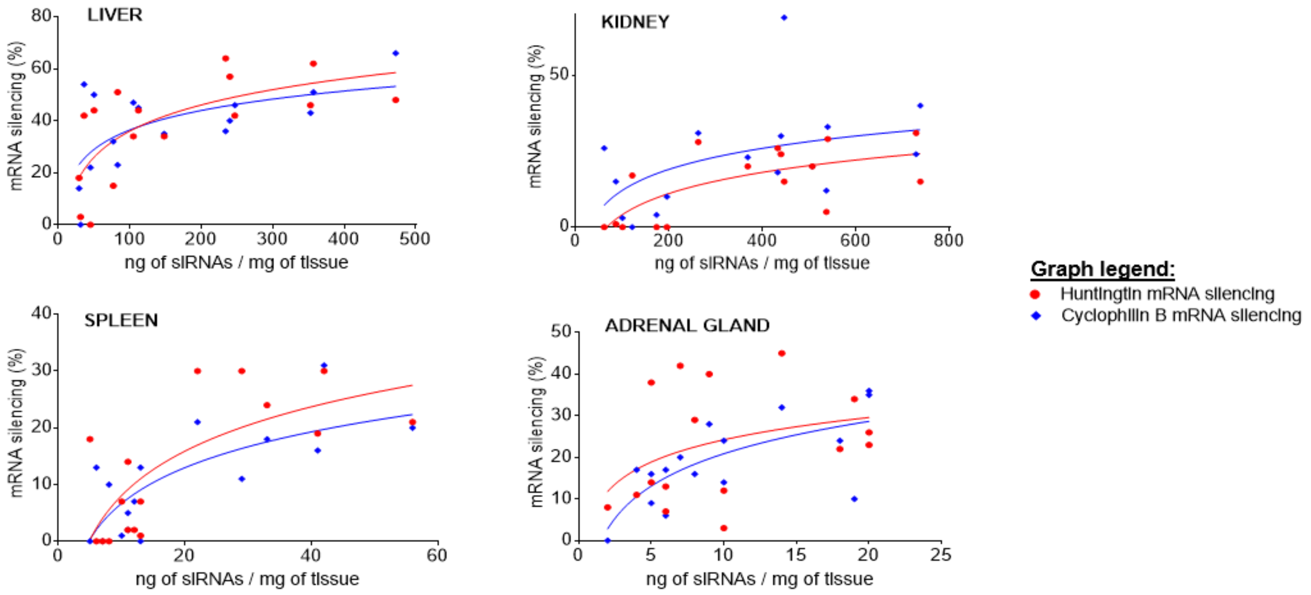

Supplementary Figure 36: Dot plots showing percent silencing of *Huntingtin* (red dots) or *Cyclophilin B* (blue dots) mRNAs by lipid-conjugated siRNAs plotted against tissue concentration of the siRNA in liver, kidney, spleen, and adrenal gland.

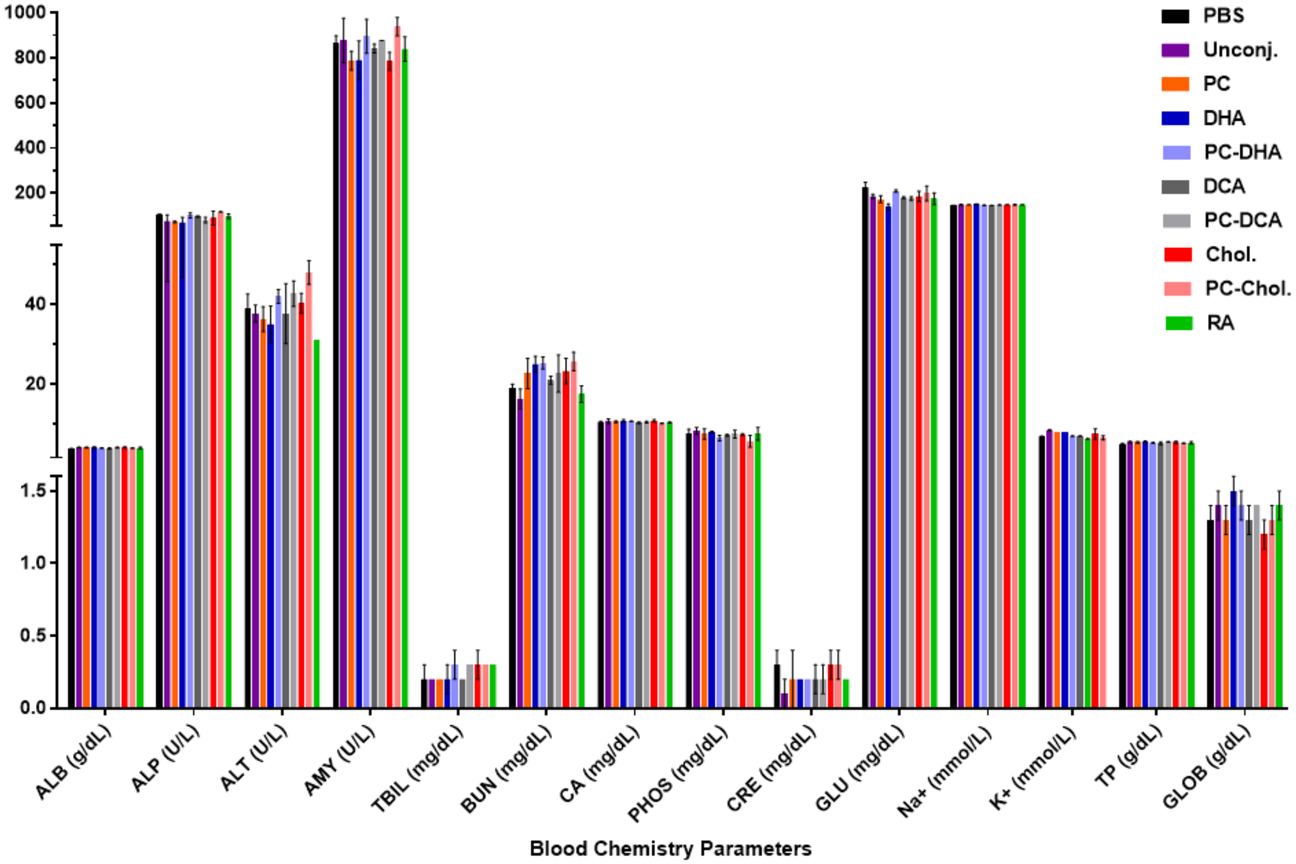

	PBS	Unconj.	PC	DHA	PC-DHA	DCA	PC-DCA	Chol.	PC-Chol.	RA
ALB (g/dL)	3.7 ± 0.2	4.1 ± 0.1	4.1 ± 0.1	4.1 ± 0.2	4.0 ± 0.1	3.9 ± 0.2	4.1 ± 0.1	4.1 ± 0.2	4.0 ± 0.1	4.0 ± 0.2
ALP (U/L)	105.3 ± 1.5	74.7 ± 29.0	72.0 ± 3.5	69.3 ± 22.5	101.0 ± 11.3	96.0 ± 3.0	80.3 ± 12.1	89.3 ± 30.1	116.7 ± 1.5	97.0 ± 11.3
ALT (U/L)	39.0 ± 3.6	37.7 ± 2.1	36.3 ± 3.1	35.0 ± 4.6	42.0 ± 1.7	37.7 ± 7.5	42.7 ± 3.2	40.3 ± 2.5	48.0 ± 3.0	31.0 ± 0.0
AMY (U/L)	864.3 ± 33.5	877.0 ± 99.0	788.0 ± 41.7	789.7 ± 85.2	895.7 ± 75.4	841.7 ± 19.5	876.6 ± 0.7	785.7 ± 39.5	938.0 ± 41.0	839.0 ± 53.7
TBIL (mg/dL)	0.2 ± 0.1	0.2 ± 0.0	0.2 ± 0.0	0.2 ± 0.1	0.3 ± 0.1	0.2 ± 0.0	0.3 ± 0.0	0.3 ± 0.1	0.3 ± 0.0	0.3 ± 0.0
BUN (mg/dL)	19.0 ± 1.0	16.3 ± 2.5	22.7 ± 3.8	25.0 ± 2.0	25.3 ± 1.5	21.0 ± 1.0	22.7 ± 4.7	23.3 ± 3.2	25.7 ± 2.3	17.5 ± 2.1
CA (mg/dL)	10.5 ± 0.2	10.7 ± 0.6	10.6 ± 0.2	10.8 ± 0.3	10.7 ± 0.1	10.3 ± 0.2	10.5 ± 0.2	10.8 ± 0.3	10.2 ± 0.1	10.4 ± 0.1
PHOS (mg/dL)	7.7 ± 1.0	8.3 ± 0.9	7.5 ± 1.3	8.0 ± 0.2	6.5 ± 0.7	7.2 ± 0.2	7.5 ± 1.0	7.4 ± 0.2	5.7 ± 1.5	7.6 ± 1.6
CRE (mg/dL)	0.3 ± 0.1	0.1 ± 0.1	0.2 ± 0.2	0.2 ± 0.2	0.2 ± 0.0	0.2 ± 0.0	0.2 ± 0.1	0.3 ± 0.1	0.3 ± 0.1	0.2 ± 0.0
GLU (mg/dL)	225.7 ± 22.4	185.7 ± 9.3	172.7 ± 15.3	138.3 ± 14.0	209.0 ± 6.2	180.0 ± 3.5	175.7 ± 8.1	185.3 ± 23.5	199.0 ± 33.2	175.0 ± 25.5
Na+ (mmol/L)	144.7 ± 1.5	148.3 ± 1.5	148.3 ± 0.6	150.0 ± 2.0	146.0 ± 1.0	145.3 ± 1.5	147.3 ± 0.6	148.0 ± 1.0	148.0 ± 2.0	148.5 ± 0.7
K+ (mmol/L)	6.9 ± 0.1	8.5 ± 0.1	7.9 ± 0.0	8.0 ± 0.0	7.0 ± 0.1	7.0 ± 0.1	/	7.5 ± 1.3	6.6 ± 0.5	6.3 ± 0.1
TP (g/dL)	5.0 ± 0.2	5.5 ± 0.2	5.4 ± 0.2	5.6 ± 0.2	5.3 ± 0.1	5.2 ± 0.3	5.5 ± 0.1	5.4 ± 0.3	5.2 ± 0.1	5.3 ± 0.3
GLOB (g/dL)	1.3 ± 0.1	1.4 ± 0.1	1.3 ± 0.1	1.5 ± 0.1	1.4 ± 0.1	1.3 ± 0.1	1.4 ± 0.0	1.2 ± 0.1	1.3 ± 0.1	1.4 ± 0.1

Supplementary Figure 37. Blood chemistry parameters for conjugated siRNAs treated mice. Subcutaneous injection (FVB/N mice); 20 mg/kg; collection of blood one week after injection; n = 3 per group. ALB = Albumin; ALP = Alkaline Phosphatase; ALT = Alanine Aminotransferase; AMY = Amylase; TBIL = Total Bilirubin; BUN = Blood Urea Nitrogen; CA = Calcium; PHOS = Phosphate; CRE = Creatinine; GLU = Glucose; Na+ = Sodium; K+ = Potassium; TP = Total Protein; GLOB = Globulin.
